# Supplementary material for: Biobased chiral semi-crystalline or amorphous high-performance polyamides and their scalable stereoselective synthesis
Source: Nat Commun. 2020 Jan 24;11:509. doi: 10.1038/s41467-020-14361-6 (PMC6981233; doi:10.1038/s41467-020-14361-6)
Supplement: Supplementary file 1 — Supplementary Information [file 41467_2020_14361_MOESM1_ESM.pdf]

Supplementary Information

V. Sieber et al.

Biobased Chiral Semi-Crystalline or Amorphous High-Performance Polyamides and their Scalable Stereoselective Synthesis

1. Supplementary Figures

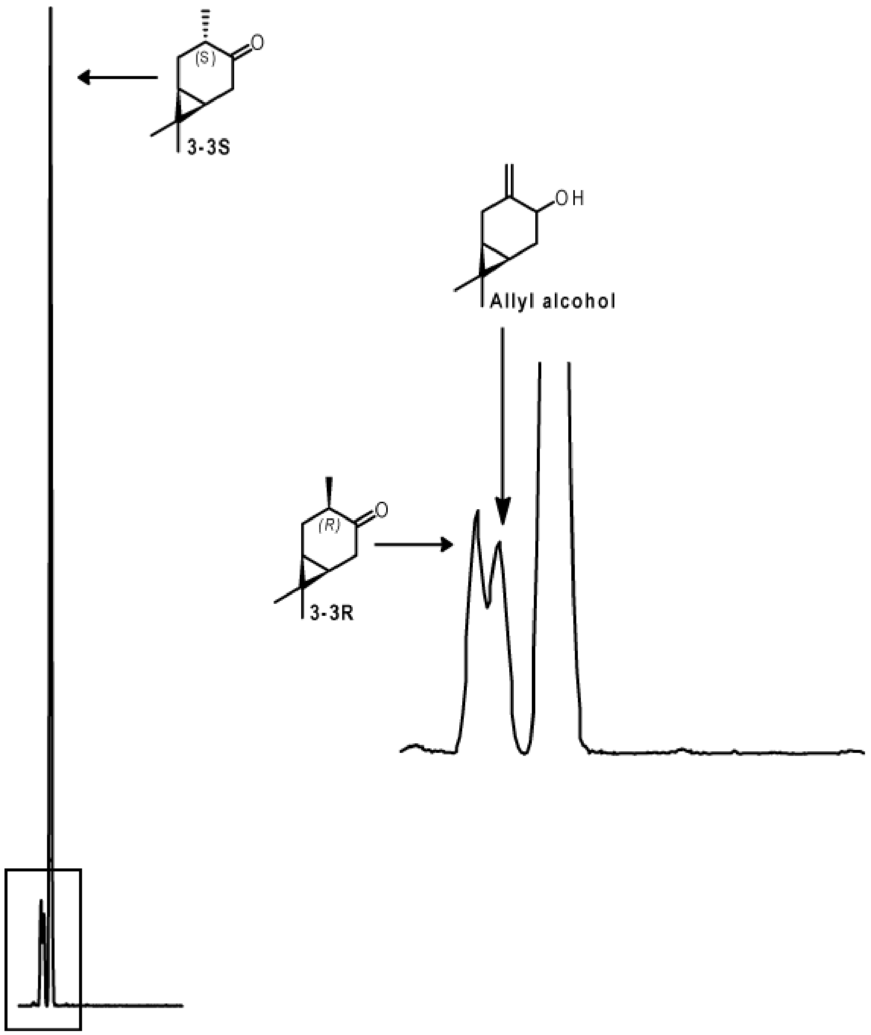

Supplementary Figure 1: Isomeric ketones **3-3S** and **3-3R** and the intermediate allyl alcohol.

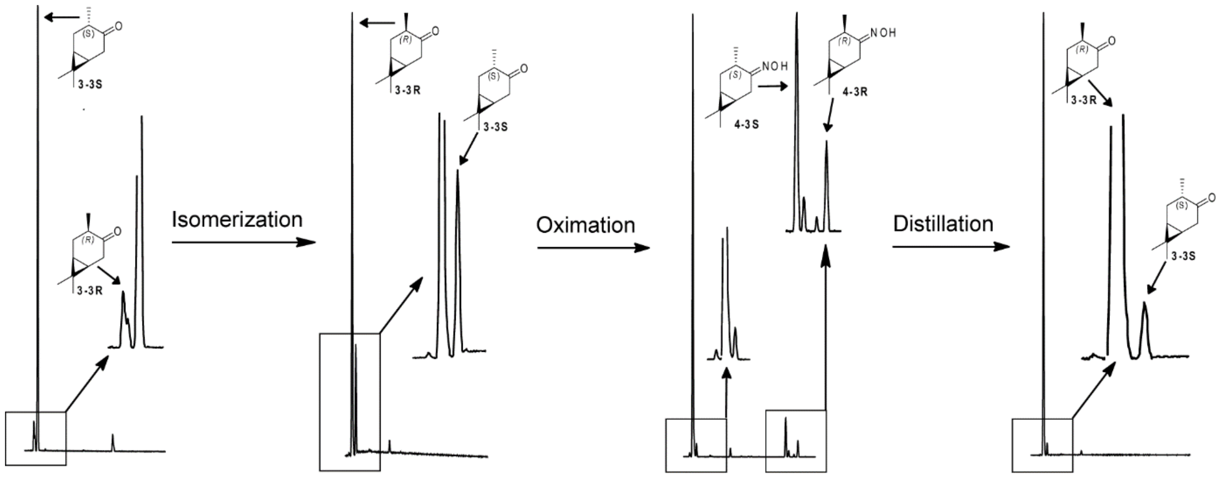

Supplementary Figure 2: GCMS-spectra of the enrichment of ketone **3-3R** by acidic isomerization of ketone **3-3S**, kinetic resolution by oximation with $HONH_2 \cdot HCl$ and subsequent distillation.

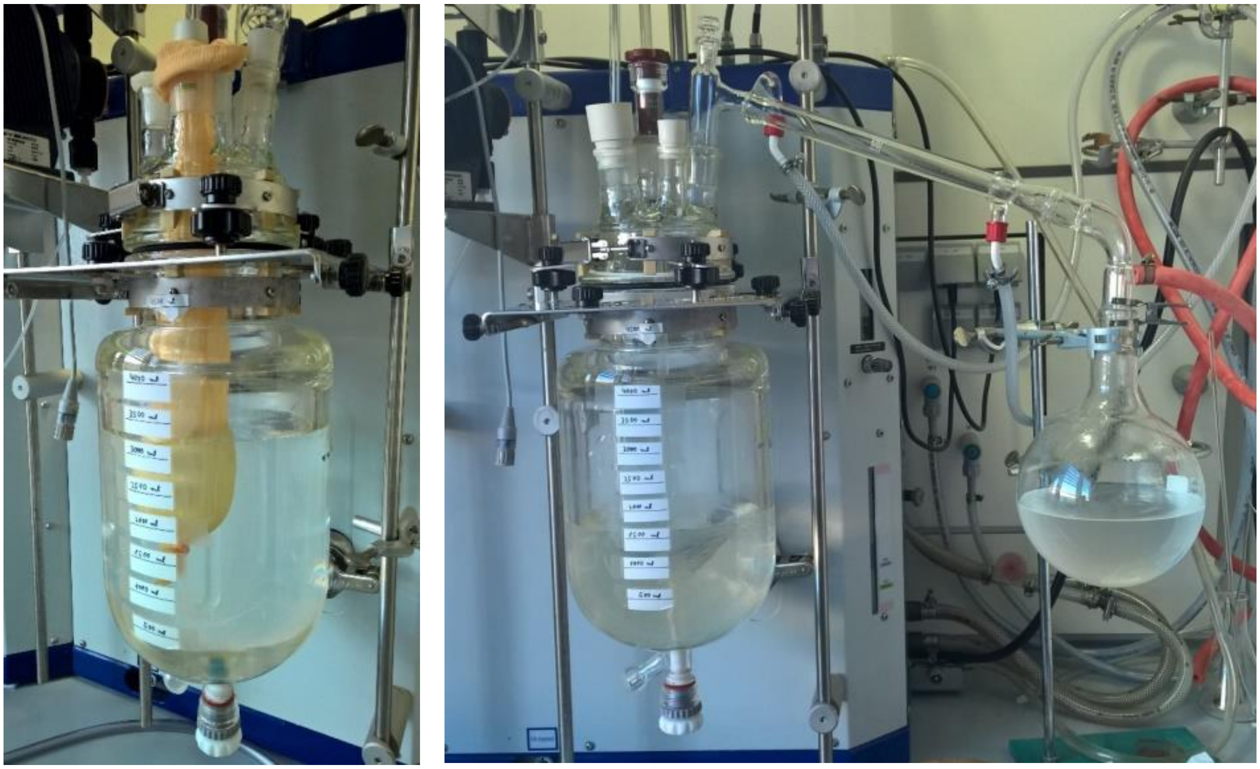

Supplementary Figure 3: Enzyme bag loaded with Novozyme-435 for epoxidation of (+)-3-carene (**1**) to epoxide **2-3S** (left) and azeotropic distillation of cyclohexane before the Meinwald rearrangement of epoxide **2-3S** to ketone **3-3S** catalysed by $Fe(ClO_4)_2 \cdot H_2O$ (right) in the 4.0 L scale.

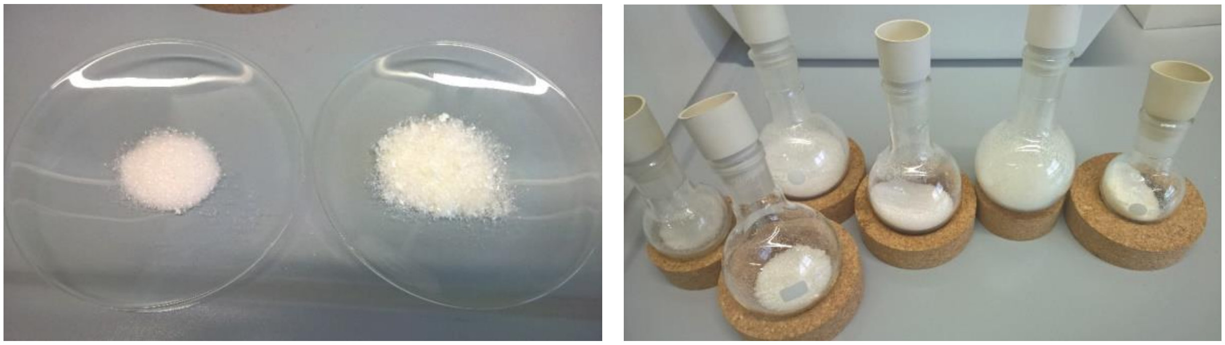

Supplementary Figure 4: Lactam **5-3S** synthesized in the 4.0 L scale after crystallization at -20 °C.

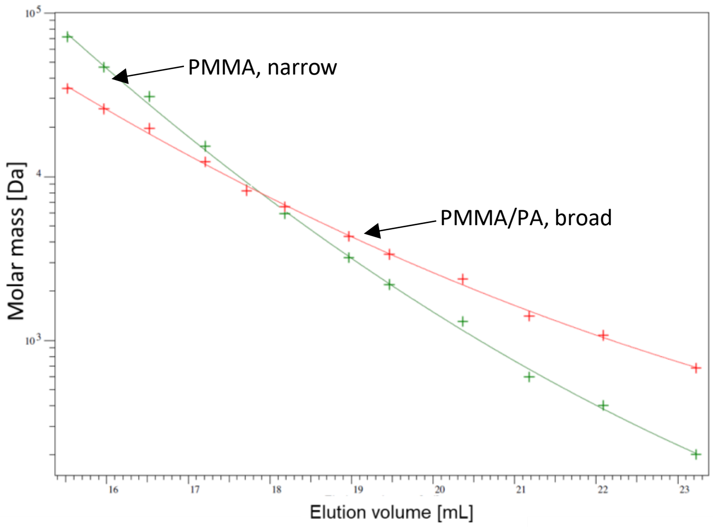

Supplementary Figure 5: Calibration curve for GPC measurements with narrow PMMA calibration (green) and PMMA/PA broad calibration (red).

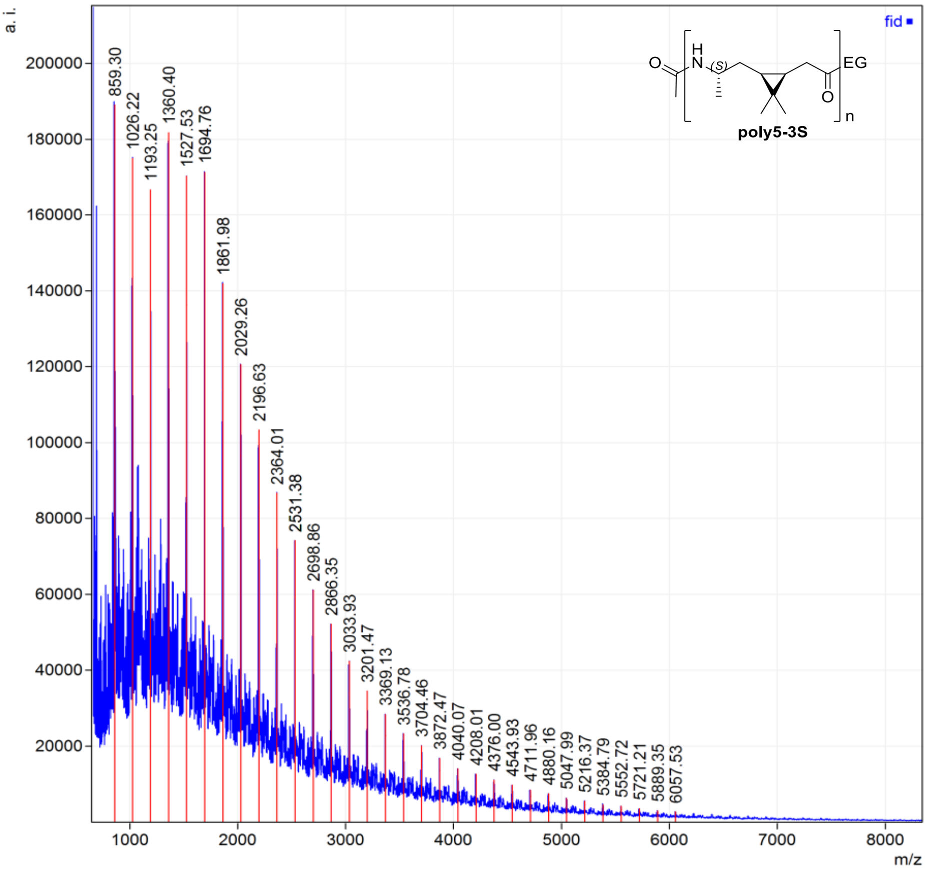

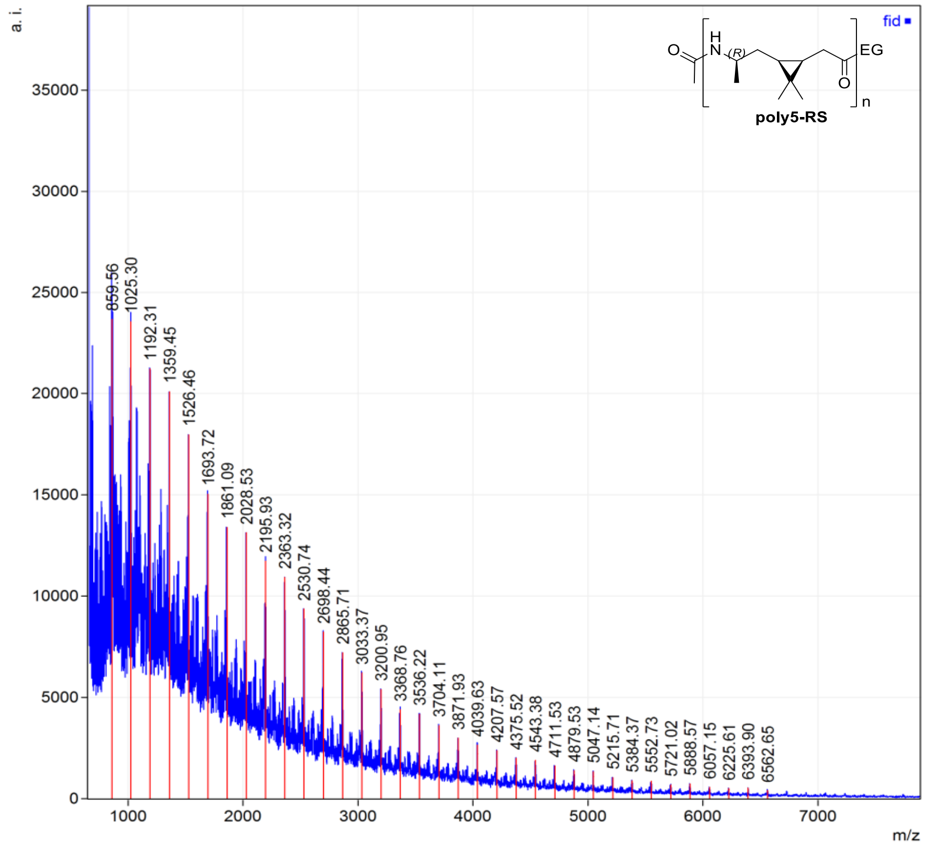

Supplementary Figure 6: MALDI-TOF measurements of **poly5-3S** and **poly5-3R**.

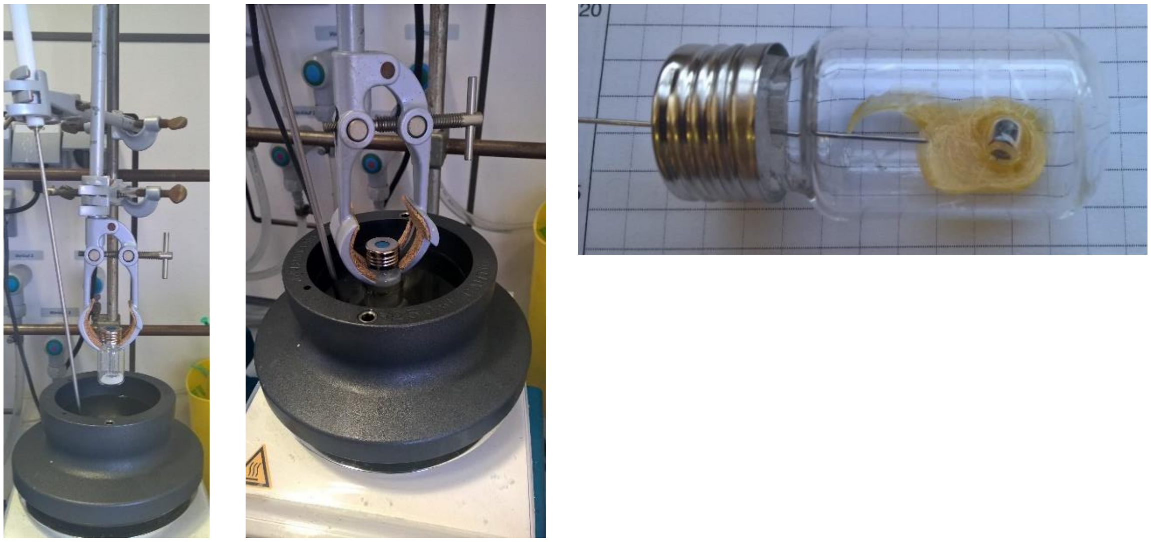

Supplementary Figure 7: Pictures of polymerizations under application of polymerization method A.

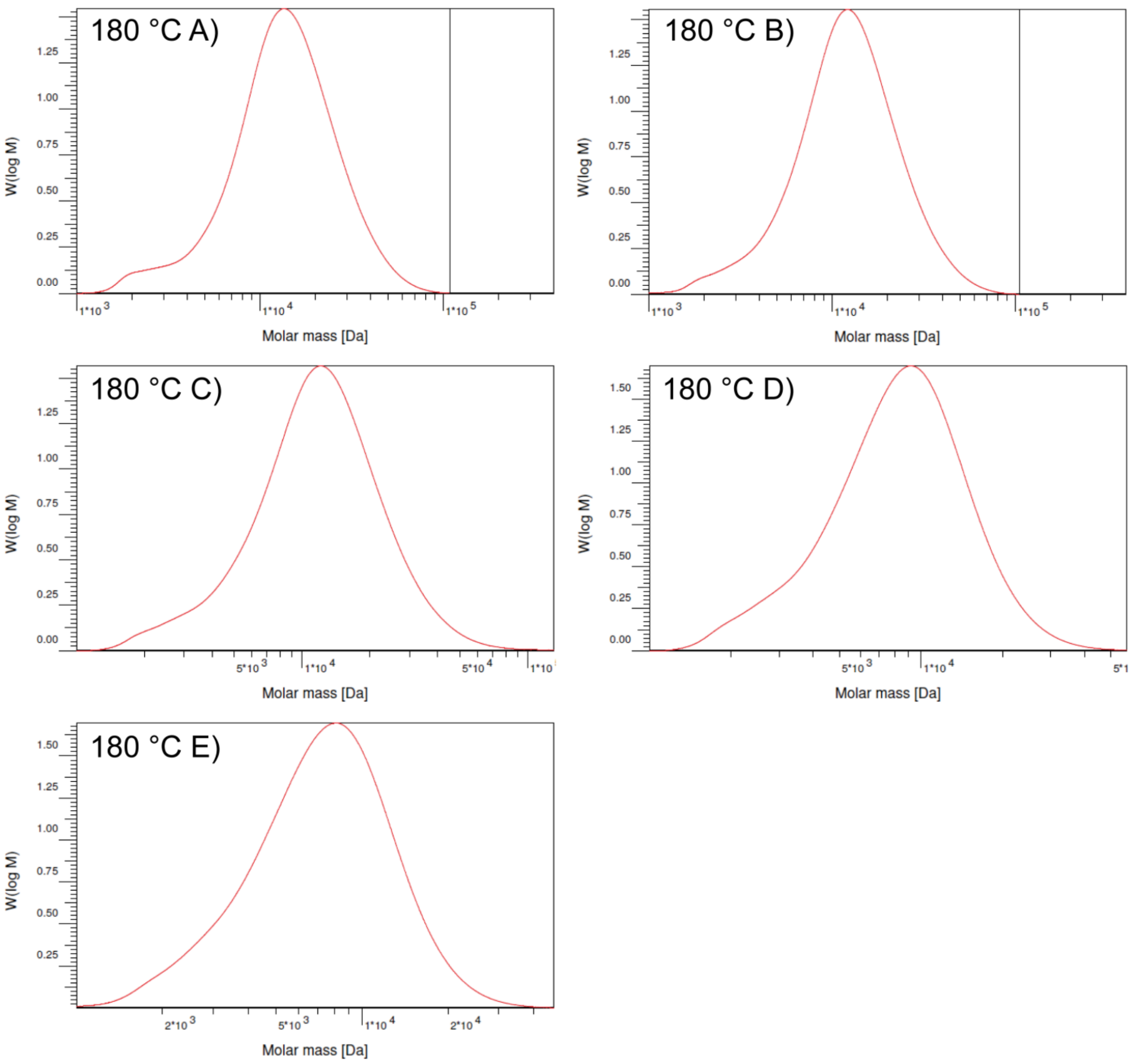

Supplementary Figure 8: Molecular weight of **poly5-3S** at different activator concentrations at 180 °C.

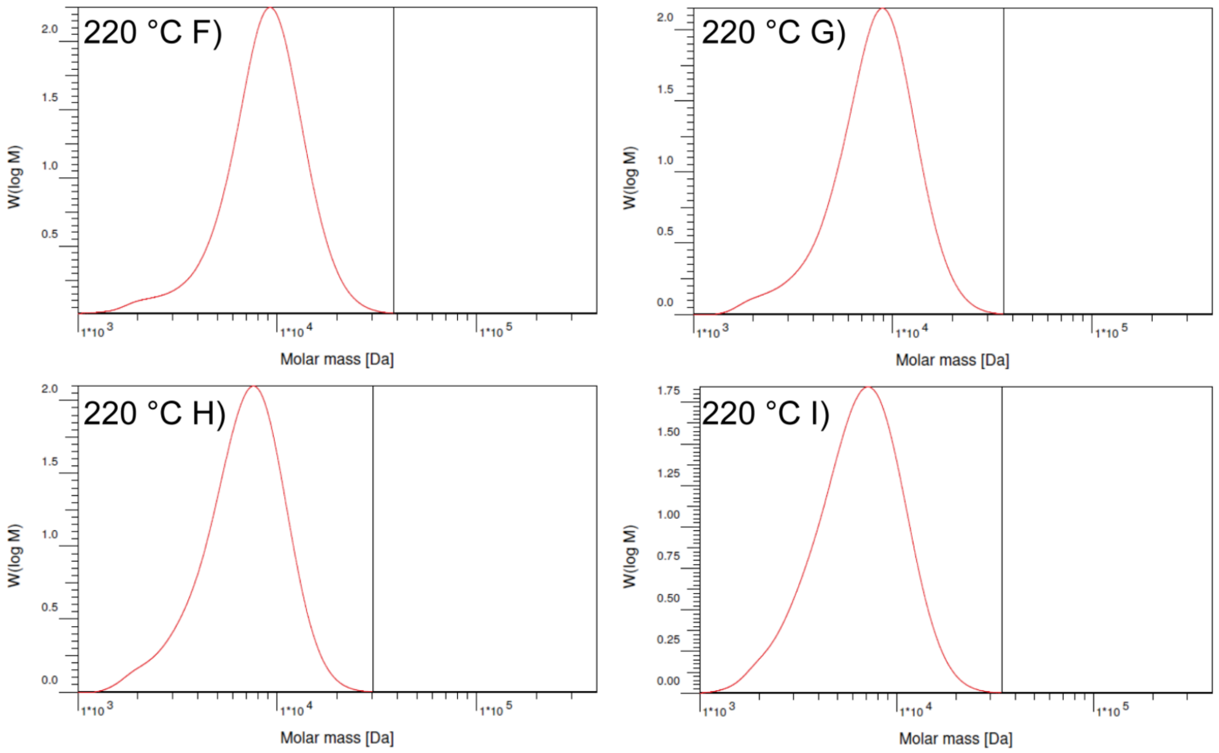

Supplementary Figure 9: Molecular weight of **poly5-3S** at different activator concentrations at 220 °C.

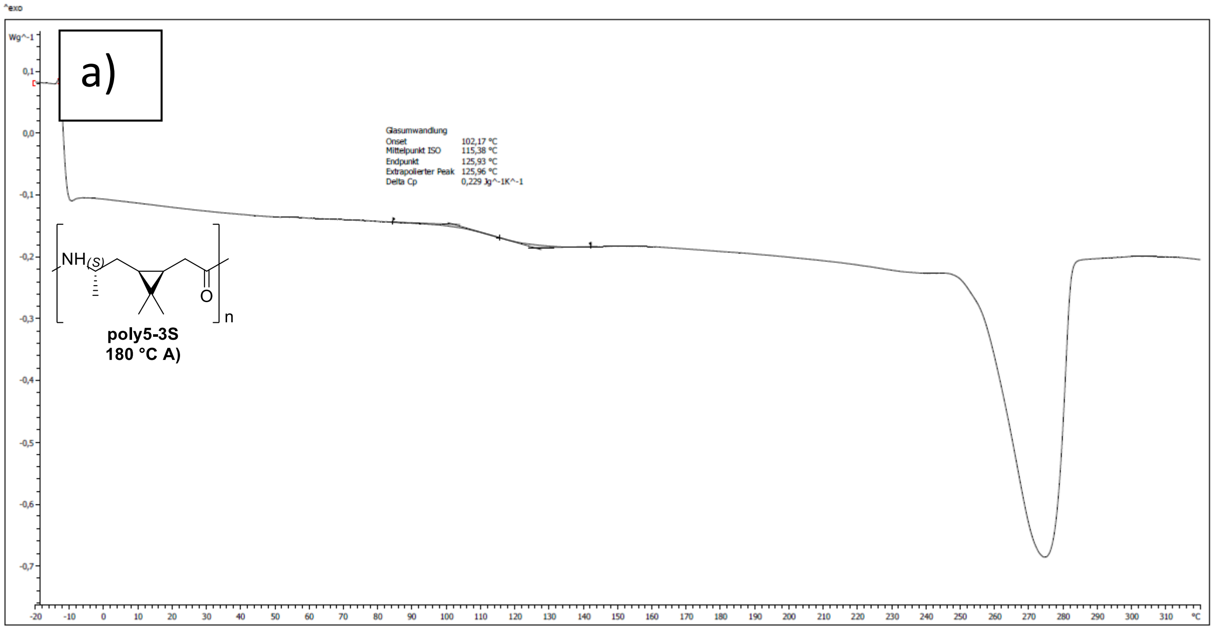

^exo
Wg^-1
a)
Glasumwandlung
Onset 102,17 °C
Mittelpunkt ISO 115,38 °C
Endpunkt 125,93 °C
Extrapolierter Peak 125,96 °C
Delta Cp 0,229 Jg^-1K^-1
NH
(S)
O
n
poly5-3S
180 °C A)
°C

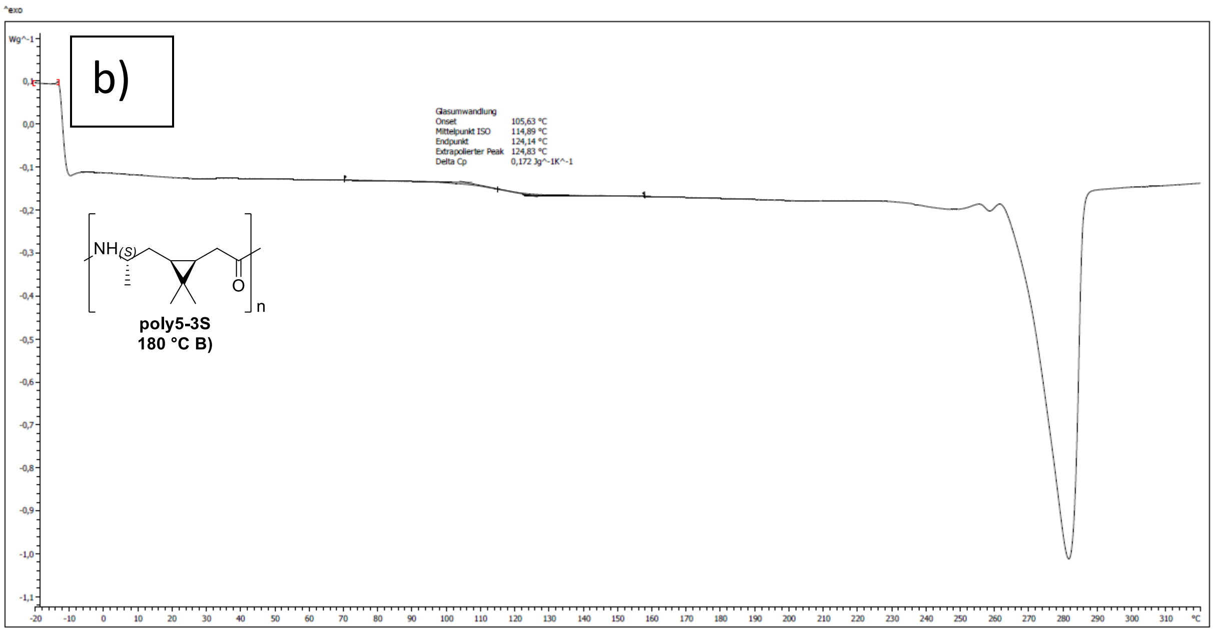

^exo
Wg^-1
b)
Glasumwandlung
Onset 105,63 °C
Mittelpunkt ISO 114,89 °C
Endpunkt 124,14 °C
Extrapolierter Peak 124,83 °C
Delta Cp 0,172 Jg^-1K^-1
NH
(S)
O
n
poly5-3S
180 °C B)
°C

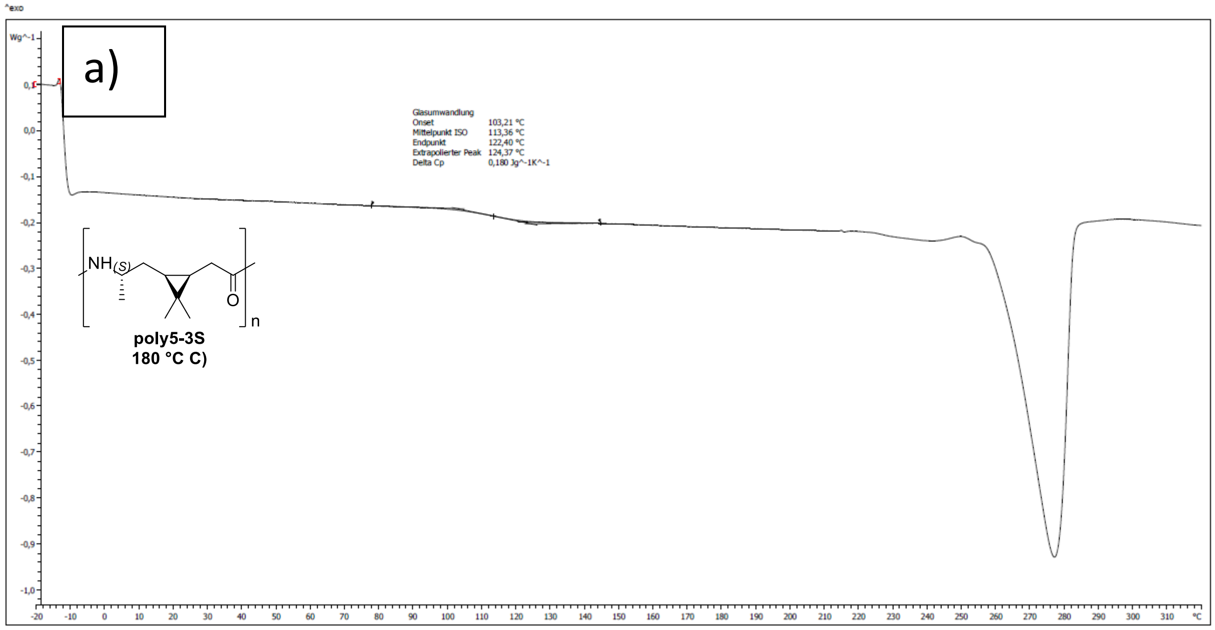
^exo
Wg^-1
a)
Glasumwandlung
Onset 103,21 °C
Mittelpunkt ISO 113,36 °C
Endpunkt 122,40 °C
Extrapolierter Peak 124,37 °C
Delta Cp 0,180 Jg^-1K^-1
NH
(S)
O
n
poly5-3S
180 °C C)
°C

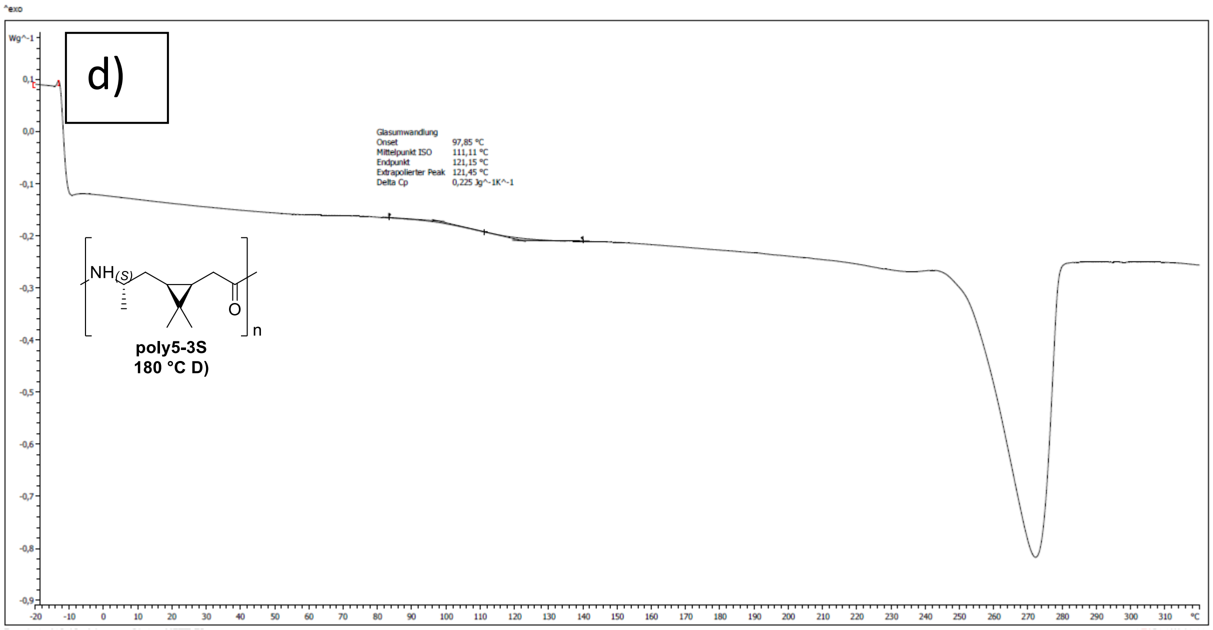
^exo
Wg^-1
d)
Glasumwandlung
Onset 97,85 °C
Mittelpunkt ISO 111,11 °C
Endpunkt 121,15 °C
Extrapolierter Peak 121,45 °C
Delta Cp 0,225 Jg^-1K^-1
NH
(S)
O
n
poly5-3S
180 °C D)
°C

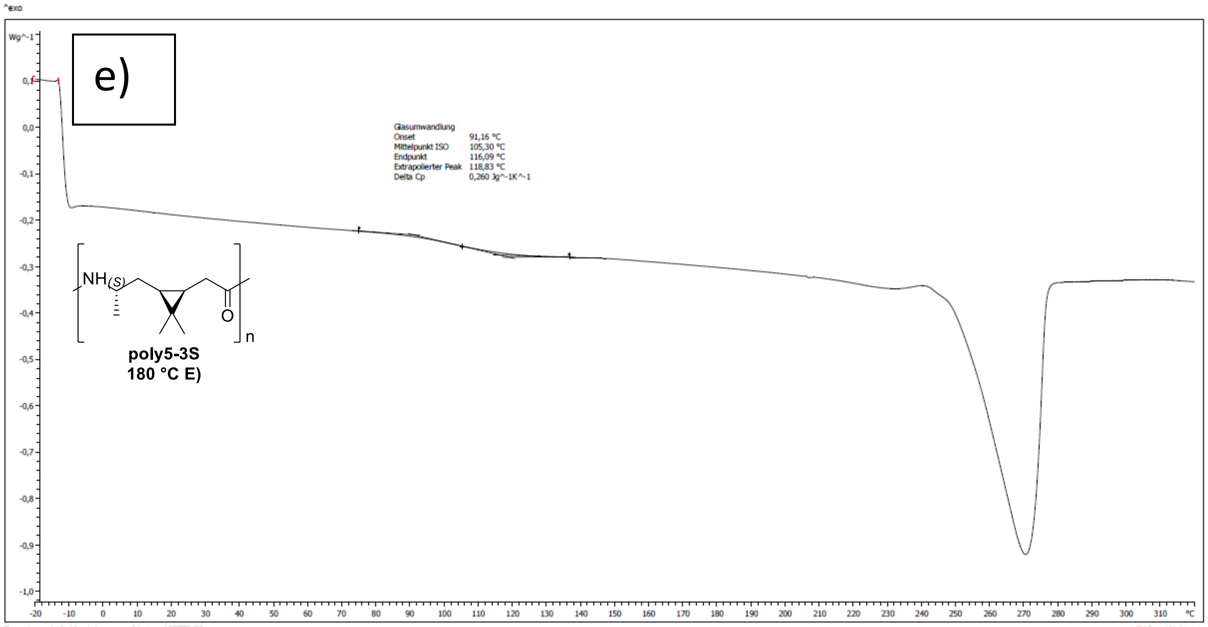
^exo
Wg^-1
e)
Glasumwandlung
Onset 91,16 °C
Mittelpunkt ISO 105,30 °C
Endpunkt 116,09 °C
Extrapolierter Peak 118,83 °C
Delta Cp 0,260 Jg^-1K^-1
NH
(S)
O
n
poly5-3S
180 °C E)
°C

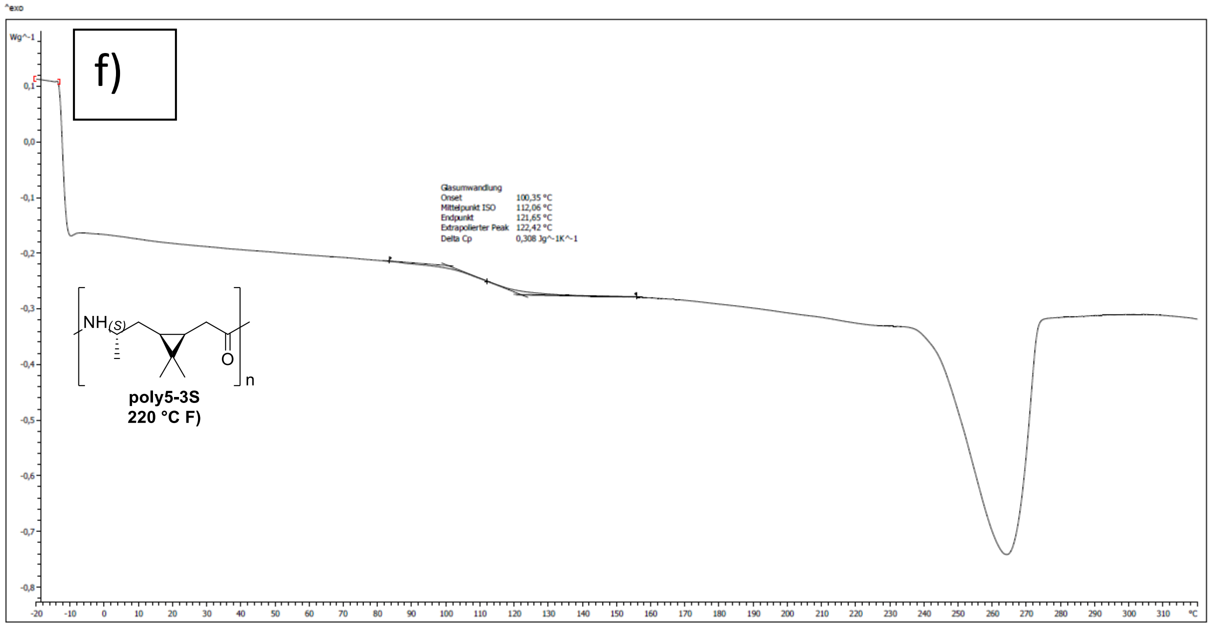
^exo
Wg^-1
f)
Glasumwandlung
Onset 100,35 °C
Mittelpunkt ISO 112,06 °C
Endpunkt 121,65 °C
Extrapolierter Peak 122,42 °C
Delta Cp 0,308 Jg^-1K^-1
NH
(S)
O
n
poly5-3S
220 °C F)
°C

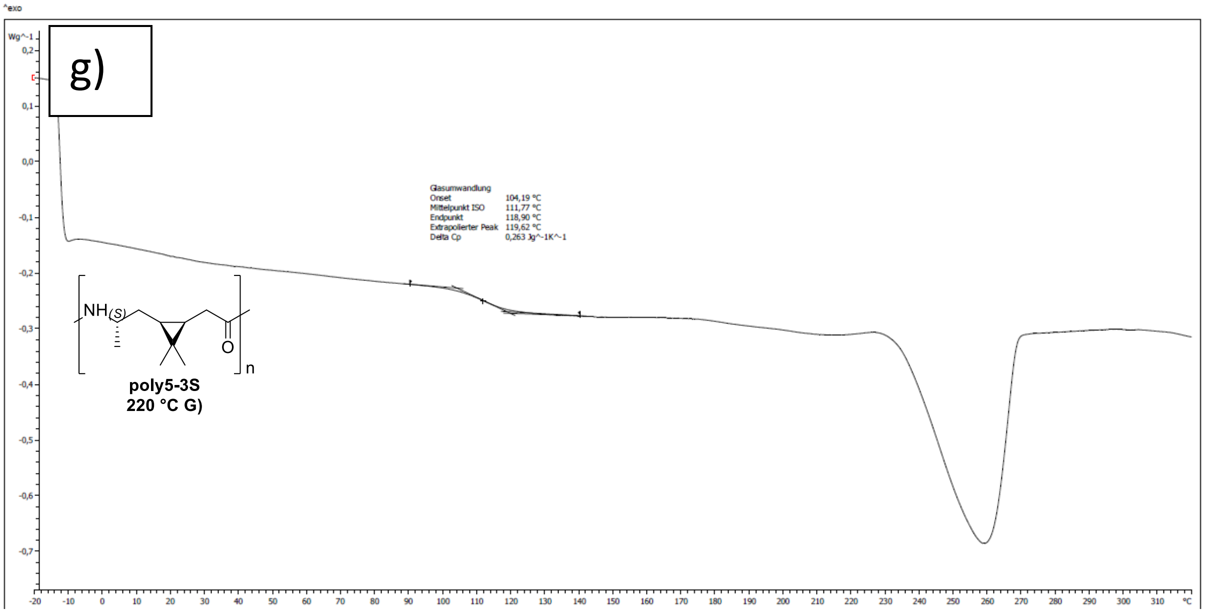

^exo
Wg^-1
g)
Glasumwandlung
Onset 104,19 °C
Mittelpunkt ISO 111,77 °C
Endpunkt 118,90 °C
Extrapolierter Peak 119,62 °C
Delta Cp 0,263 Jg^-1K^-1
NH
(S)
O
n
poly5-3S
220 °C G)
°C

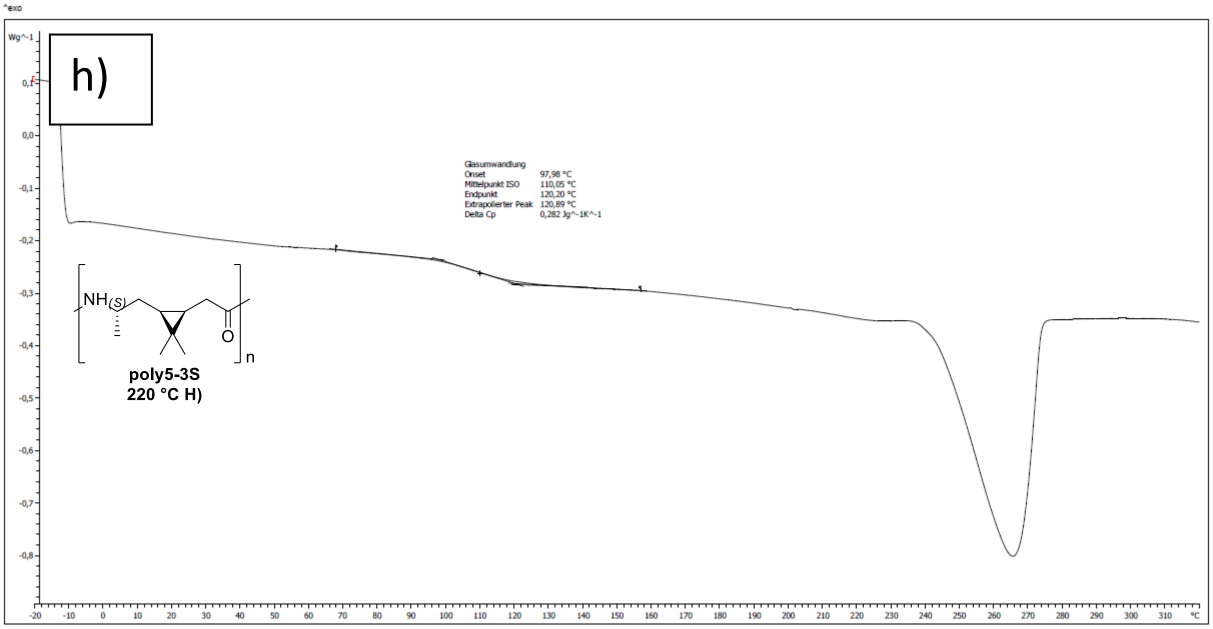

^exo
Wg^-1
h)
Glasumwandlung
Onset 97,98 °C
Mittelpunkt ISO 110,05 °C
Endpunkt 120,20 °C
Extrapolierter Peak 120,89 °C
Delta Cp 0,282 Jg^-1K^-1
NH
(S)
O
n
poly5-3S
220 °C H)
°C

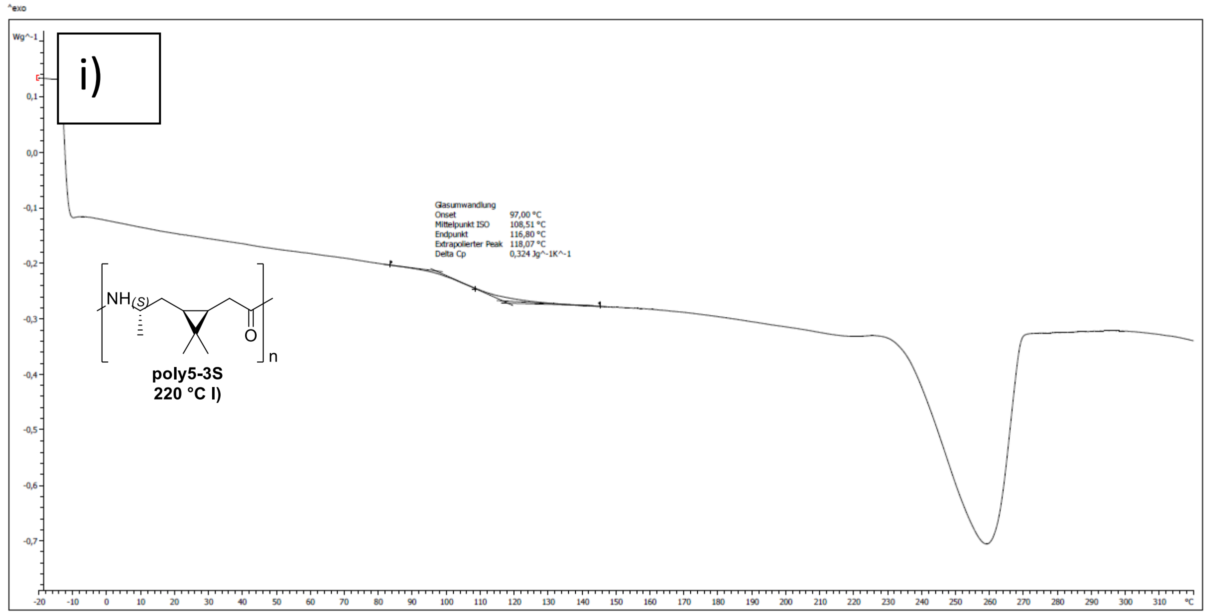

Supplementary Figure 10: DSC curves of various poly-3S-caranamides a)-i) as described in Supplementary Table 12, measured using DSC method B, segment 10 (heating, black).

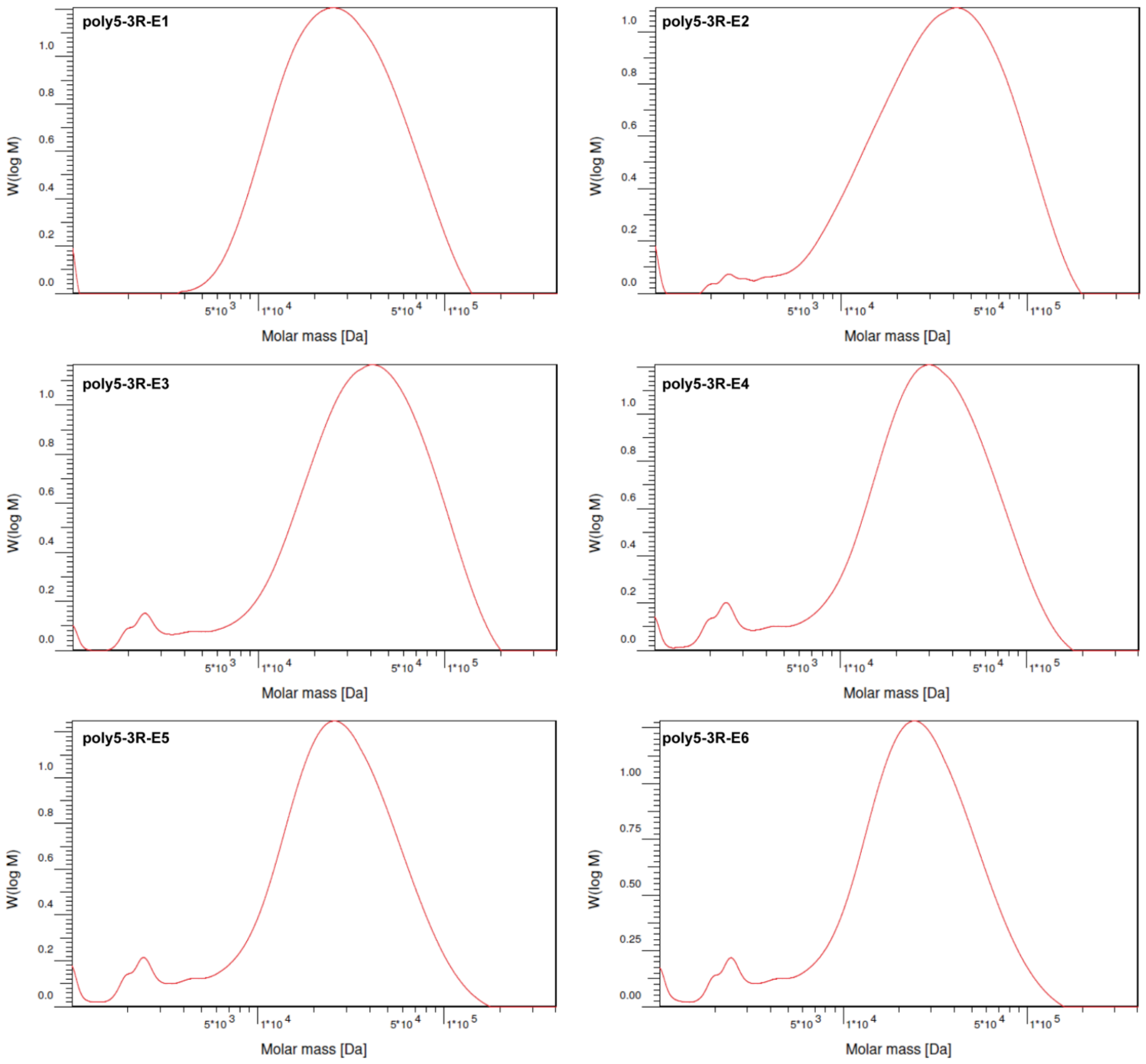

Supplementary Figure 11: Molecular weight of **poly5-3R** at different activator concentrations.

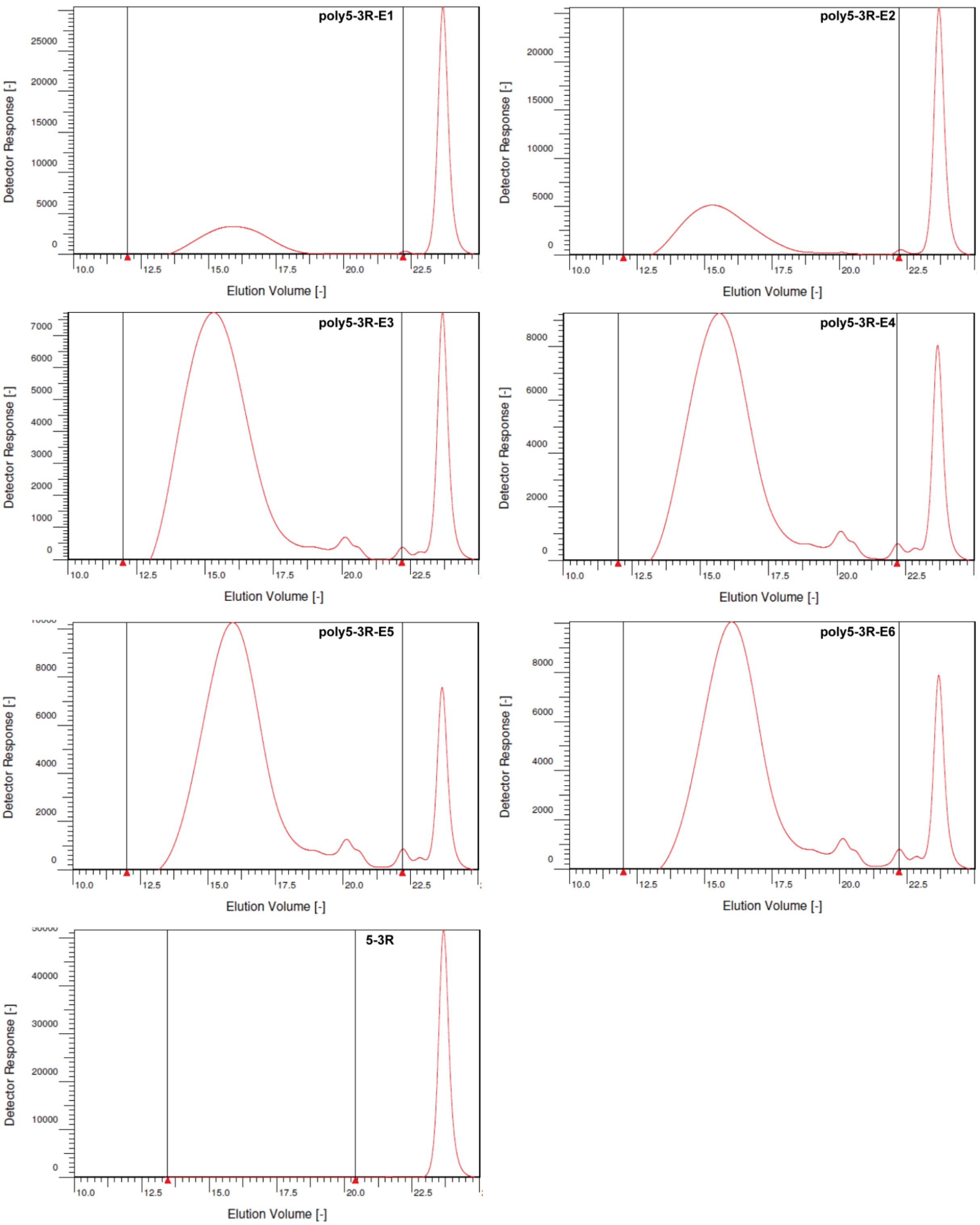

Supplementary Figure 12: GPC elugrams of **poly5R** displaying the monomer conversion at different activator concentrations

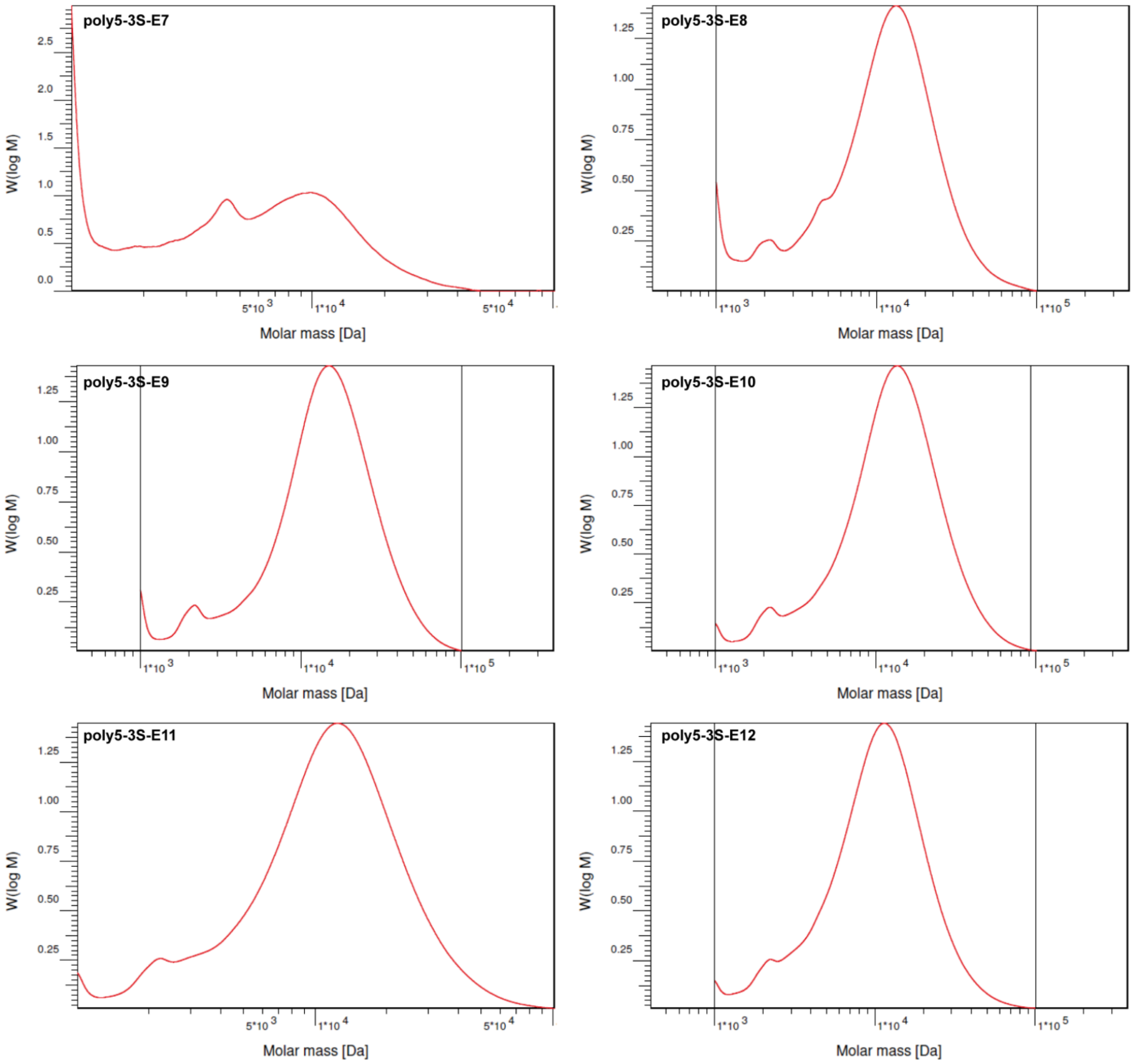

Supplementary Figure 13: Molecular weight of **poly5-3S** at different activator concentrations.

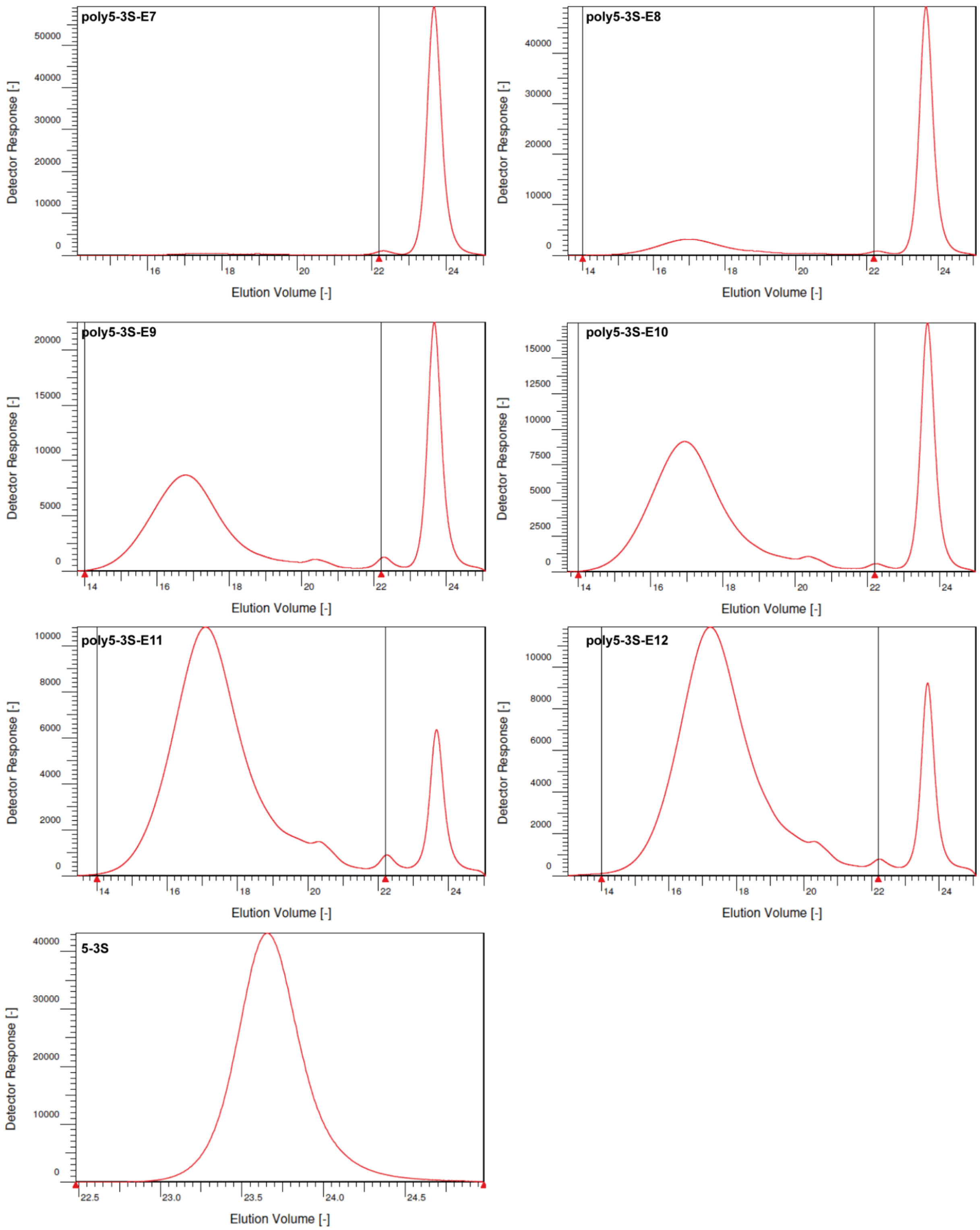

Supplementary Figure 14: GPC elugrams of **poly5R** displaying the monomer conversion at different activator concentrations.

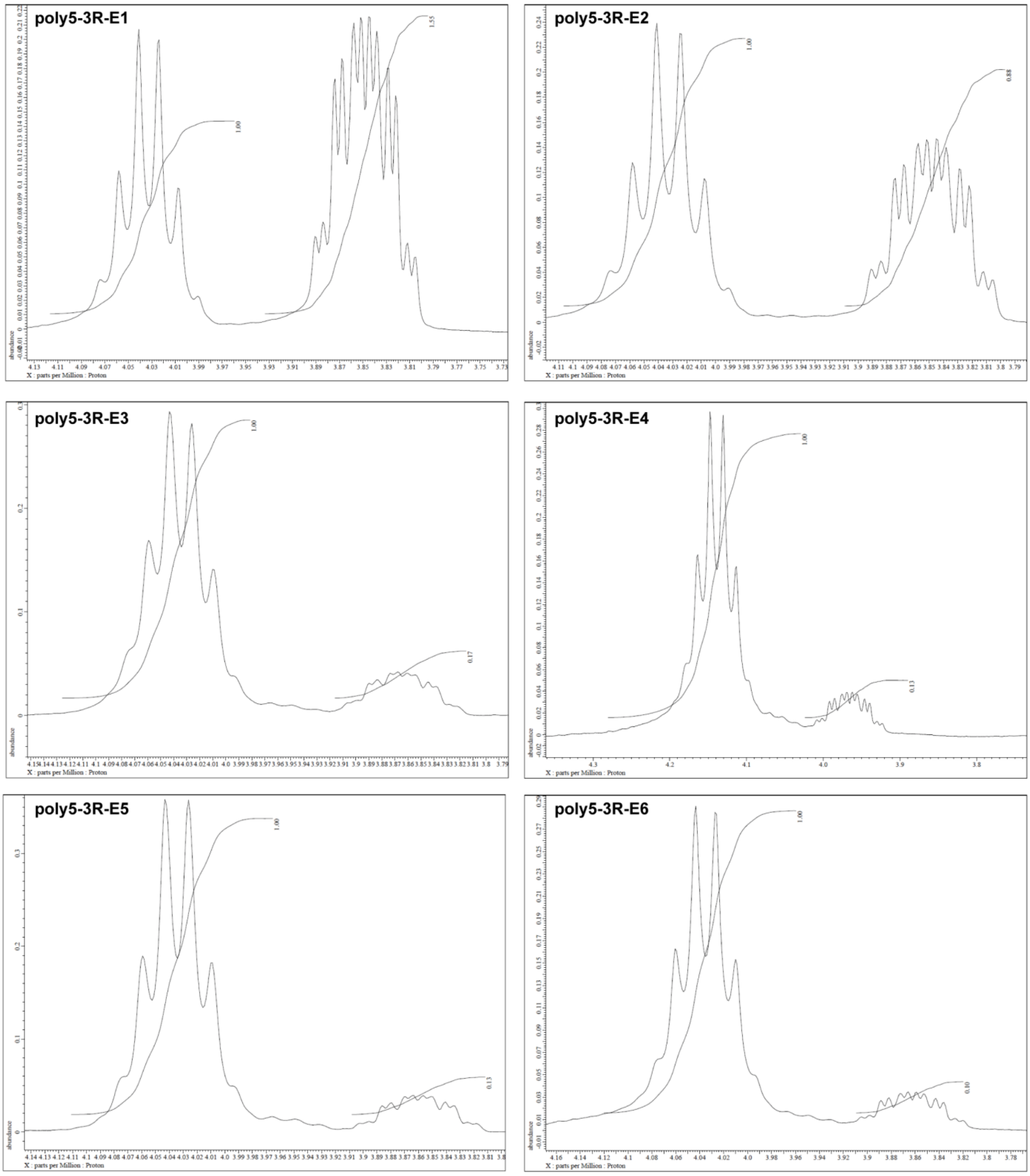

Supplementary Figure 15: Evaluation of the conversion of monomer **5-3R** at different concentrations of activator.

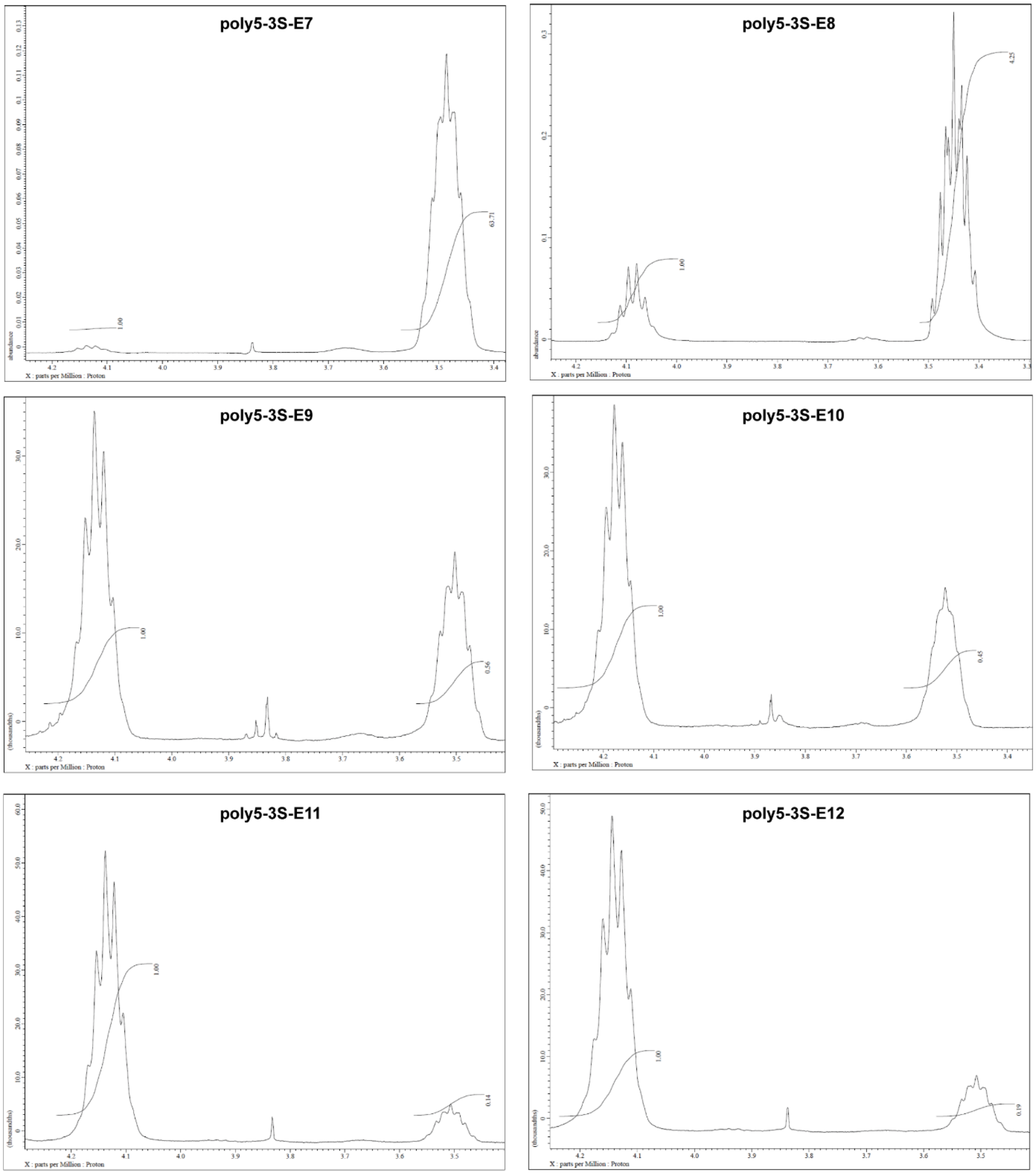

Supplementary Figure 16: Evaluation of the conversion of monomer **5-3S** at different concentrations of activator.

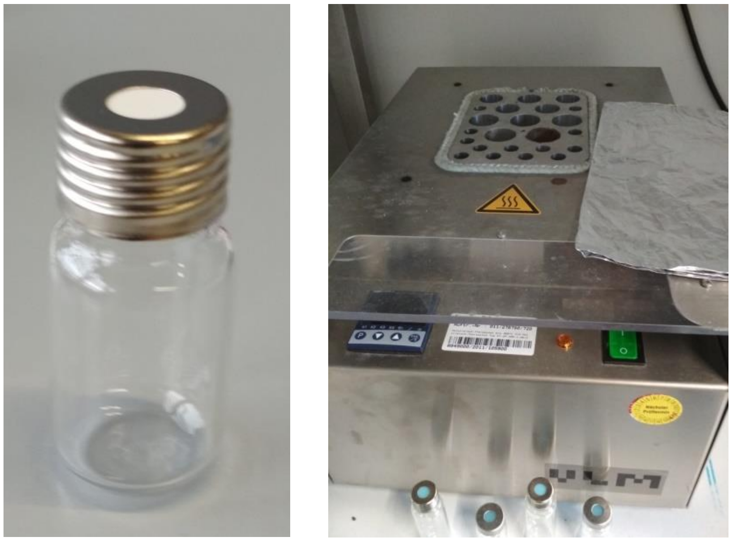

Supplementary Figure 17: Pictures of the reaction vial and oven for polymerization method B.

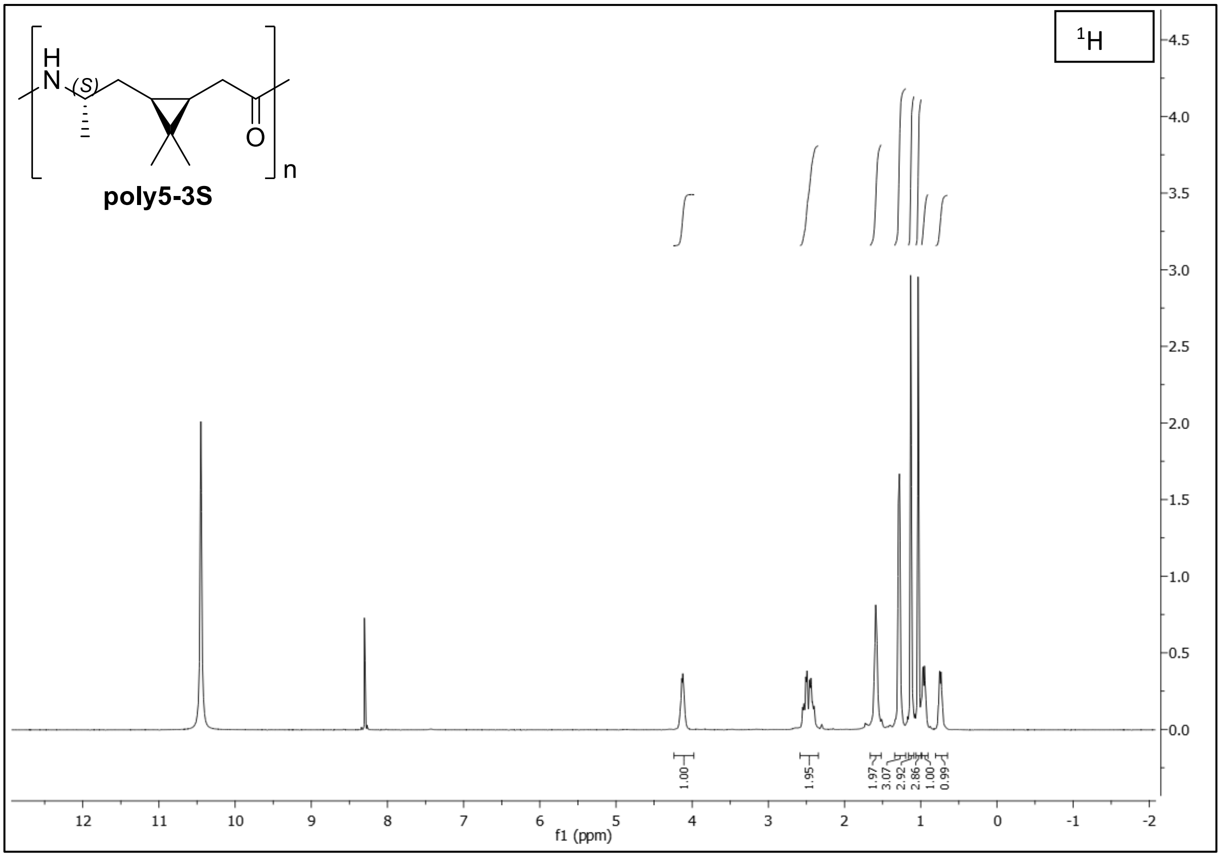
poly5-3S
1H
1.00
1.95
1.97
3.07
2.92
2.86
1.00
0.99
f1 (ppm)

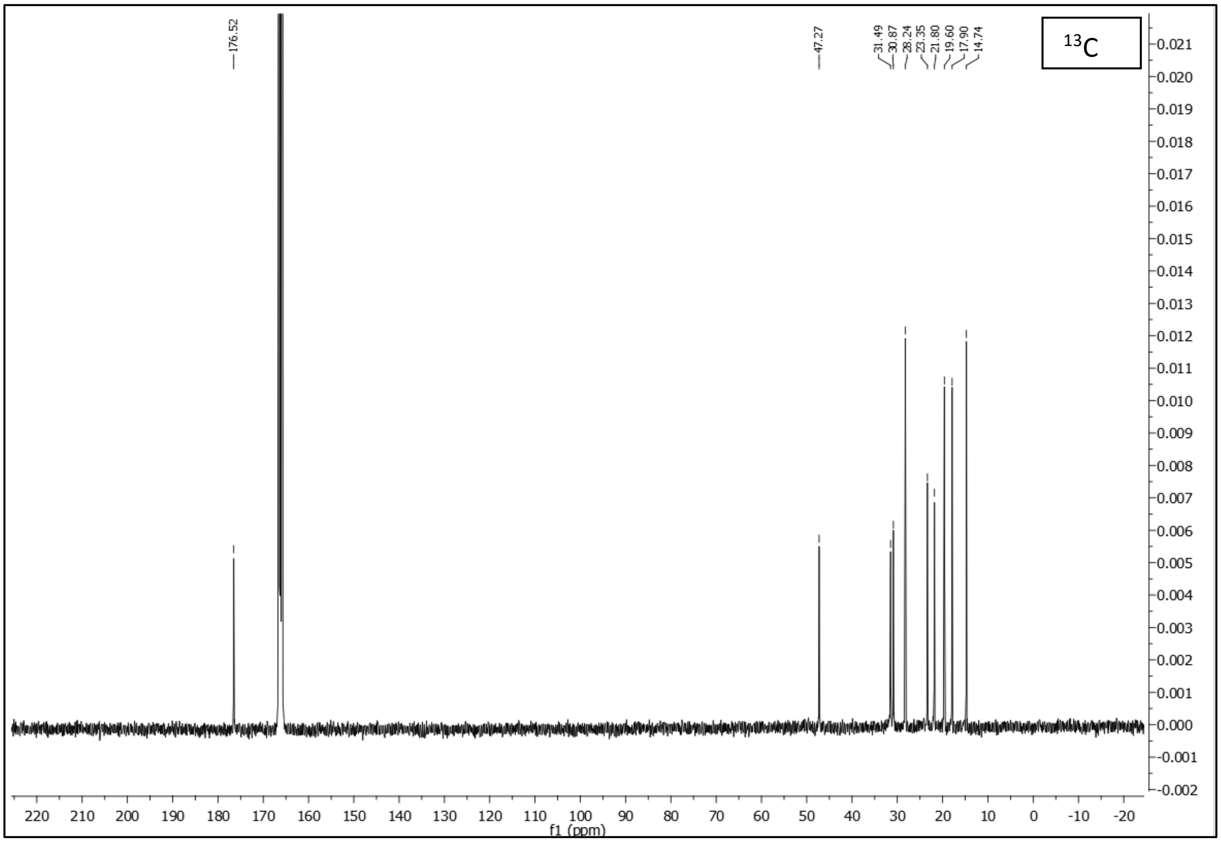
13C
176.52
47.27
31.49
30.87
28.24
23.35
21.80
19.60
17.90
14.74
f1 (ppm)

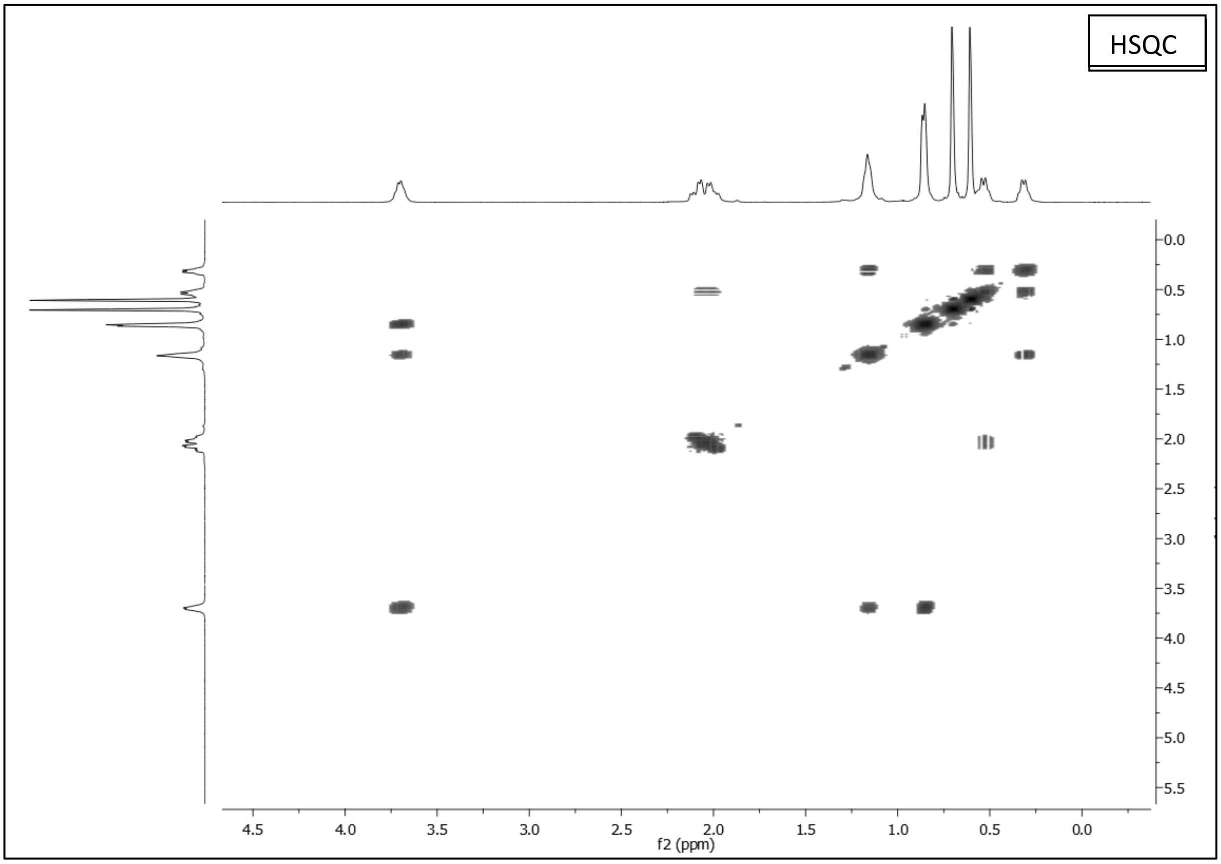
HSQC
f2 (ppm)

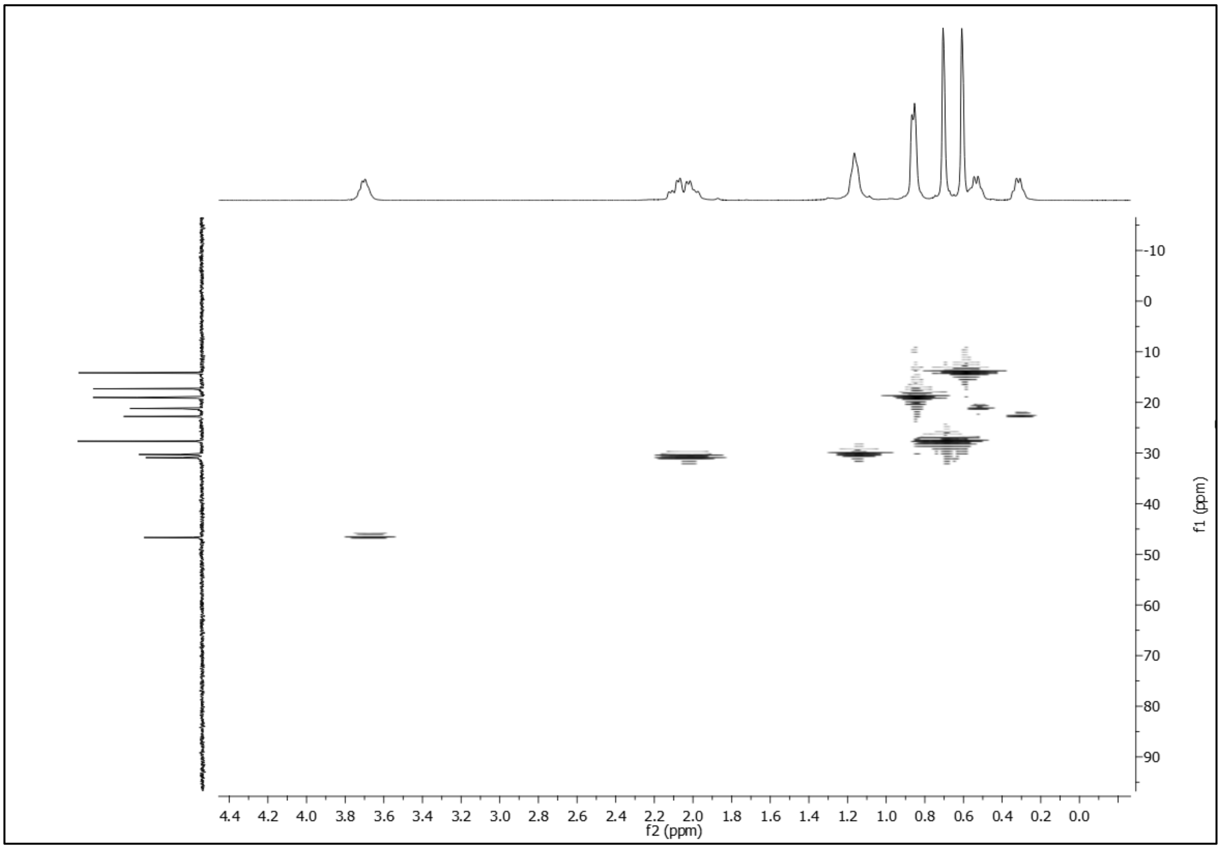
f2 (ppm)
f1 (ppm)

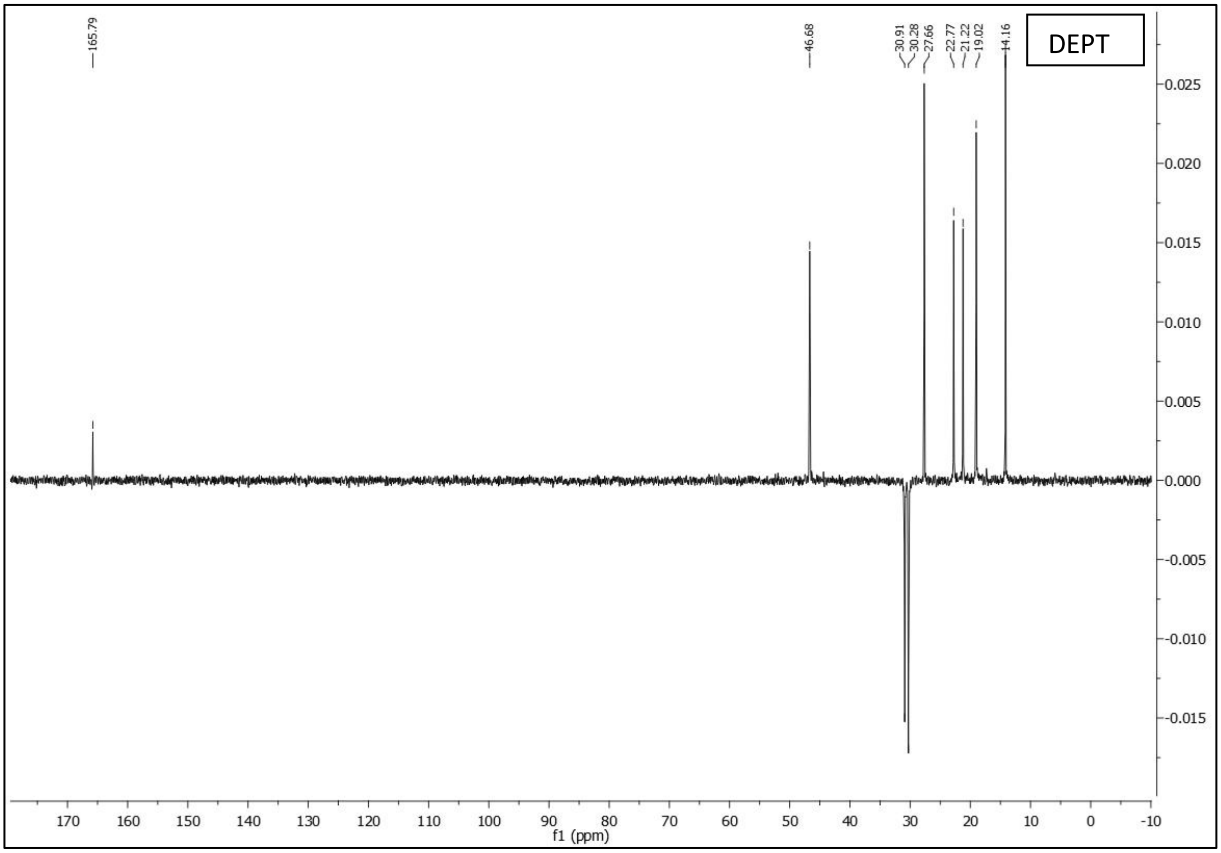

Supplementary Figure 18: NMR-spectra of Poly-3S-caranamide (DCOOD, 1H 400 MHz, 13C 100 MHz).

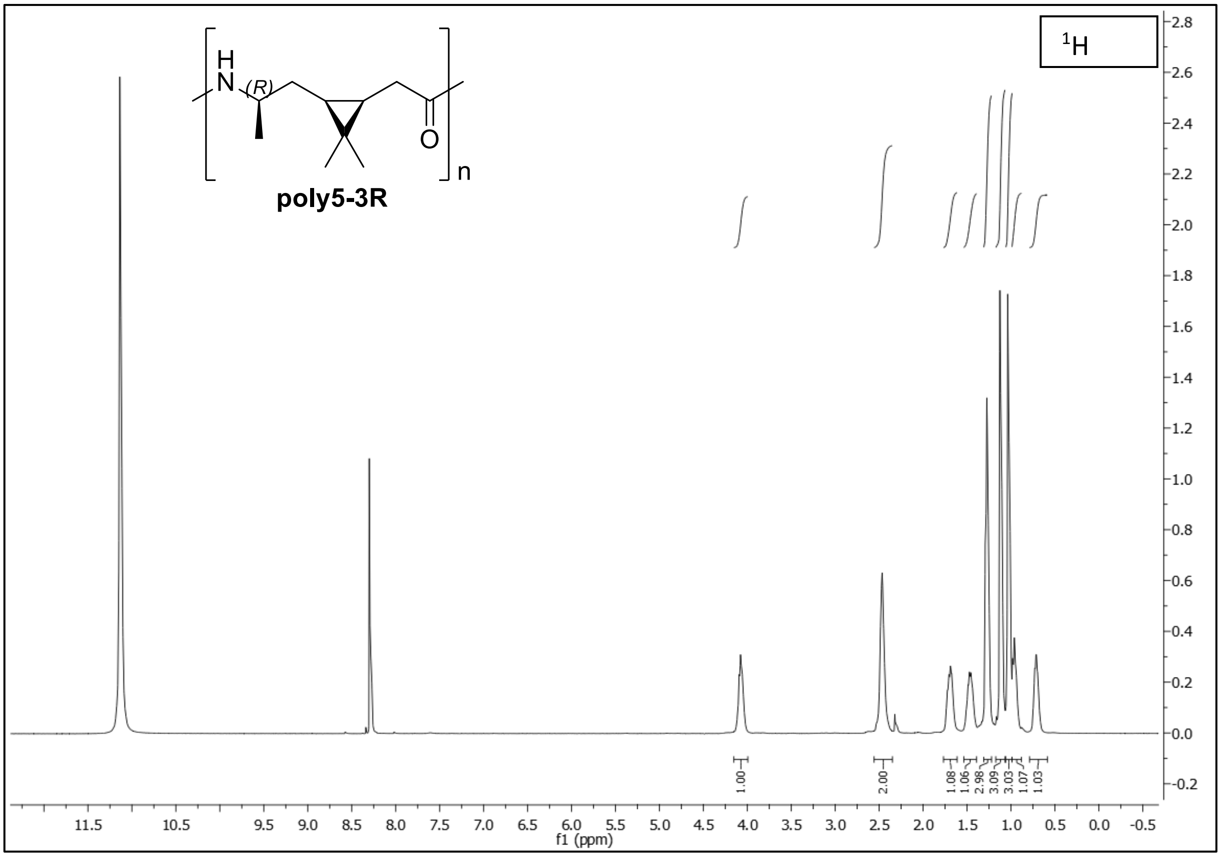

poly5-3R
(R)
n
1H
1.00
2.00
1.08
1.06
2.98
3.09
3.03
1.07
1.03
f1 (ppm)

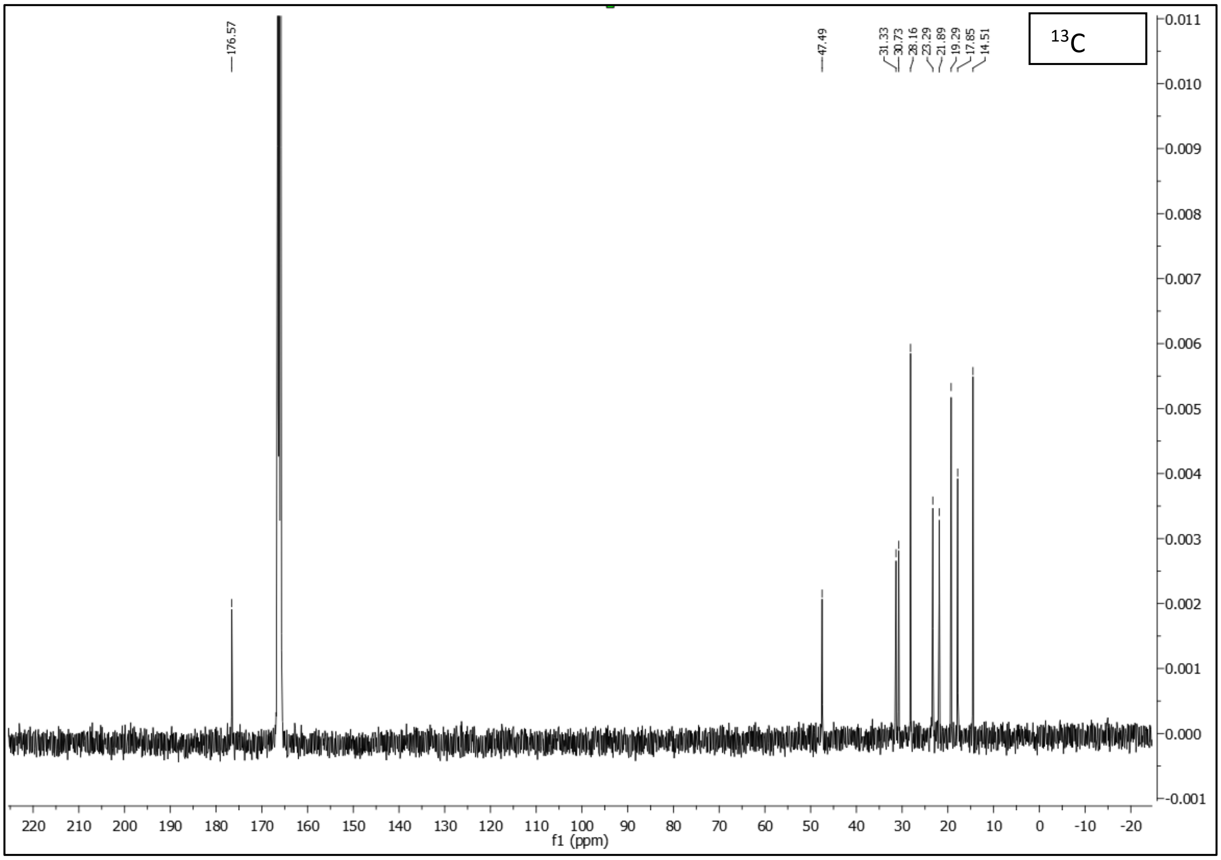

13C
176.57
47.49
31.33
30.73
28.16
23.29
21.89
19.29
17.85
14.51
f1 (ppm)

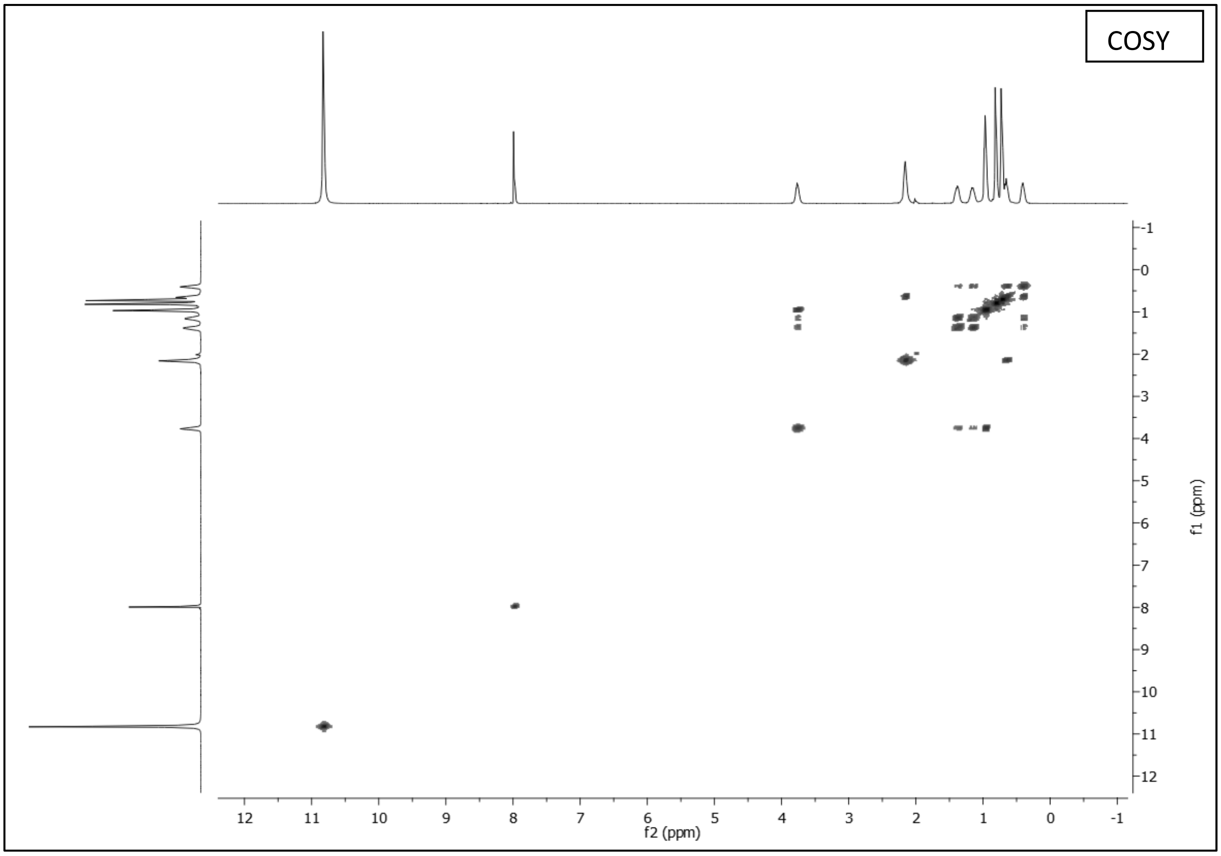
COSY
f2 (ppm)
f1 (ppm)

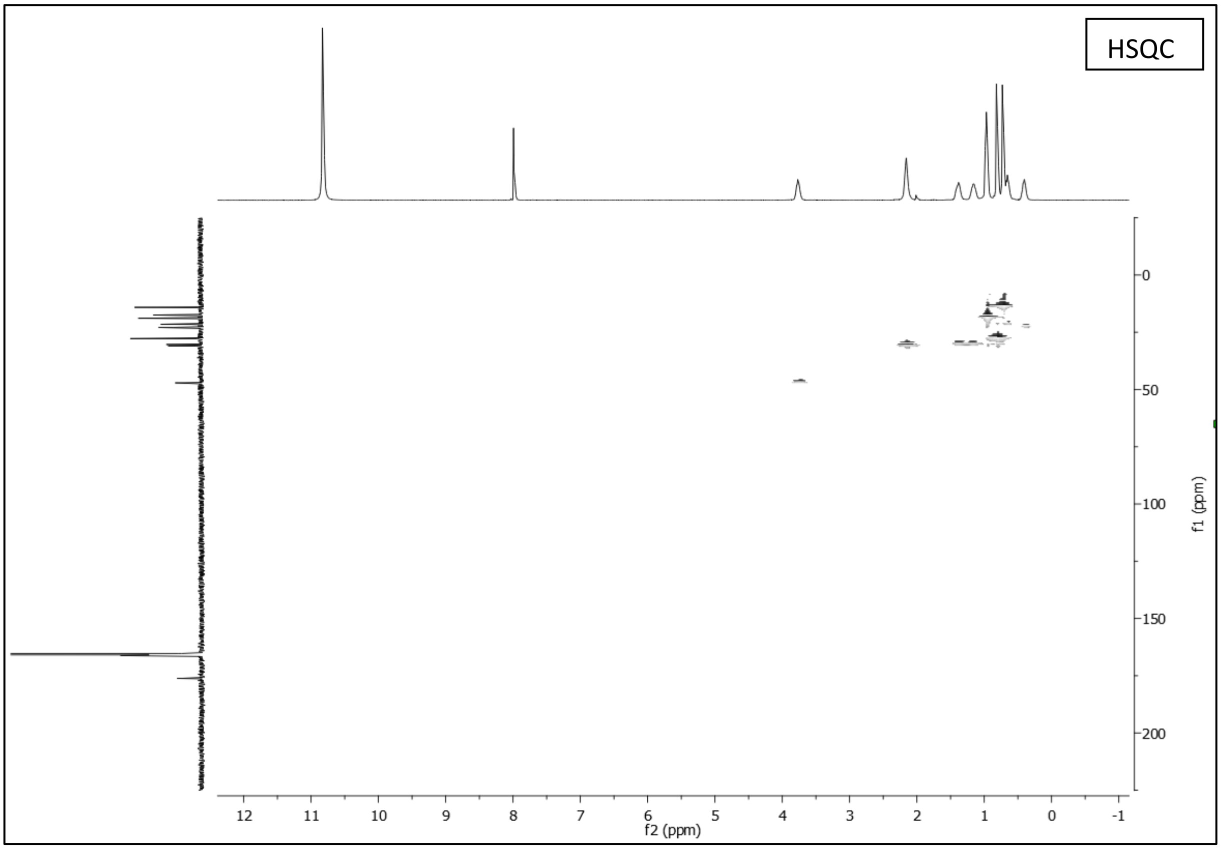
HSQC
f2 (ppm)
f1 (ppm)

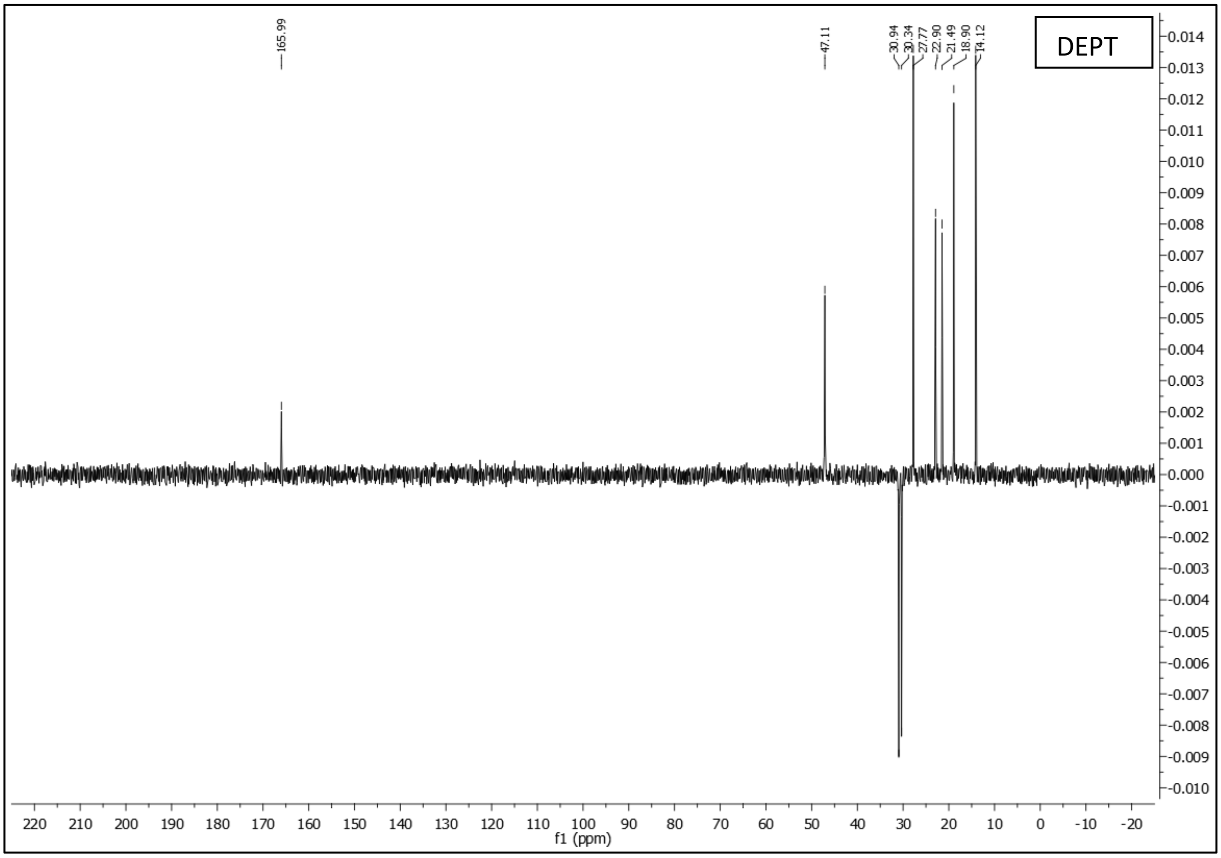

Supplementary Figure 19: NMR-spectra of Poly-3R-caranamide (DCOOD, 1H 400 MHz, 13C 100 MHz).[12]

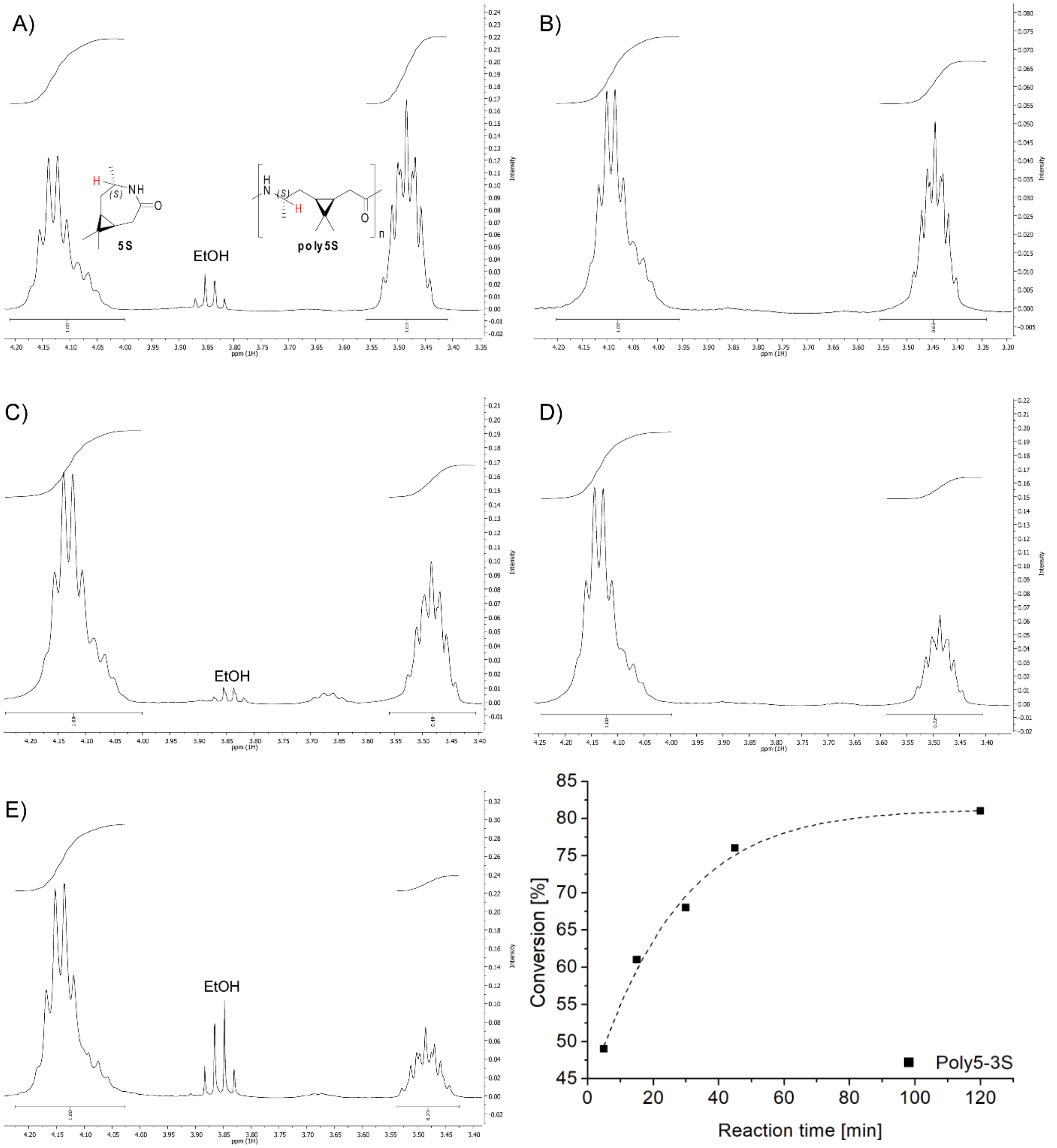

Supplementary Figure 20: Influence of the reaction time on the conversion.

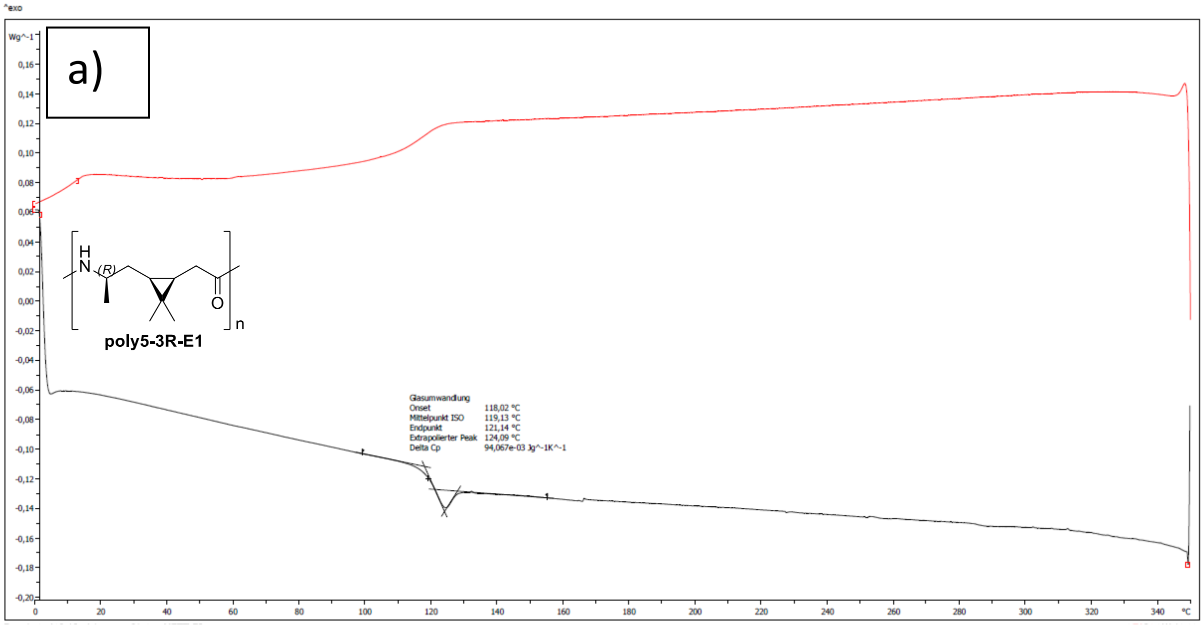
a)
^exo
Wg^-1
poly5-3R-E1
Glasumwandlung
Onset 118,02 °C
Mittelpunkt ISO 119,13 °C
Endpunkt 121,14 °C
Extrapolierter Peak 124,09 °C
Delta Cp 94,067e-03 Jg^-1K^-1
°C

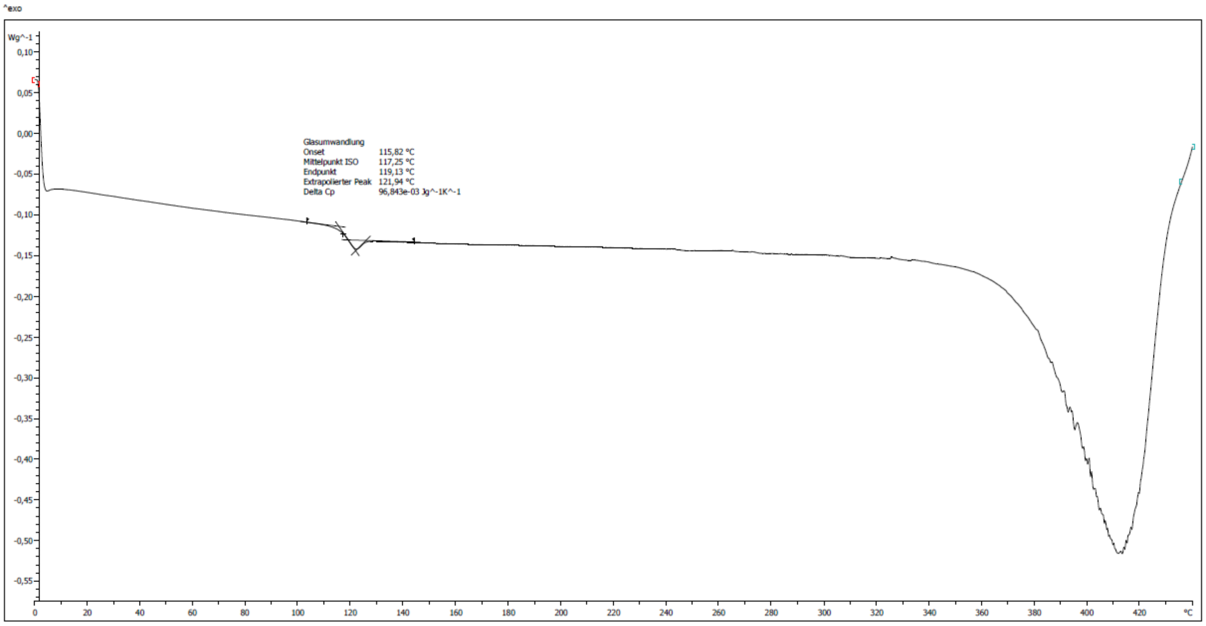
^exo
Wg^-1
Glasumwandlung
Onset 115,82 °C
Mittelpunkt ISO 117,25 °C
Endpunkt 119,13 °C
Extrapolierter Peak 121,94 °C
Delta Cp 96,843e-03 Jg^-1K^-1
°C

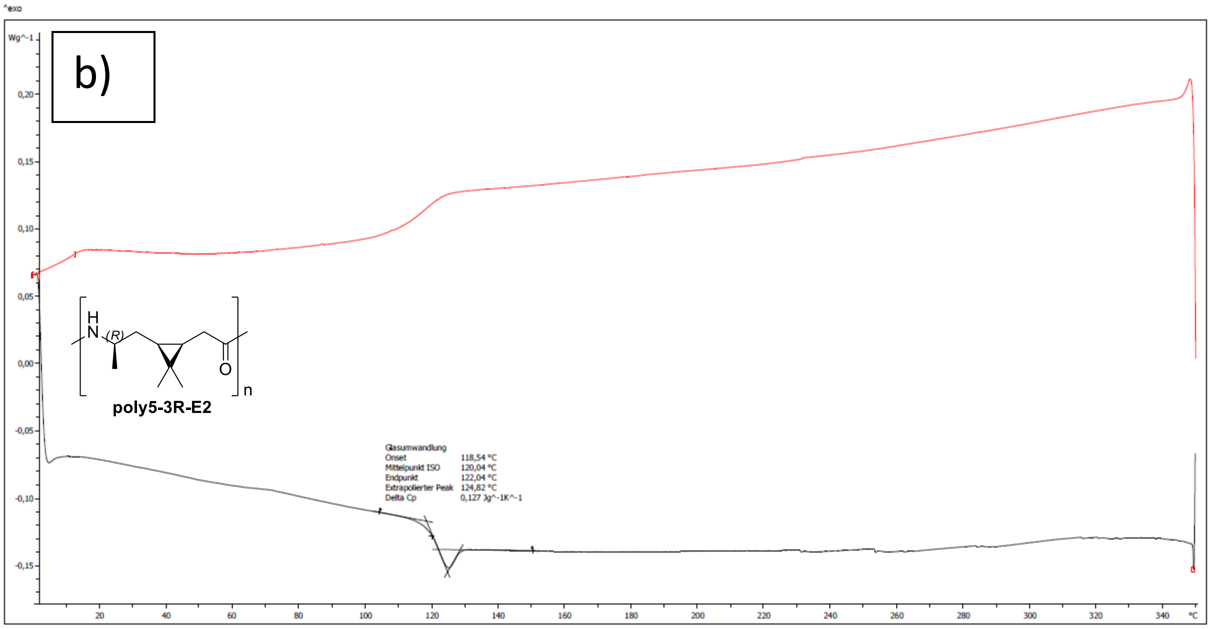
b)
^exo
Wg^-1
0,20
0,15
0,10
0,05
0,00
-0,05
-0,10
-0,15
20
40
60
80
100
120
140
160
180
200
220
240
260
280
300
320
340
°C
H
N
(R)
O
n
poly5-3R-E2
Glasumwandlung
Onset 118,54 °C
Mittelpunkt ISO 120,04 °C
Endpunkt 122,04 °C
Extrapolierter Peak 124,82 °C
Delta Cp 0,127 Jg^-1K^-1

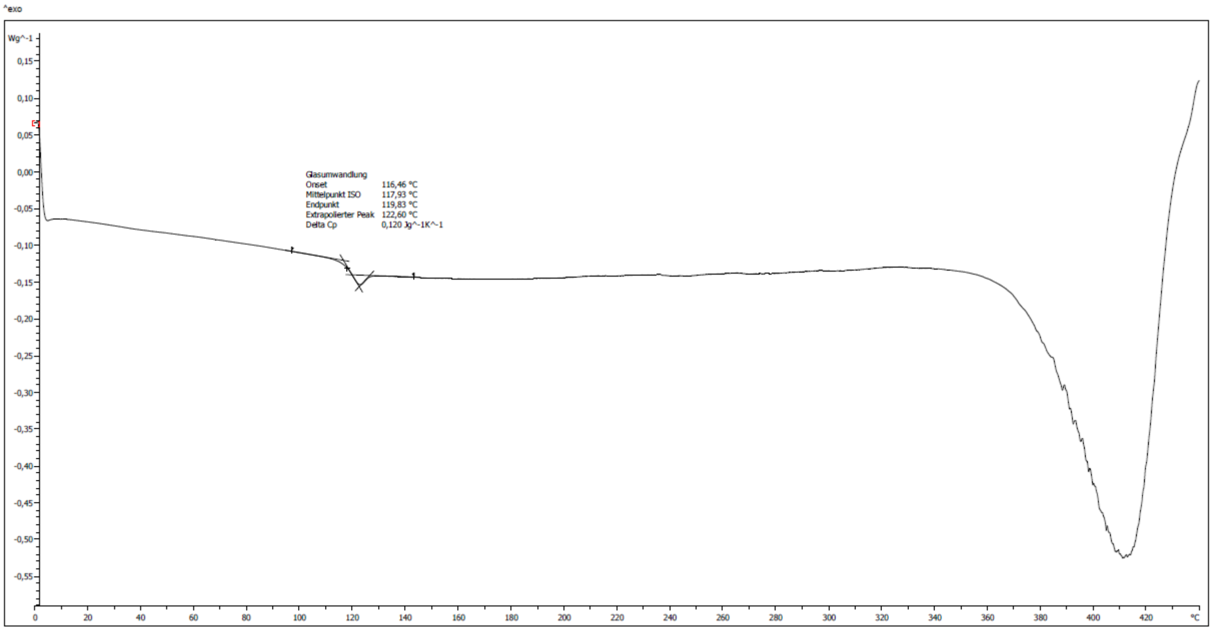
^exo
Wg^-1
0,15
0,10
0,05
0,00
-0,05
-0,10
-0,15
-0,20
-0,25
-0,30
-0,35
-0,40
-0,45
-0,50
-0,55
0
20
40
60
80
100
120
140
160
180
200
220
240
260
280
300
320
340
360
380
400
420
°C
Glasumwandlung
Onset 116,46 °C
Mittelpunkt ISO 117,93 °C
Endpunkt 119,83 °C
Extrapolierter Peak 122,60 °C
Delta Cp 0,120 Jg^-1K^-1

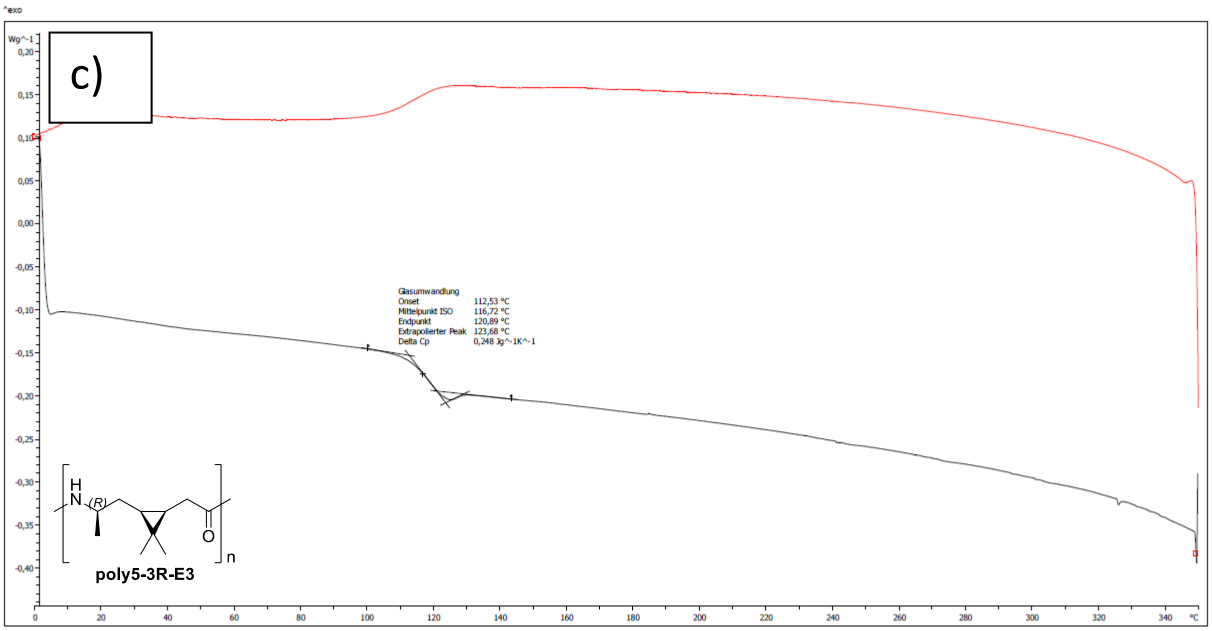
^exo
Wg^-1
c)
Glasumwandlung
Onset 112,53 °C
Mittelpunkt ISO 116,72 °C
Endpunkt 120,89 °C
Extrapolierter Peak 123,68 °C
Delta Cp 0,248 Jg^-1K^-1
poly5-3R-E3
°C

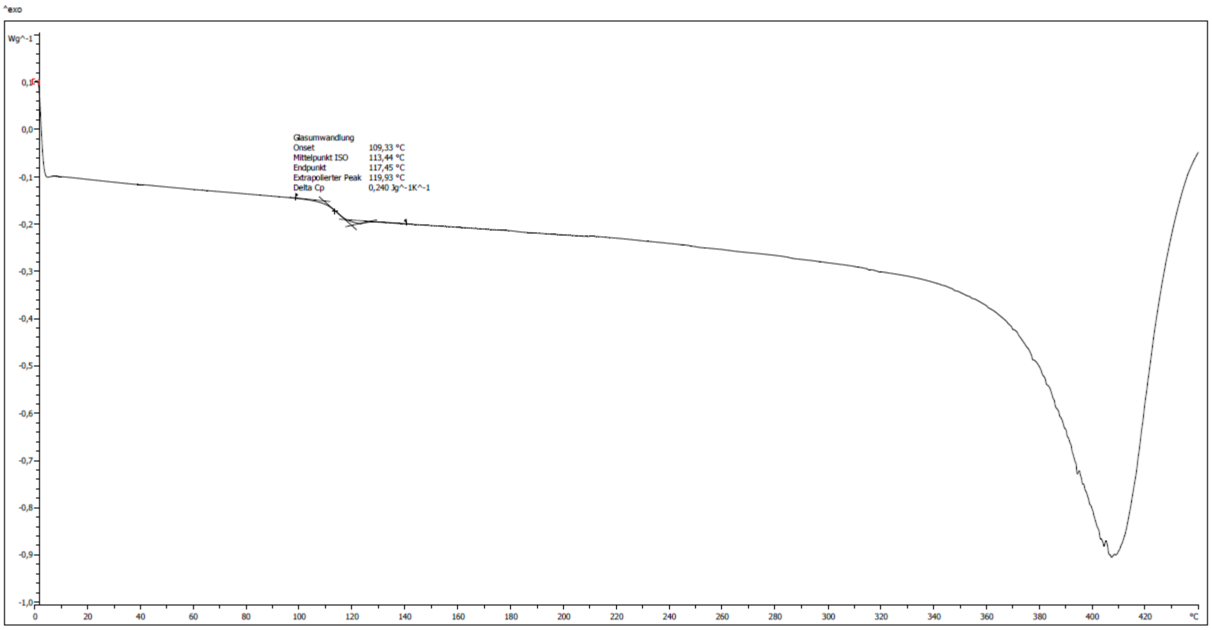
^exo
Wg^-1
Glasumwandlung
Onset 109,33 °C
Mittelpunkt ISO 113,44 °C
Endpunkt 117,45 °C
Extrapolierter Peak 119,93 °C
Delta Cp 0,240 Jg^-1K^-1
°C

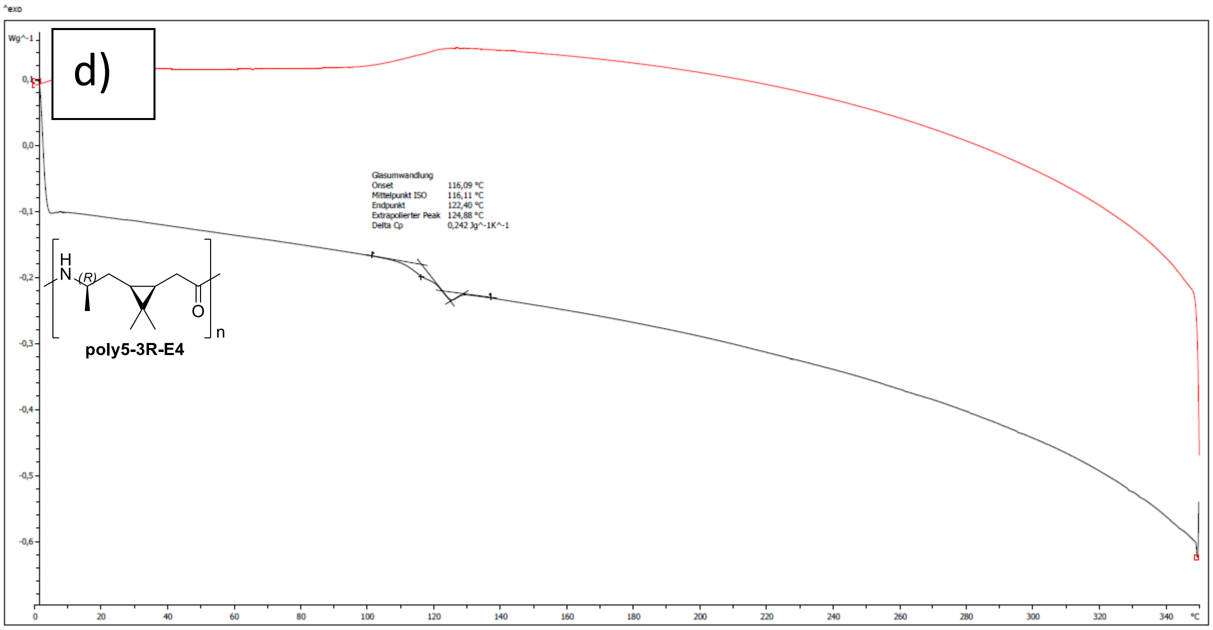

^exo
Wg^-1
d)
Glasumwandlung
Onset 116,09 °C
Mittelpunkt ISO 116,11 °C
Endpunkt 122,40 °C
Extrapolierter Peak 124,88 °C
Delta Cp 0,242 Jg^-1K^-1
poly5-3R-E4
°C

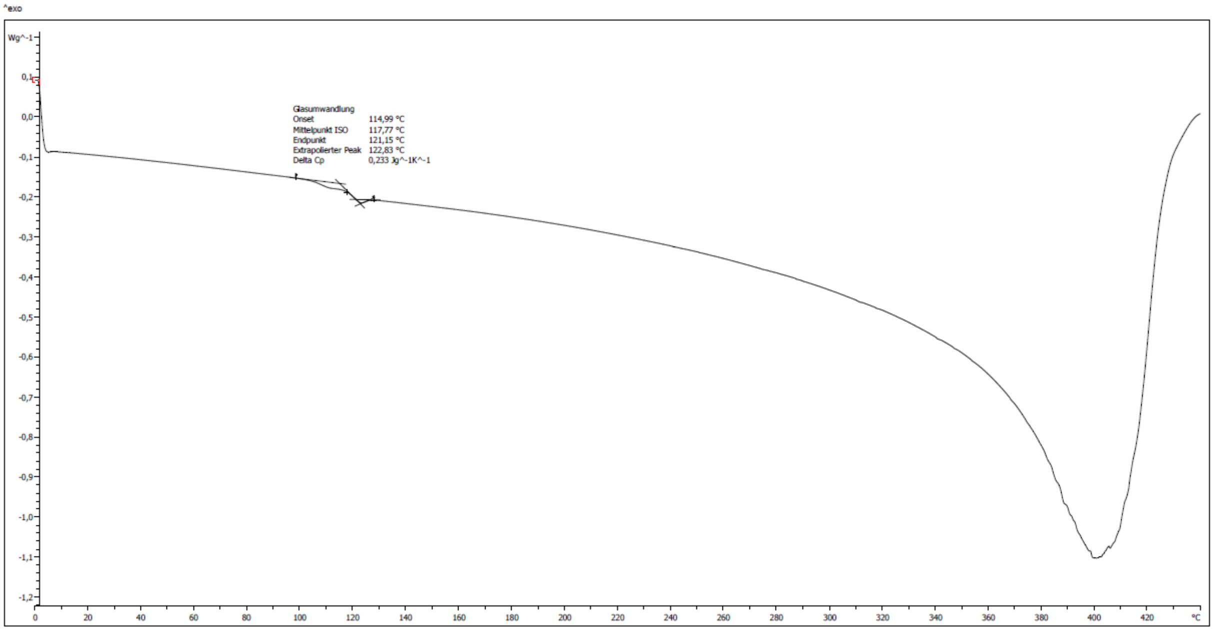

^exo
Wg^-1
Glasumwandlung
Onset 114,99 °C
Mittelpunkt ISO 117,77 °C
Endpunkt 121,15 °C
Extrapolierter Peak 122,83 °C
Delta Cp 0,233 Jg^-1K^-1
°C

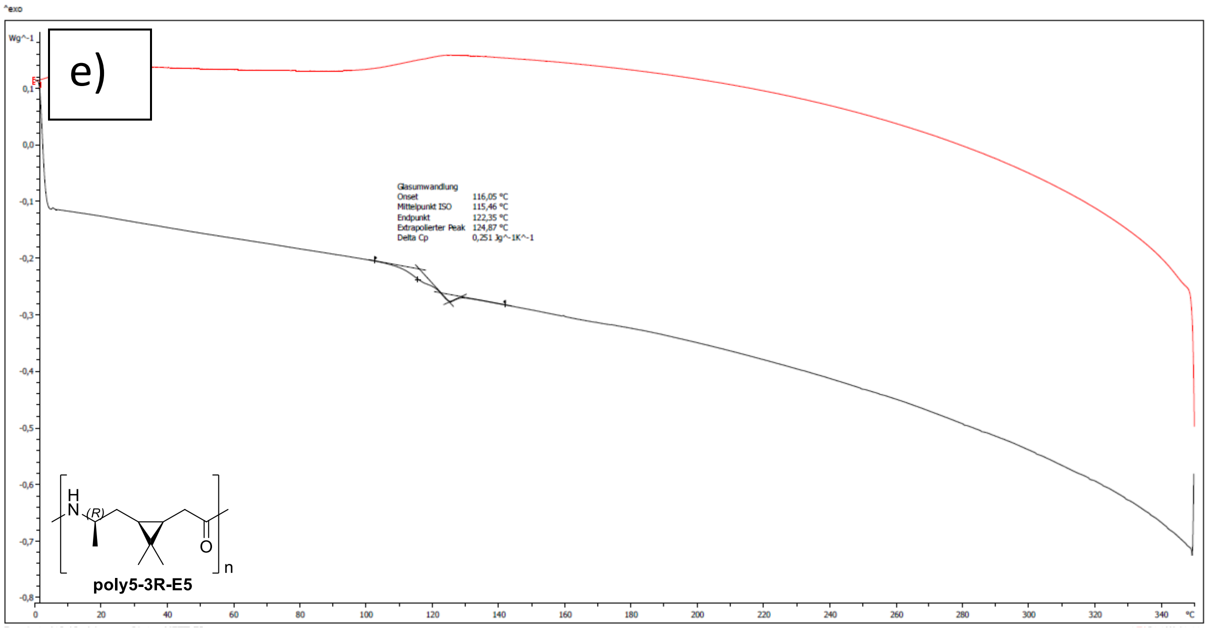
^exo
Wg^-1
e)
Glasumwandlung
Onset 116,05 °C
Mittelpunkt ISO 115,46 °C
Endpunkt 122,35 °C
Extrapolierter Peak 124,87 °C
Delta Cp 0,251 Jg^-1K^-1
poly5-3R-E5
°C

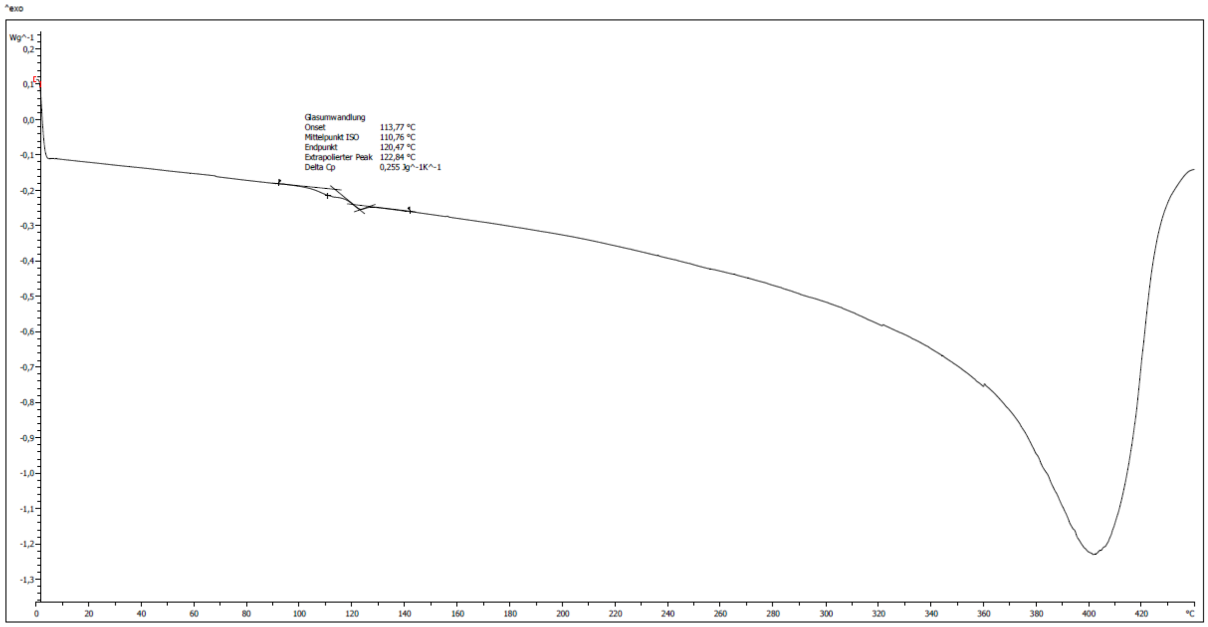
^exo
Wg^-1
Glasumwandlung
Onset 113,77 °C
Mittelpunkt ISO 110,76 °C
Endpunkt 120,47 °C
Extrapolierter Peak 122,84 °C
Delta Cp 0,255 Jg^-1K^-1
°C

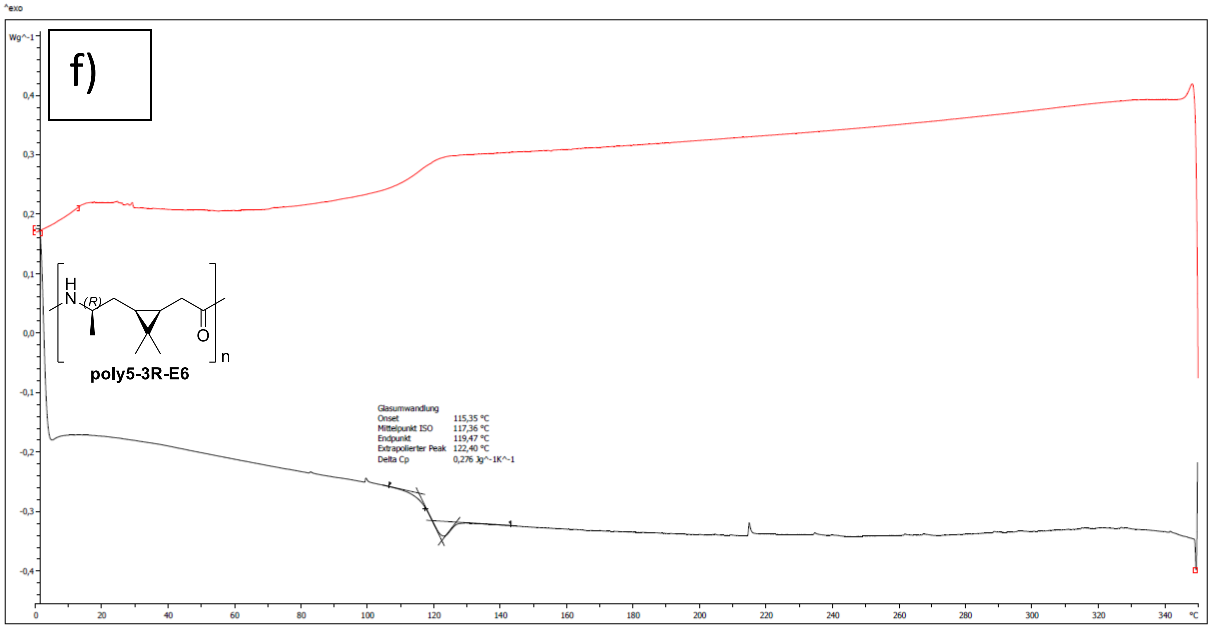

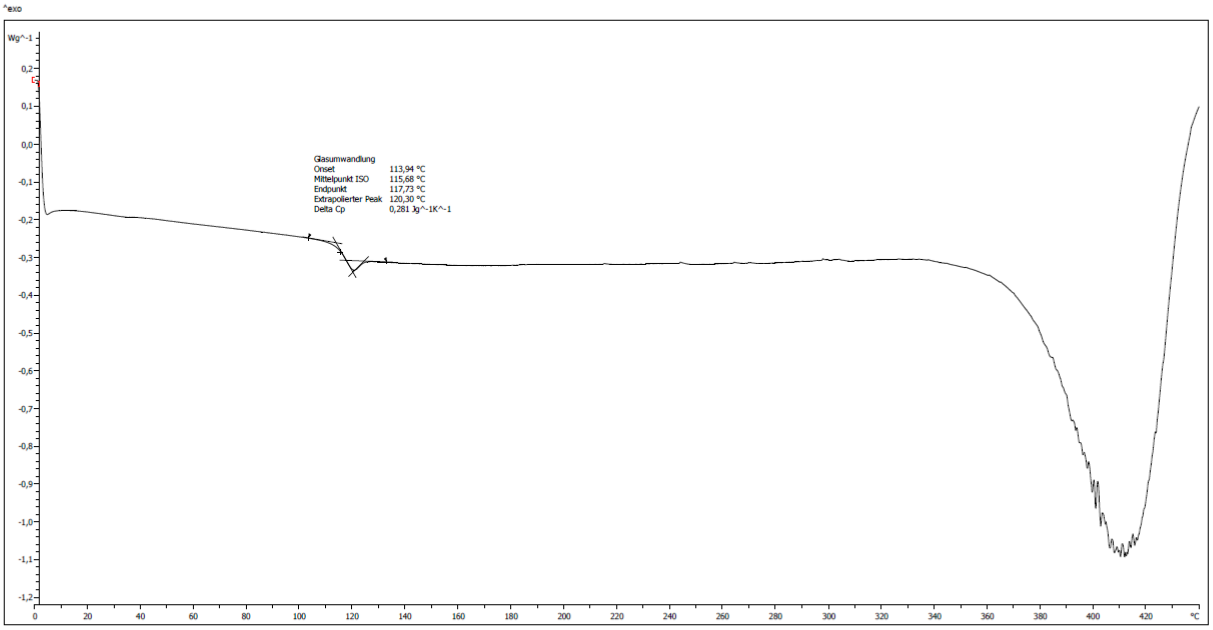

Supplementary Figure 21: DSC curves of various poly-3R-caranamides e)-f) as described in Supplementary Table 13, measured using DSC method A, segment 6 (heating, black, upper), segment 7 (cooling, red, upper) and segment 8 (black, heating to decomposition, lower).

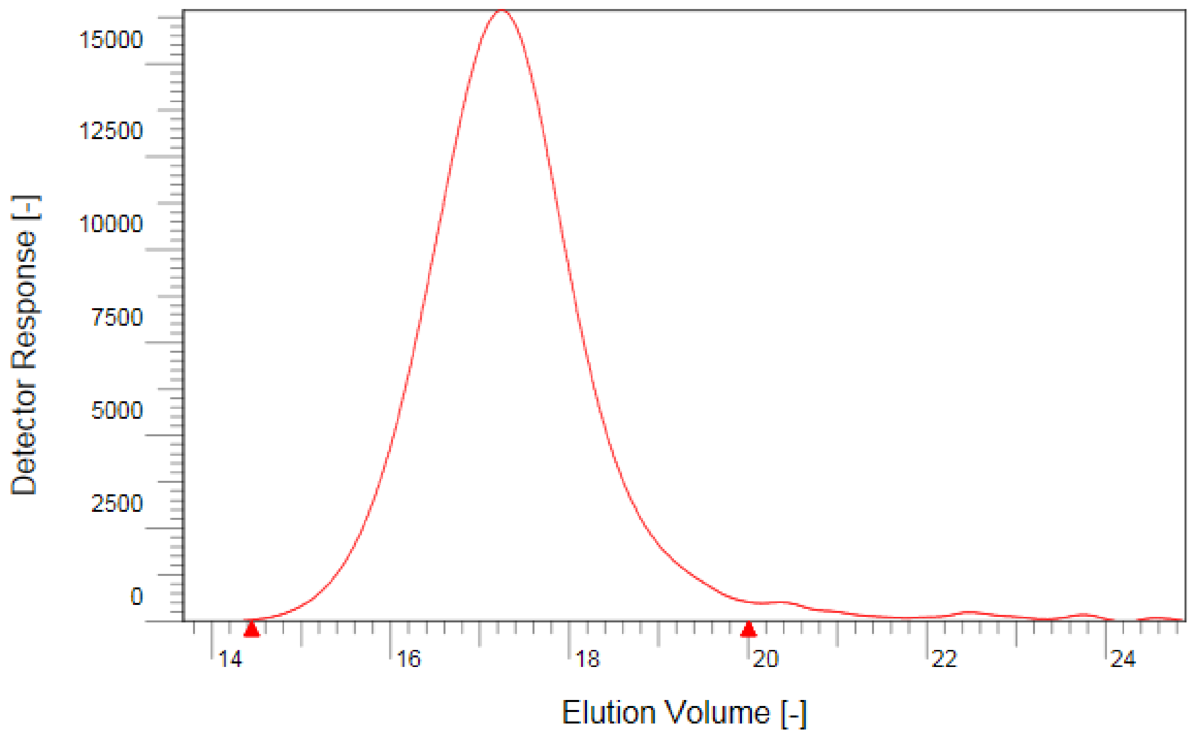

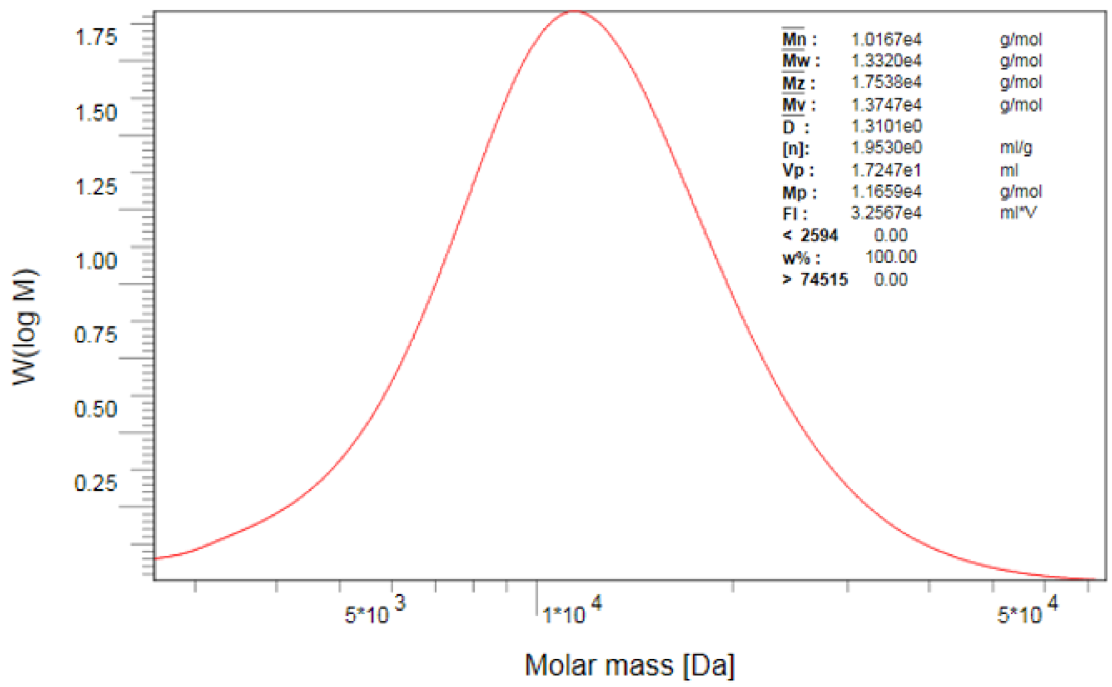

Supplementary Figure 22: GPC analysis **poly5-3R** with 10 kDa.

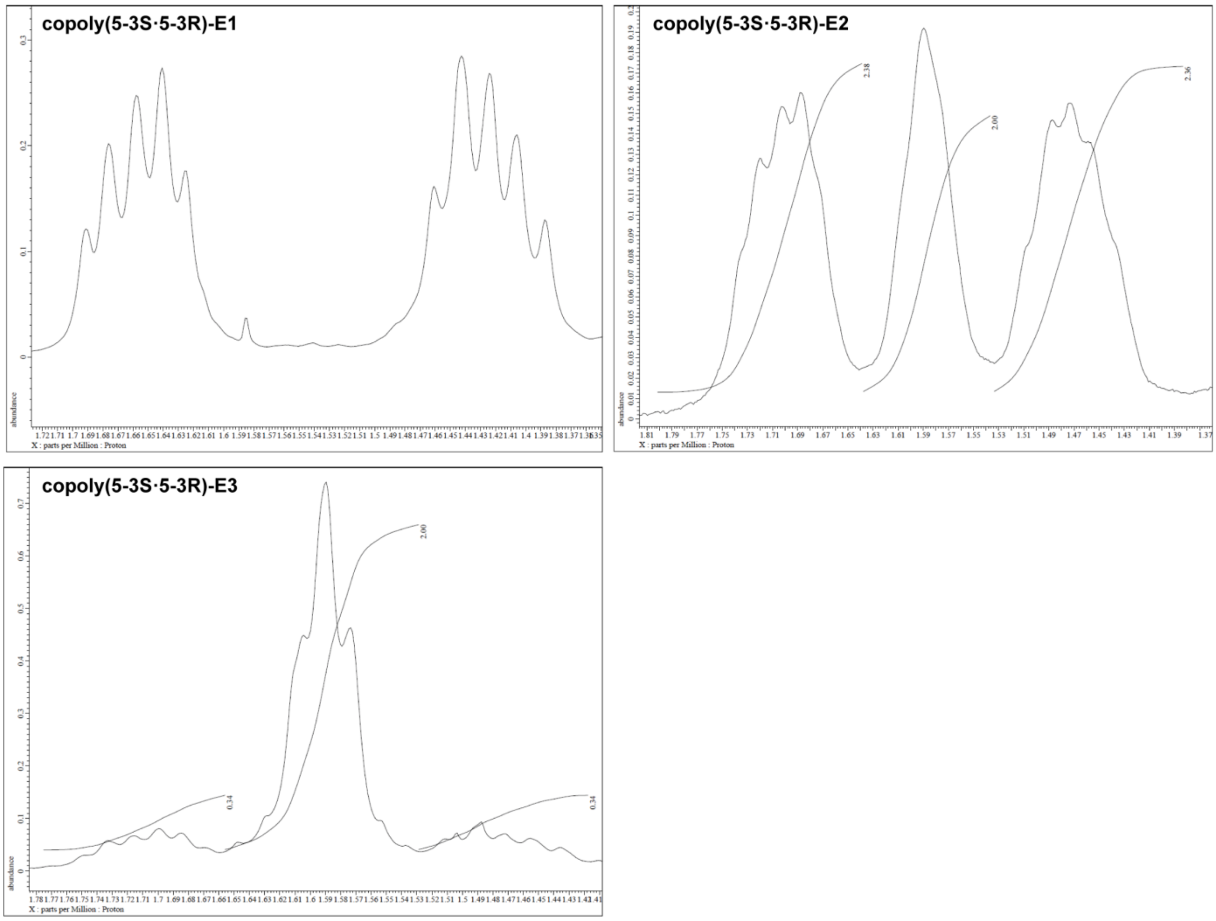

Supplementary Figure 23: Ratio of 3S-caranlactam (**5-3S**) and 3R-caranlactam (**5-3R**) in co-polymers synthesized as displayed in Supplementary Table 16 entries E1-E3.

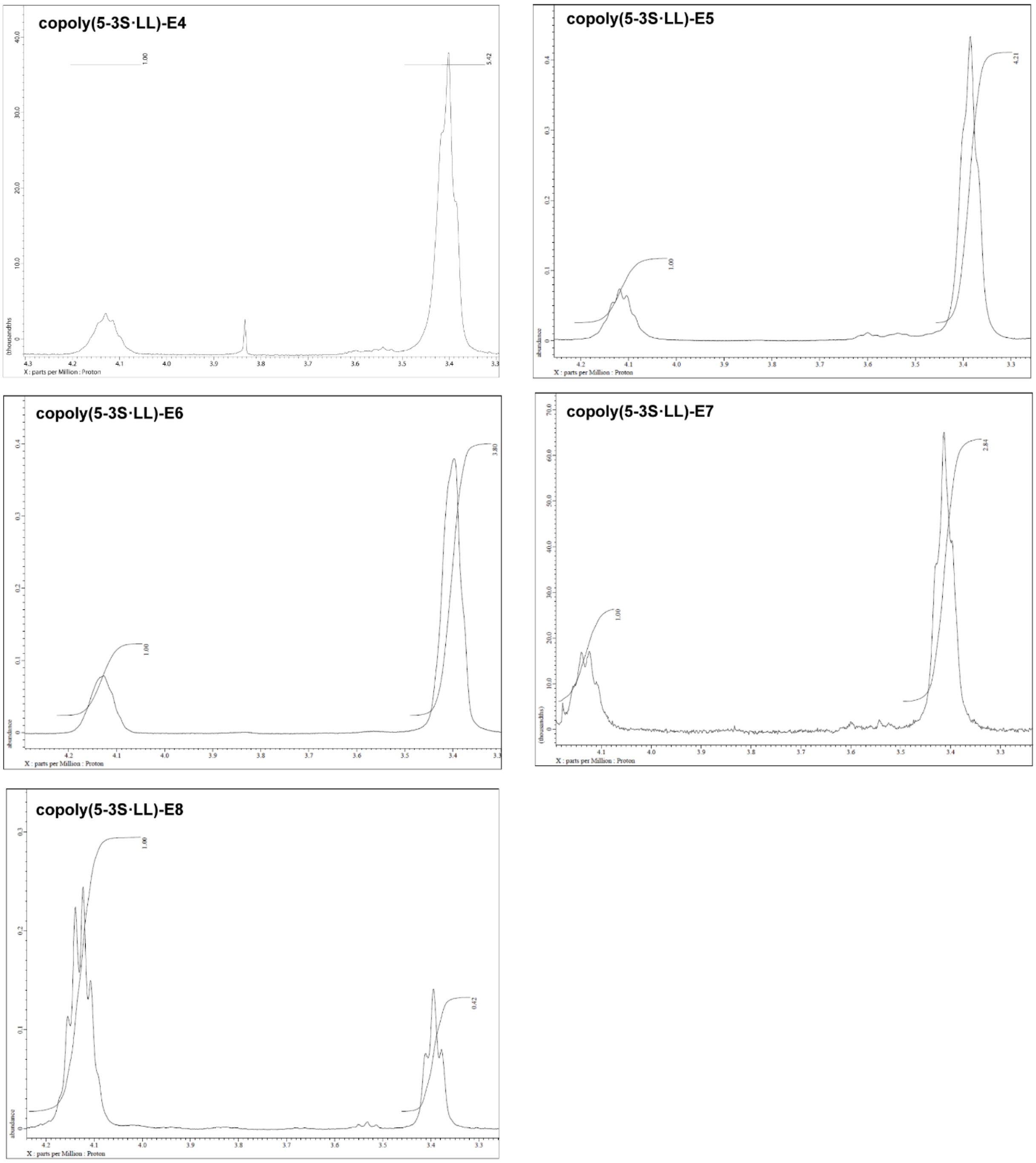

Supplementary Figure 24: Ratio of 3S-caranlactam (**5-3S**) and laurolactam (**LL**) in co-polymers synthesized as displayed in Supplementary Table 16 entries E4-E8.

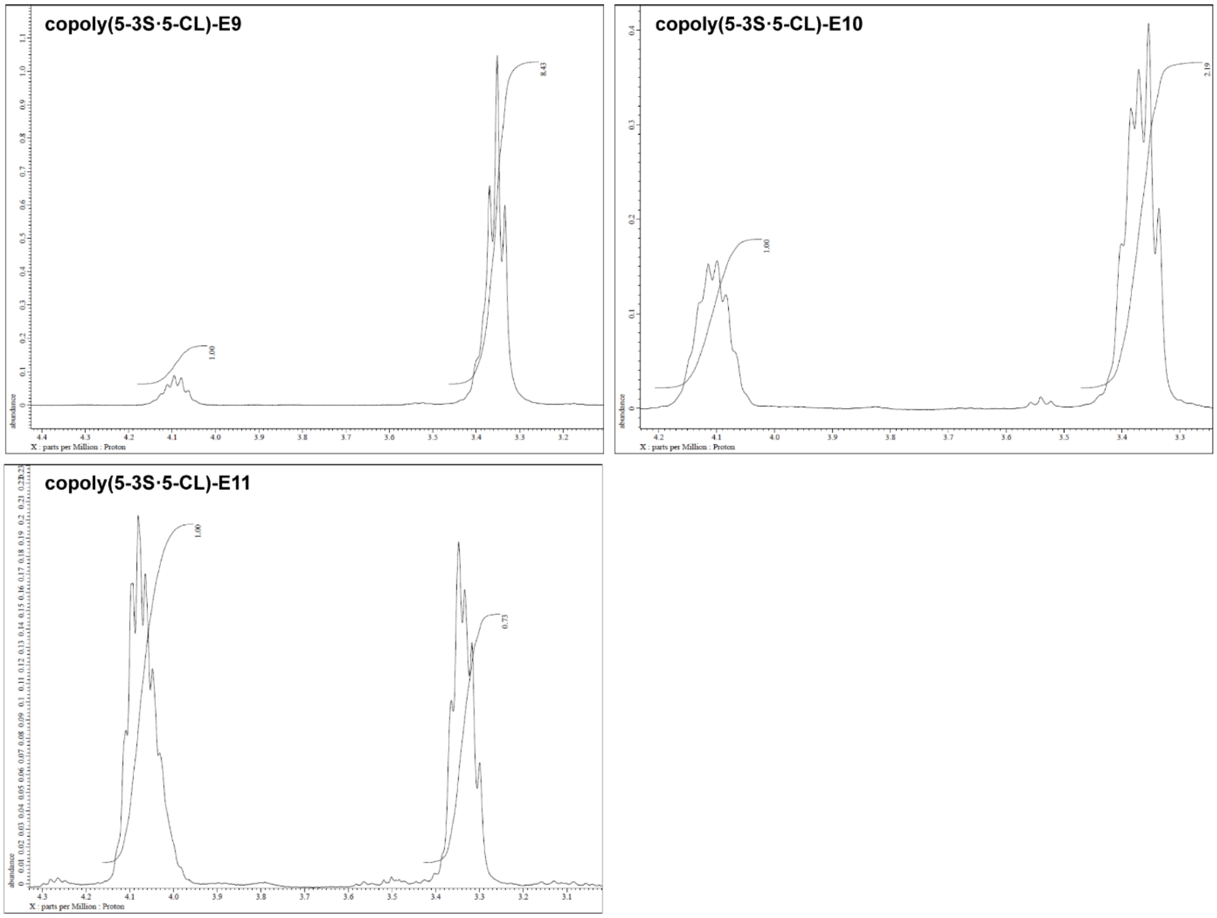

Supplementary Figure 25: Ratio of 3S-caranlactam (**5-3S**) and caprolactam (**CL**) in co-polymers synthesized as displayed in Supplementary Table 16 entries E9-E11.

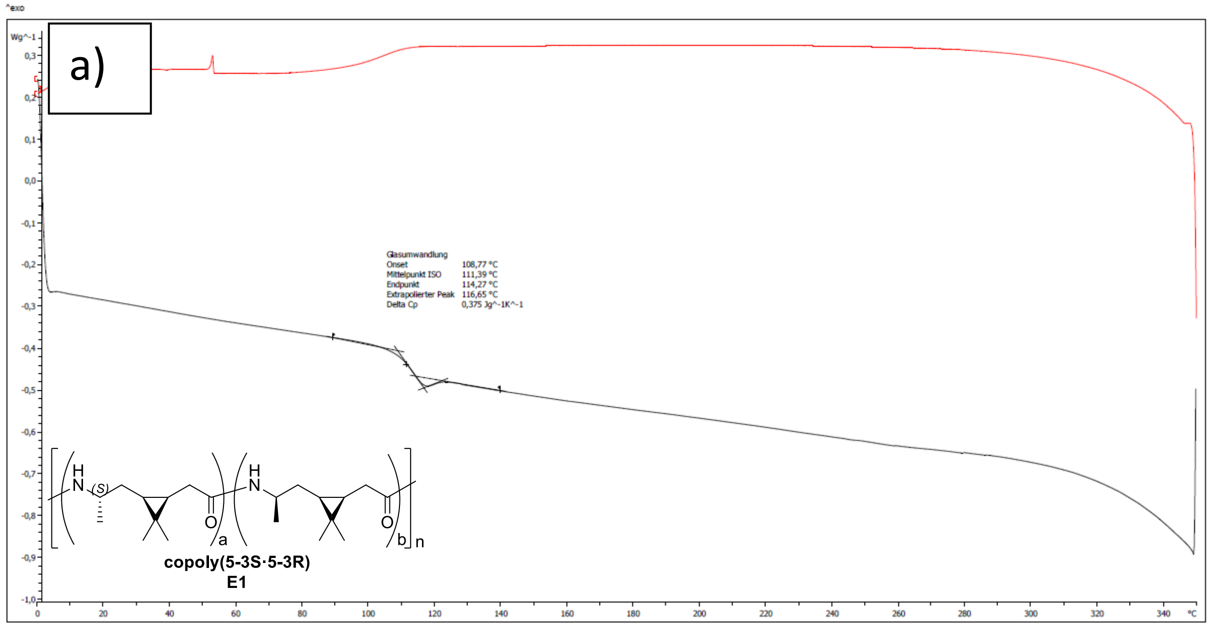

^exo
Wg^-1
a)
Glasumwandlung
Onset 108,77 °C
Mittelpunkt ISO 111,39 °C
Endpunkt 114,27 °C
Extrapolierter Peak 116,65 °C
Delta Cp 0,375 Jg^-1K^-1
copoly(5-3S·5-3R)
E1
°C

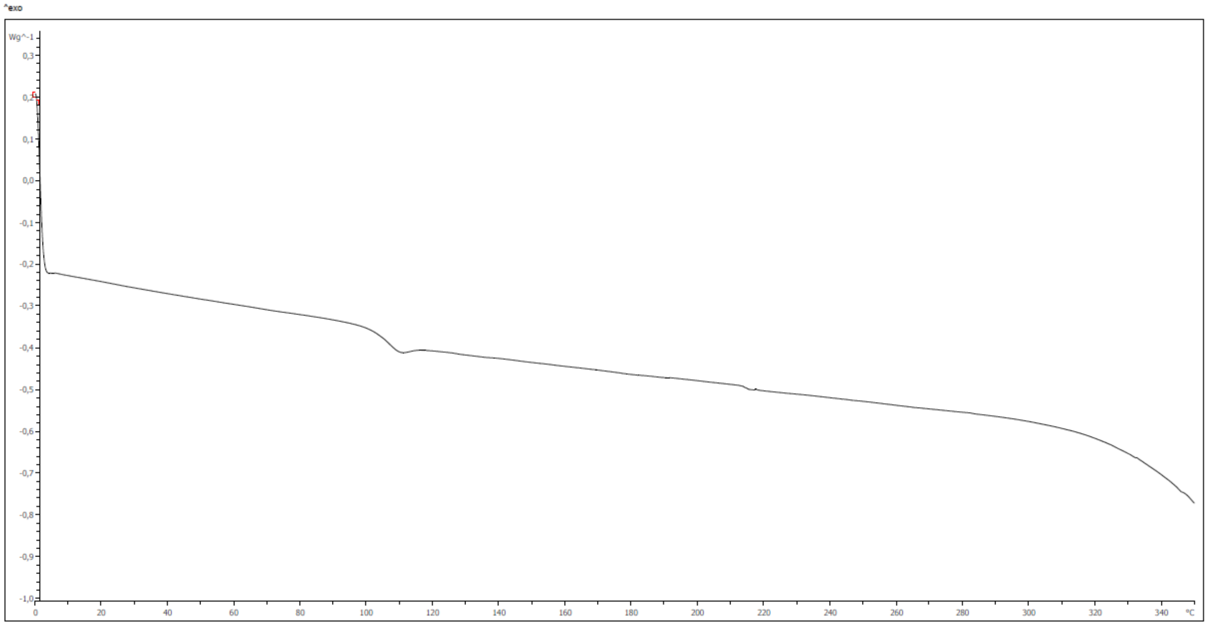

^exo
Wg^-1
°C

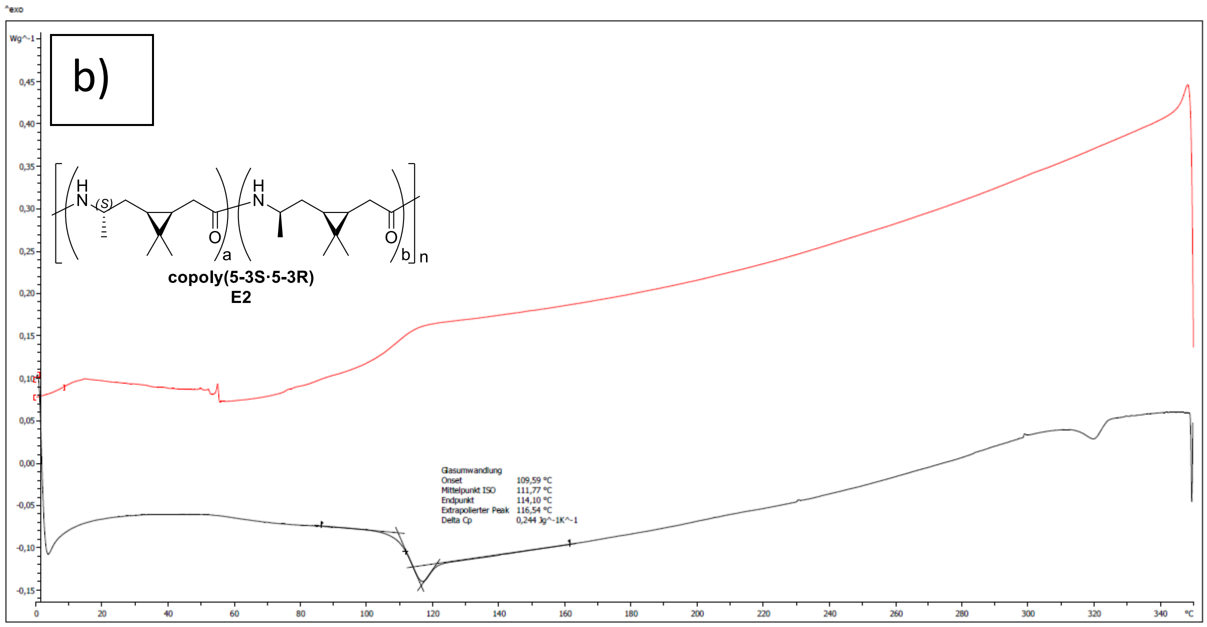

^exo
Wg^-1
b)
copoly(5-3S·5-3R)
E2
Glasumwandlung
Onset 109,59 °C
Mittelpunkt ISO 111,77 °C
Endpunkt 114,10 °C
Extrapolierter Peak 116,54 °C
Delta Cp 0,244 Jg^-1K^-1
°C

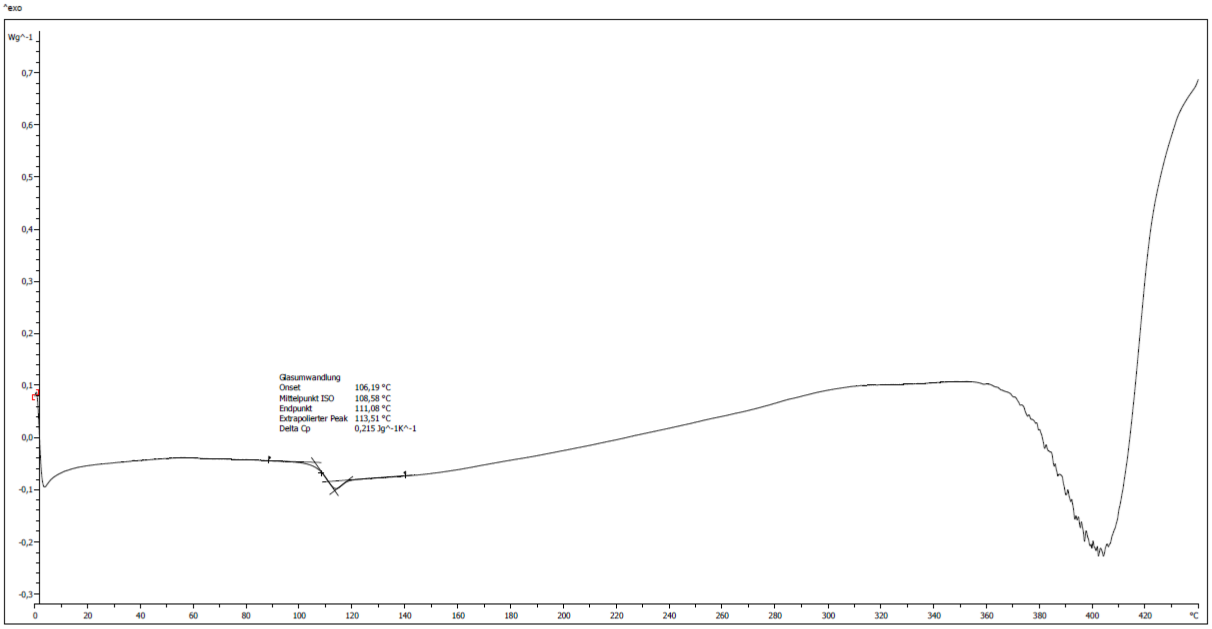

^exo
Wg^-1
Glasumwandlung
Onset 106,19 °C
Mittelpunkt ISO 108,58 °C
Endpunkt 111,08 °C
Extrapolierter Peak 113,51 °C
Delta Cp 0,215 Jg^-1K^-1
°C

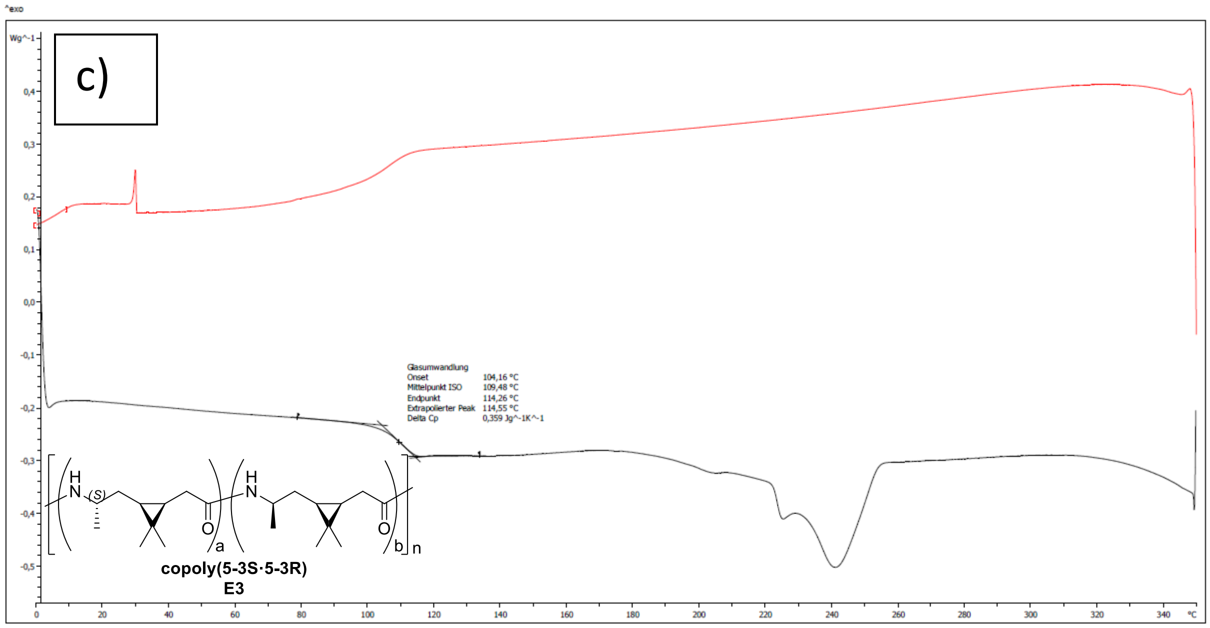

^exo
Wg^-1
c)
Glasumwandlung
Onset 104,16 °C
Mittelpunkt ISO 109,48 °C
Endpunkt 114,26 °C
Extrapolierter Peak 114,55 °C
Delta Cp 0,359 Jg^-1K^-1
copoly(5-3S·5-3R)
E3
°C

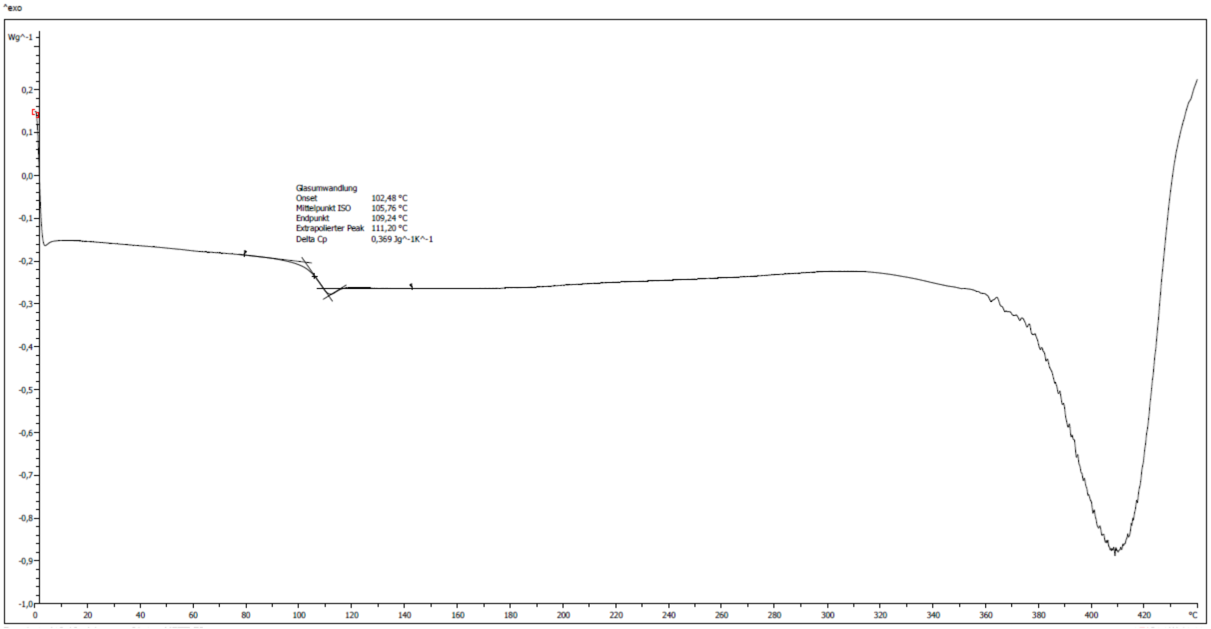

^exo
Wg^-1
Glasumwandlung
Onset 102,48 °C
Mittelpunkt ISO 105,76 °C
Endpunkt 109,24 °C
Extrapolierter Peak 111,20 °C
Delta Cp 0,369 Jg^-1K^-1
°C

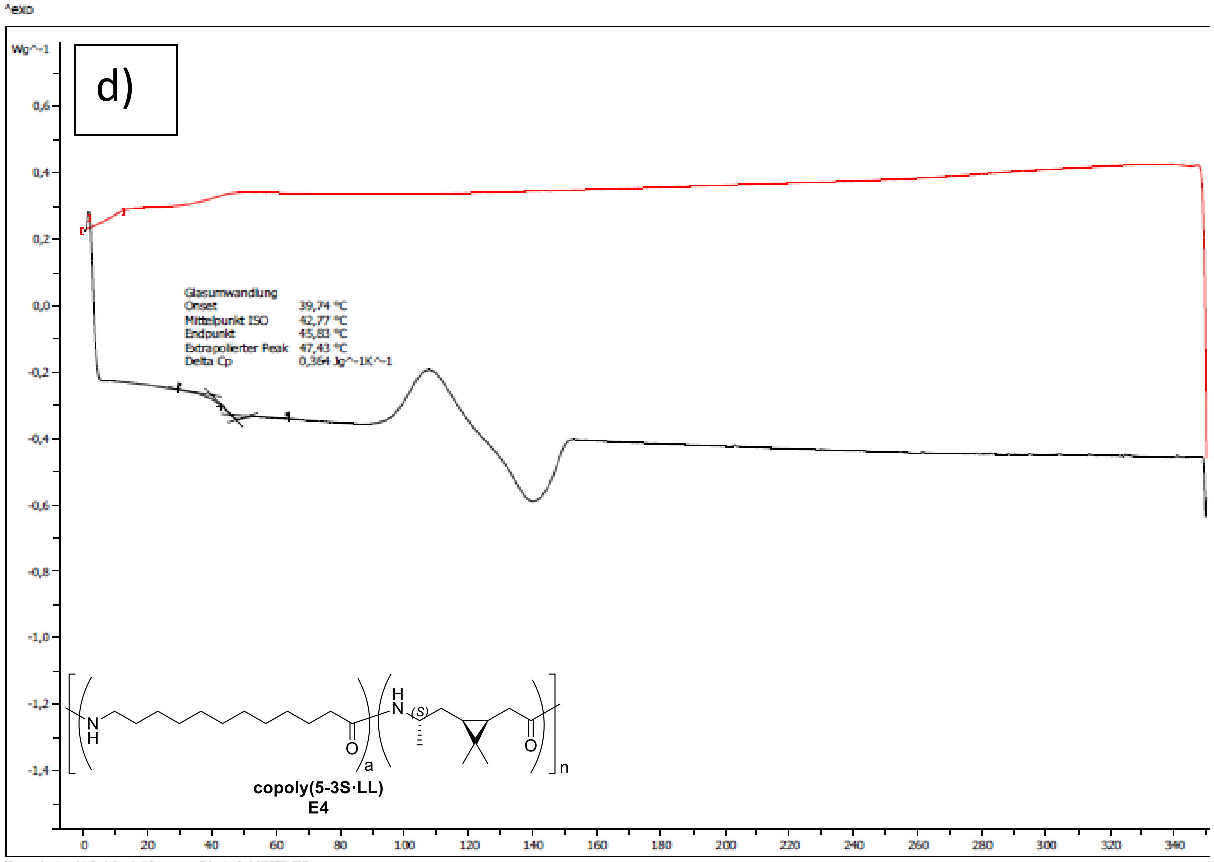
^exo
Wg^-1
d)
Glasumwandlung
Onset 39,74 °C
Mittelpunkt ISO 42,77 °C
Endpunkt 45,83 °C
Extrapolierter Peak 47,43 °C
Delta Cp 0,364 Jg^-1K^-1
copoly(5-3S·LL)
E4

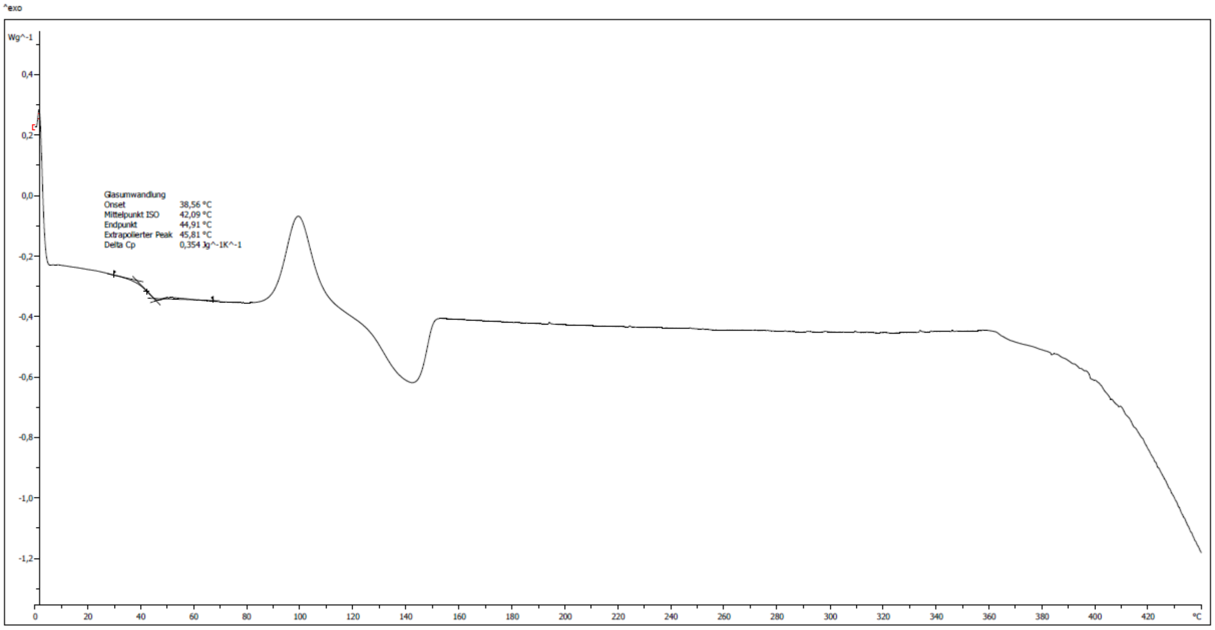
^exo
Wg^-1
Glasumwandlung
Onset 38,56 °C
Mittelpunkt ISO 42,09 °C
Endpunkt 44,91 °C
Extrapolierter Peak 45,81 °C
Delta Cp 0,354 Jg^-1K^-1
°C

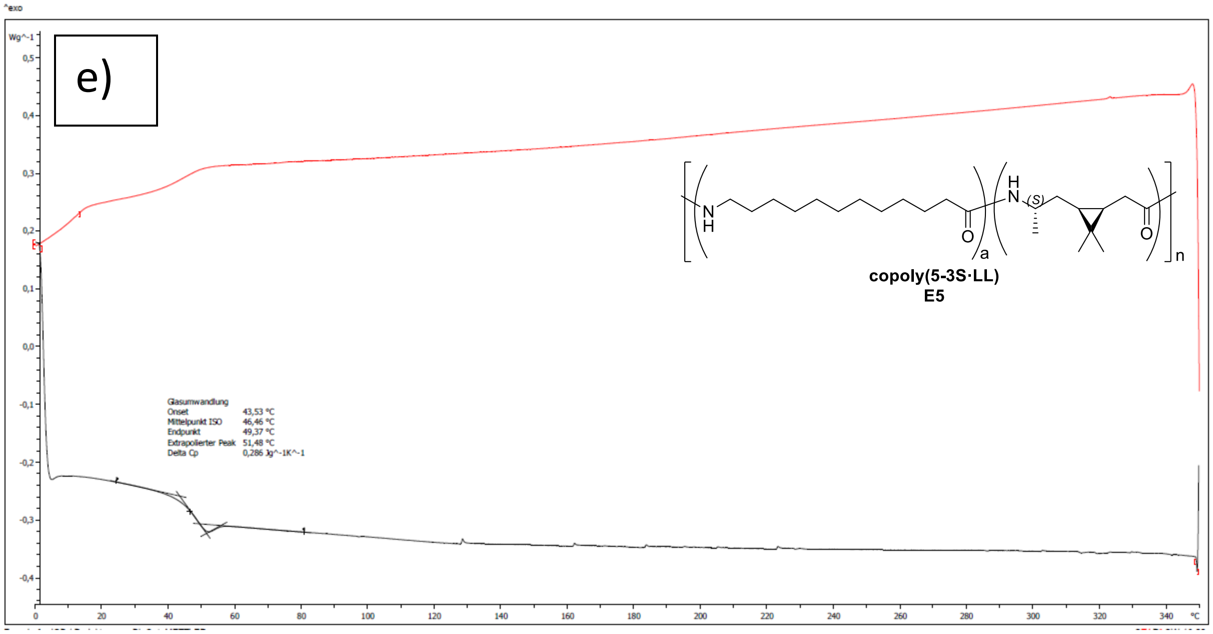
^exo
Wg^-1
e)
copoly(5-3S·LL)
E5
Glasumwandlung
Onset 43,53 °C
Mittelpunkt ISO 46,46 °C
Endpunkt 49,37 °C
Extrapolierter Peak 51,48 °C
Delta Cp 0,286 Jg^-1K^-1
°C

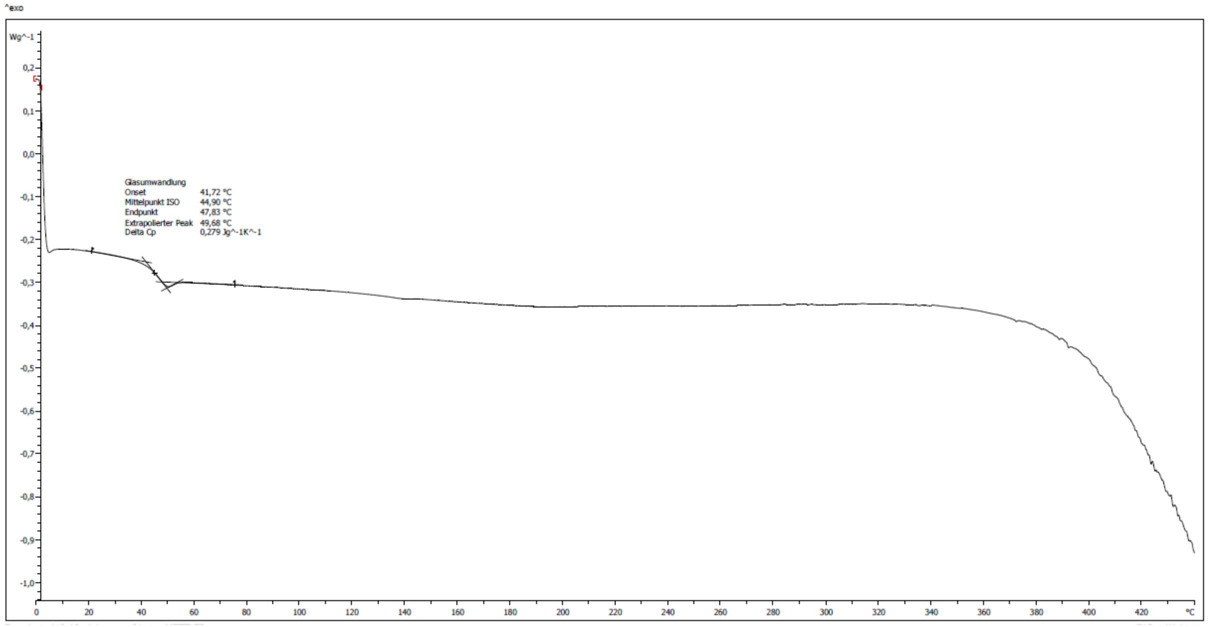
^exo
Wg^-1
Glasumwandlung
Onset 41,72 °C
Mittelpunkt ISO 44,90 °C
Endpunkt 47,83 °C
Extrapolierter Peak 49,68 °C
Delta Cp 0,279 Jg^-1K^-1
°C

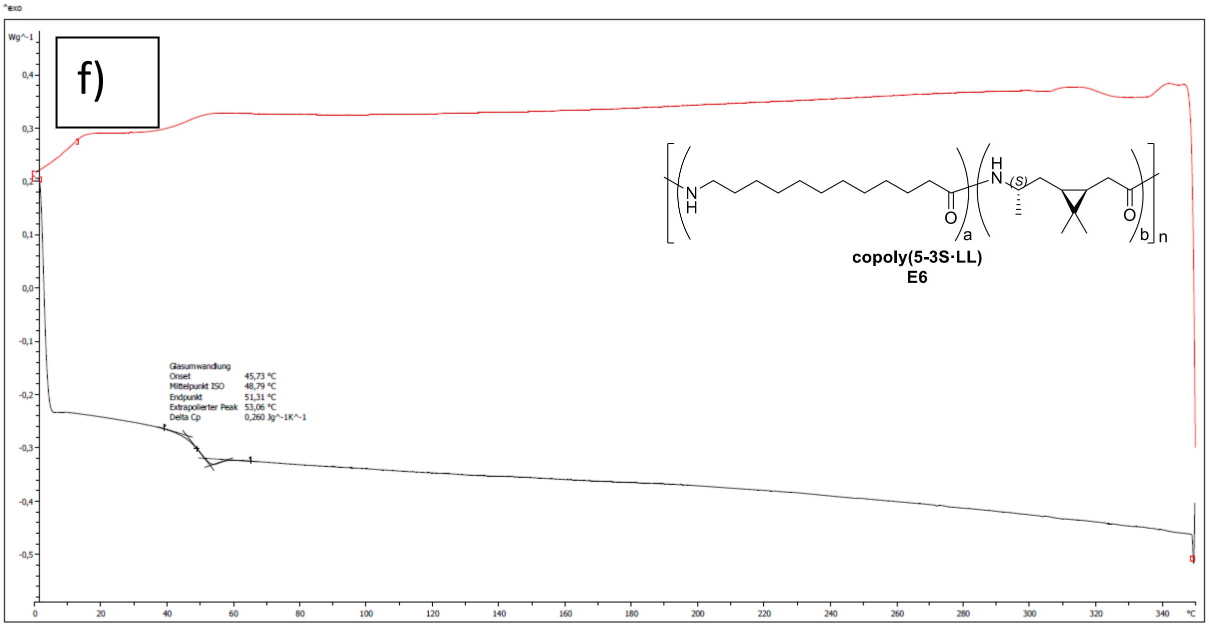

^exo
Wg^-1
f)
copoly(5-3S·LL)
E6
Glasumwandlung
Onset 45,73 °C
Mittelpunkt ISO 48,79 °C
Endpunkt 51,31 °C
Extrapolierter Peak 53,06 °C
Delta Cp 0,260 Jg^-1K^-1
°C

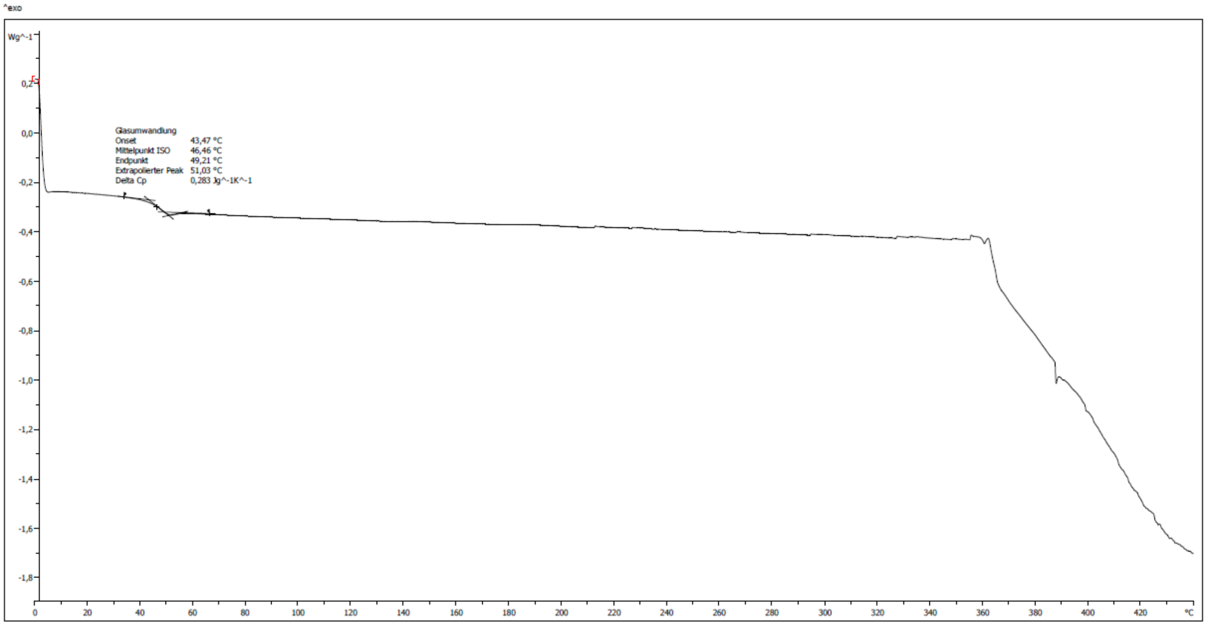

^exo
Wg^-1
Glasumwandlung
Onset 43,47 °C
Mittelpunkt ISO 46,46 °C
Endpunkt 49,21 °C
Extrapolierter Peak 51,03 °C
Delta Cp 0,283 Jg^-1K^-1
°C

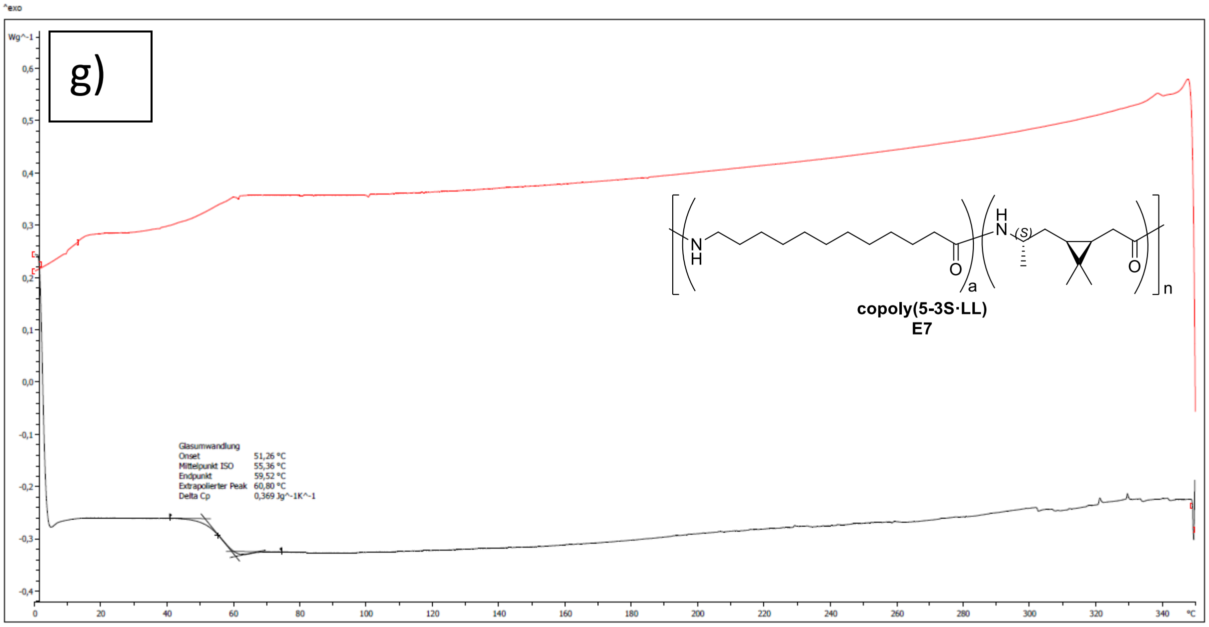
^exo
Wg^-1
g)
copoly(5-3S·LL)
E7
Glasumwandlung
Onset 51,26 °C
Mittelpunkt ISO 55,36 °C
Endpunkt 59,52 °C
Extrapolierter Peak 60,80 °C
Delta Cp 0,369 Jg^-1K^-1
°C

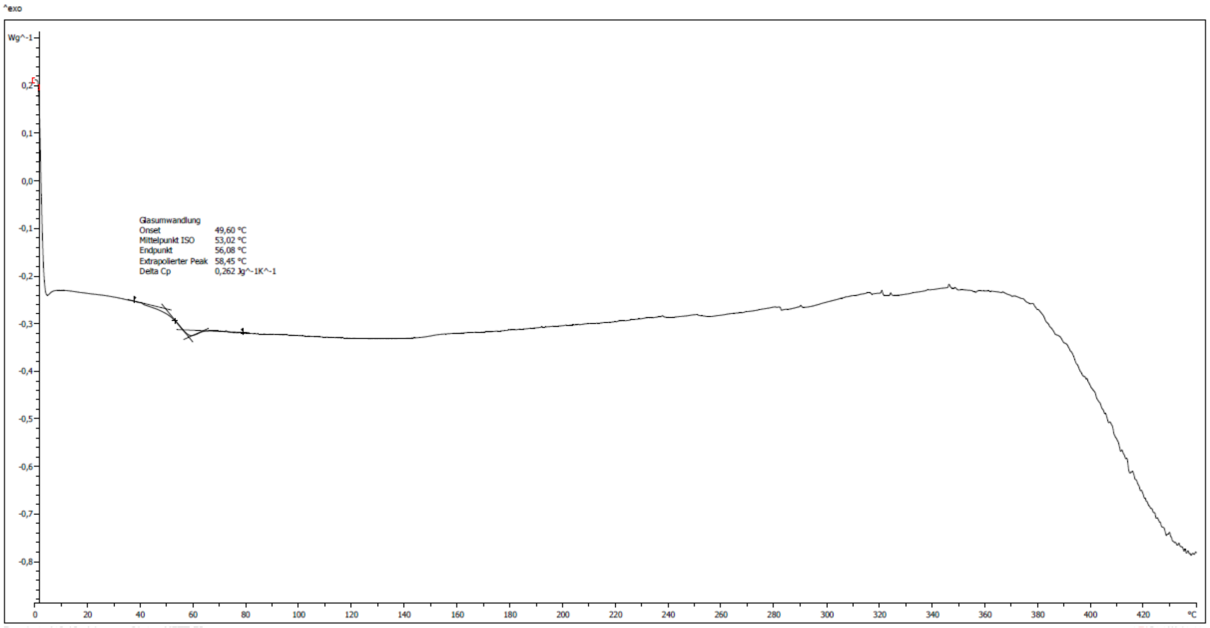
^exo
Wg^-1
Glasumwandlung
Onset 49,60 °C
Mittelpunkt ISO 53,02 °C
Endpunkt 56,08 °C
Extrapolierter Peak 58,45 °C
Delta Cp 0,262 Jg^-1K^-1
°C

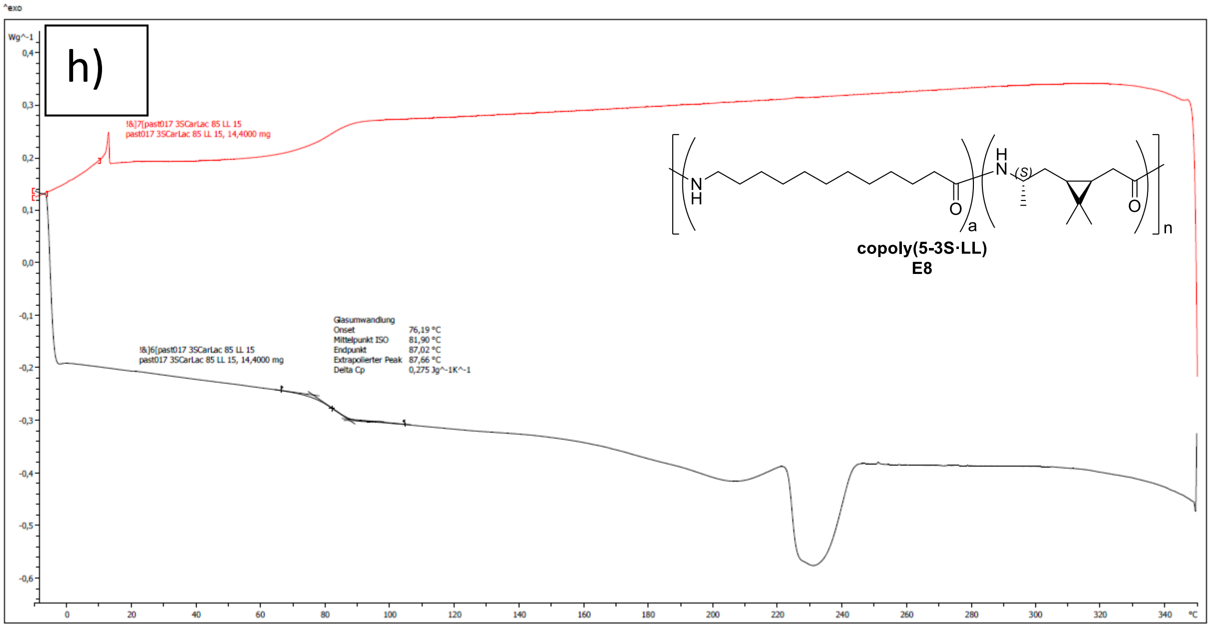
^exo
Wg^-1
h)
!&]7[past017 3SCarLac 85 LL 15
past017 3SCarLac 85 LL 15, 14,4000 mg
!&]6[past017 3SCarLac 85 LL 15
past017 3SCarLac 85 LL 15, 14,4000 mg
Glasumwandlung
Onset 76,19 °C
Mittelpunkt ISO 81,90 °C
Endpunkt 87,02 °C
Extrapolierter Peak 87,66 °C
Delta Cp 0,275 Jg^-1K^-1
copoly(5-3S·LL)
E8
°C

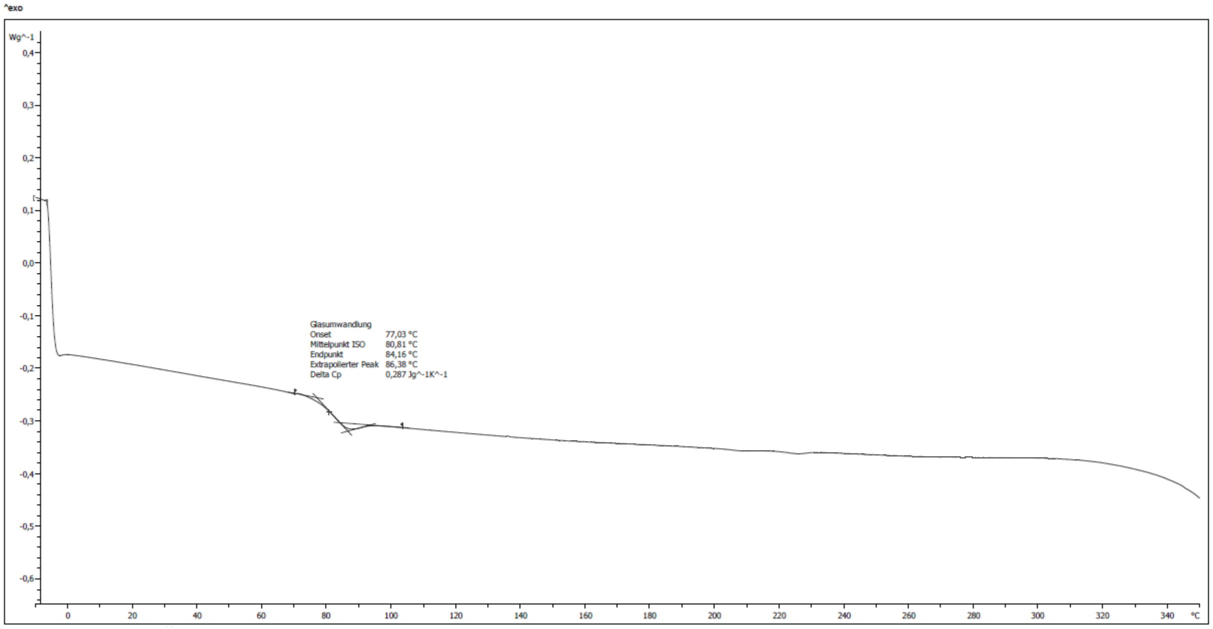
^exo
Wg^-1
Glasumwandlung
Onset 77,03 °C
Mittelpunkt ISO 80,81 °C
Endpunkt 84,16 °C
Extrapolierter Peak 86,38 °C
Delta Cp 0,287 Jg^-1K^-1
°C

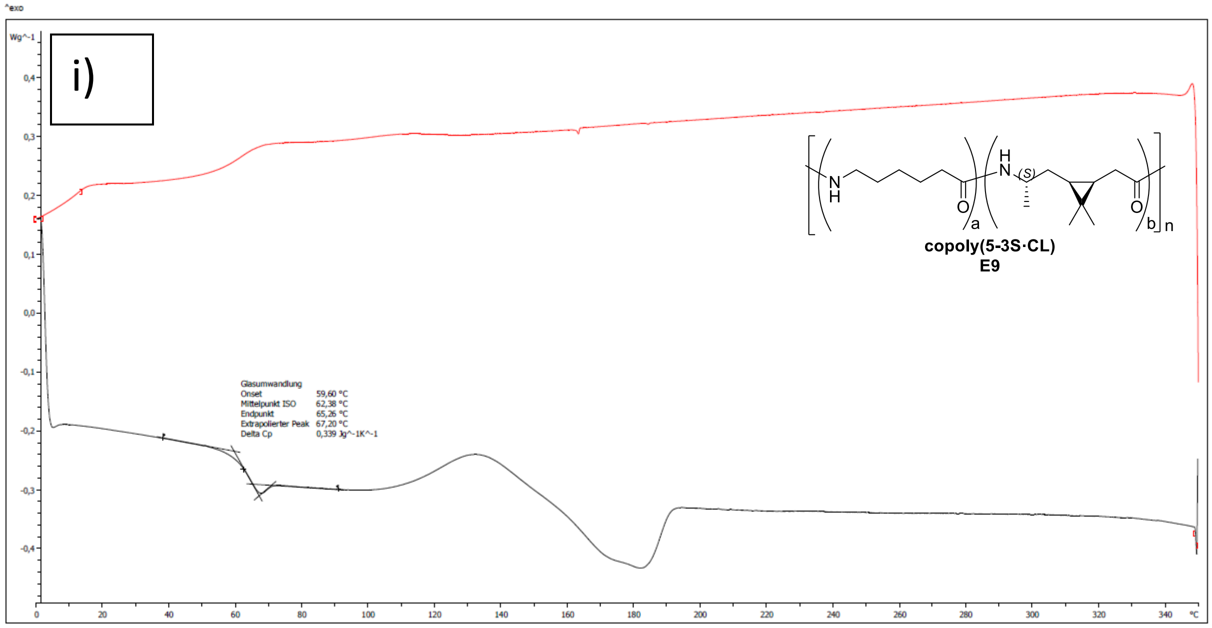

^exo
Wg^-1
i)
copoly(5-3S·CL)
E9
Glasumwandlung
Onset 59,60 °C
Mittelpunkt ISO 62,38 °C
Endpunkt 65,26 °C
Extrapolierter Peak 67,20 °C
Delta Cp 0,339 Jg^-1K^-1
°C

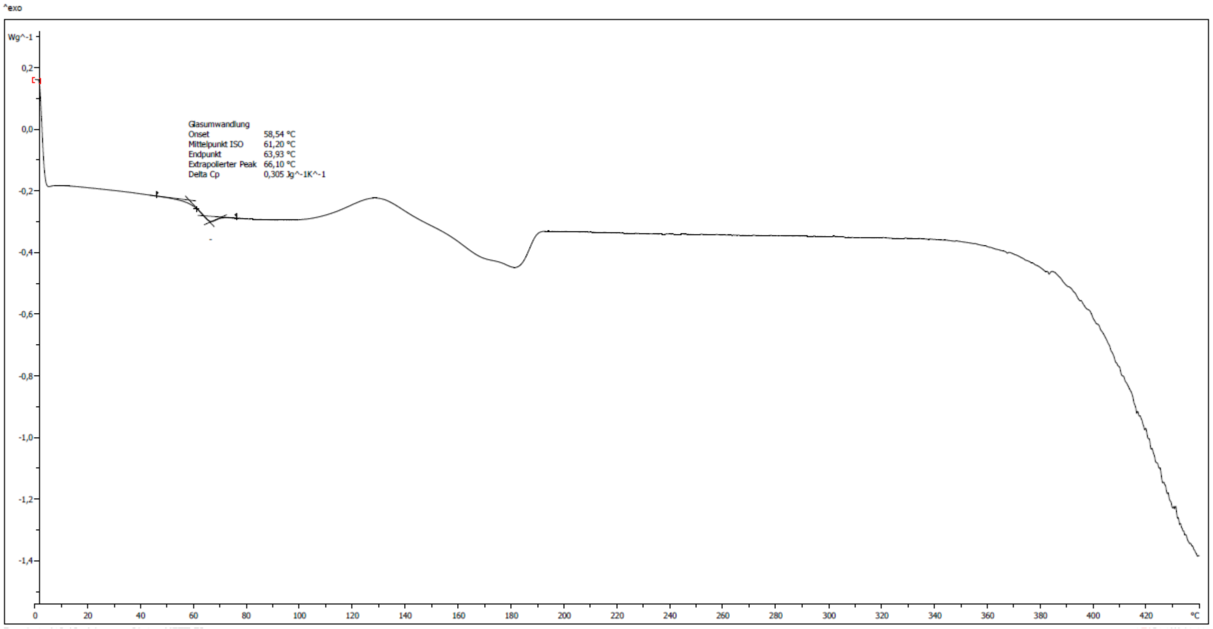

^exo
Wg^-1
Glasumwandlung
Onset 58,54 °C
Mittelpunkt ISO 61,20 °C
Endpunkt 63,93 °C
Extrapolierter Peak 66,10 °C
Delta Cp 0,305 Jg^-1K^-1
°C

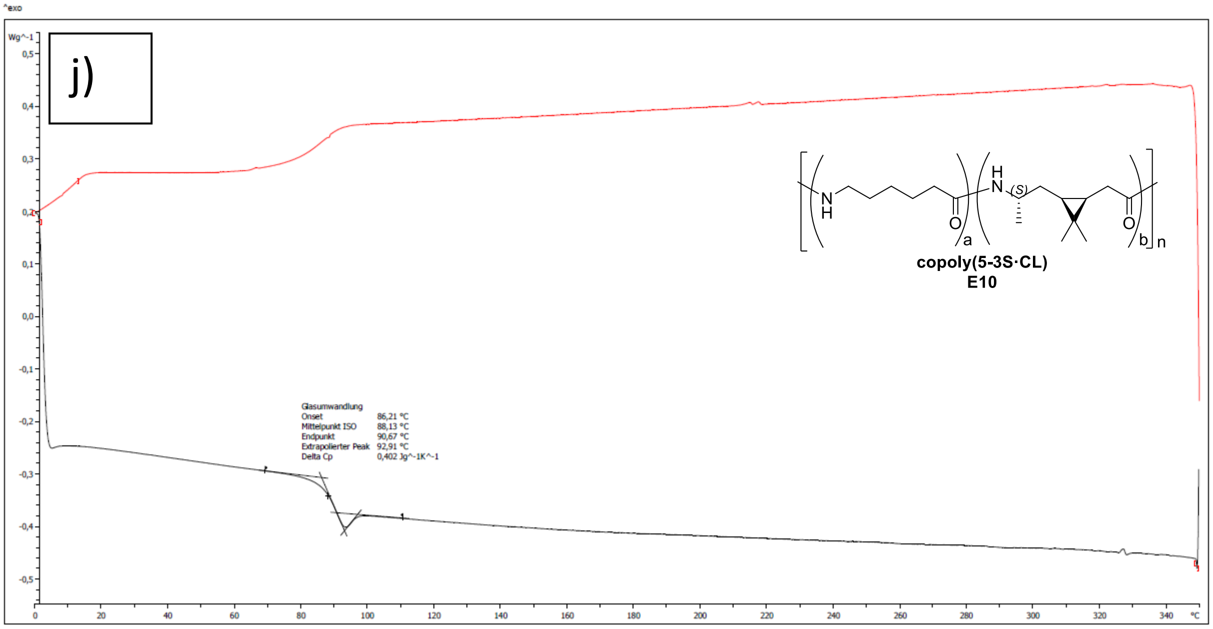
^exo
Wg^-1
j)
copoly(5-3S·CL)
E10
Glasumwandlung
Onset 86,21 °C
Mittelpunkt ISO 88,13 °C
Endpunkt 90,67 °C
Extrapolierter Peak 92,91 °C
Delta Cp 0,402 Jg^-1K^-1
°C

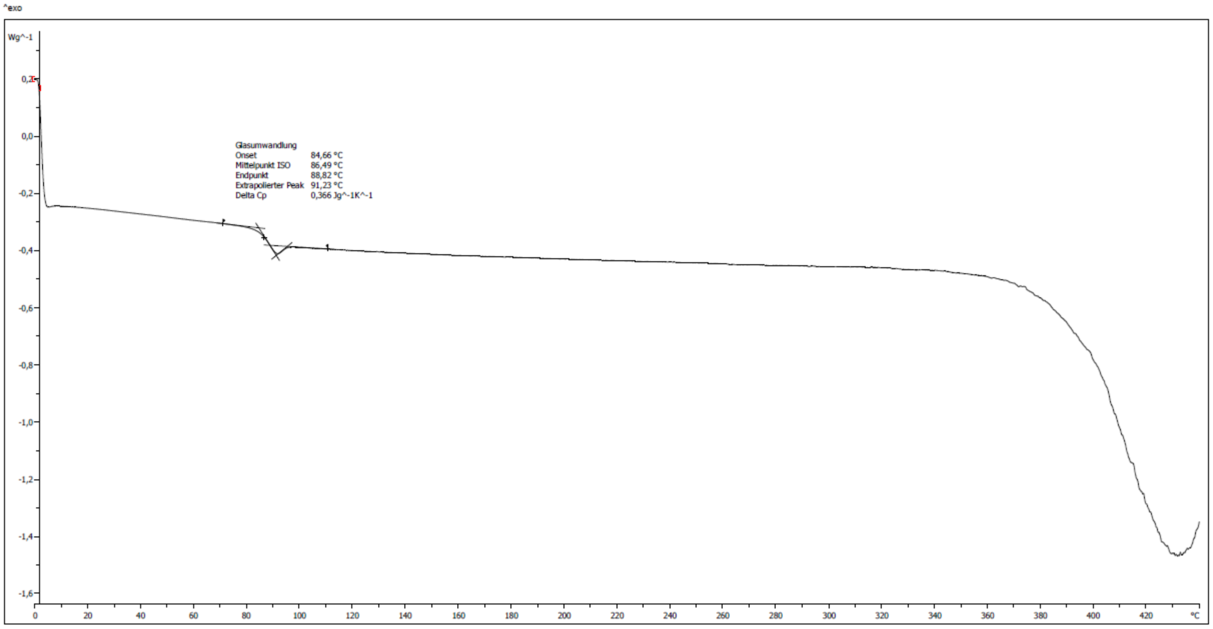
^exo
Wg^-1
Glasumwandlung
Onset 84,66 °C
Mittelpunkt ISO 86,49 °C
Endpunkt 88,82 °C
Extrapolierter Peak 91,23 °C
Delta Cp 0,366 Jg^-1K^-1
°C

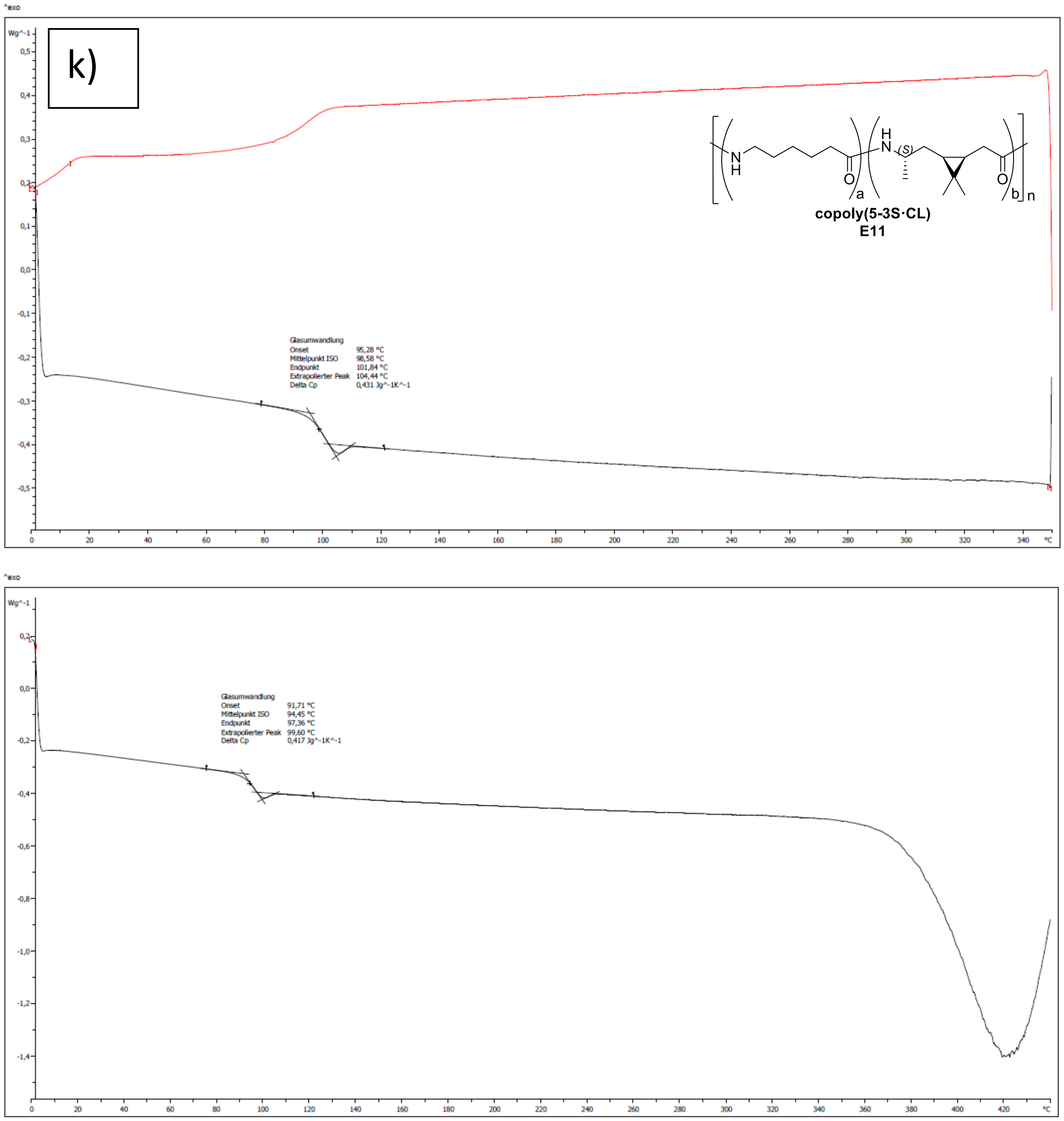

Supplementary Figure 26: DSC curves of various co-polyamides a)-k) as described in Supplementary Table 16, measured using DSC method A, segment 6 (heating, black, upper), segment 7 (cooling, red, upper) and segment 8 (black, heating to decomposition, lower).

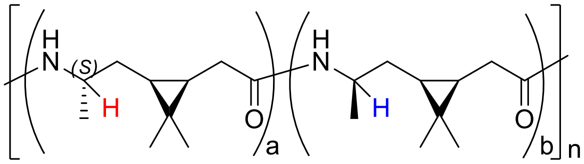

copoly(5-3S·5-3R)

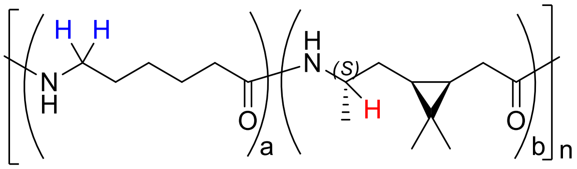

copoly(5-3S·CL)

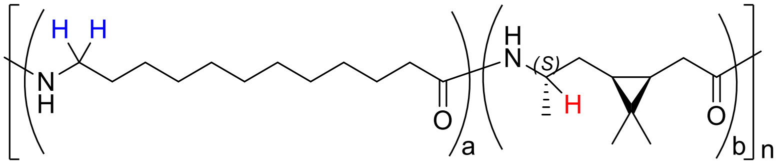

copoly(5-3S·LL)

Supplementary Figure 27: Selected protons (red: **5-3S**, blue: co-monomer) for the determination of different built-ins under various reaction conditions by NMR.

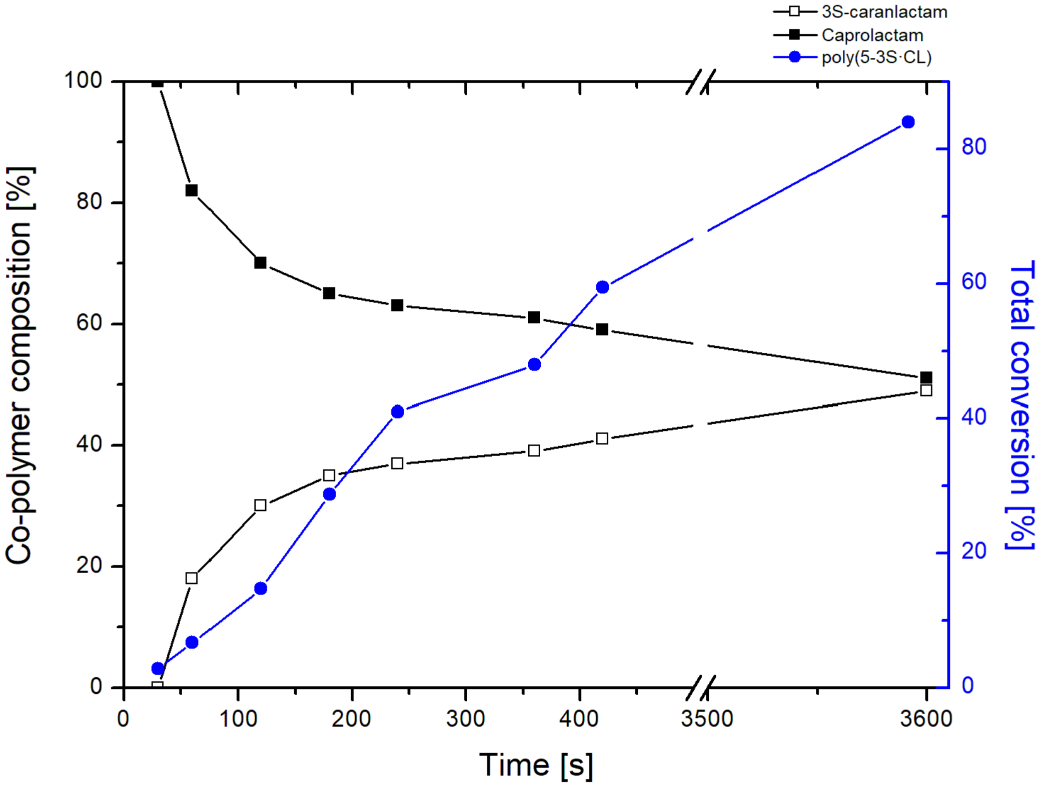

Supplementary Figure 28: Incorporation of **5-3S** and **CL** at different reaction times. Reaction conditions: 3S-caranlactam (**5-3S**, 2.36 g, 14.1 mmol), caprolactam (**CL**, 1.64 g, 14.5 mmol, NaH (60% on paraffin, 10.0 mg, 0.25 mmol) and **Bz5-3S** (54.0 mg, 0.22 mmol), 190 °C

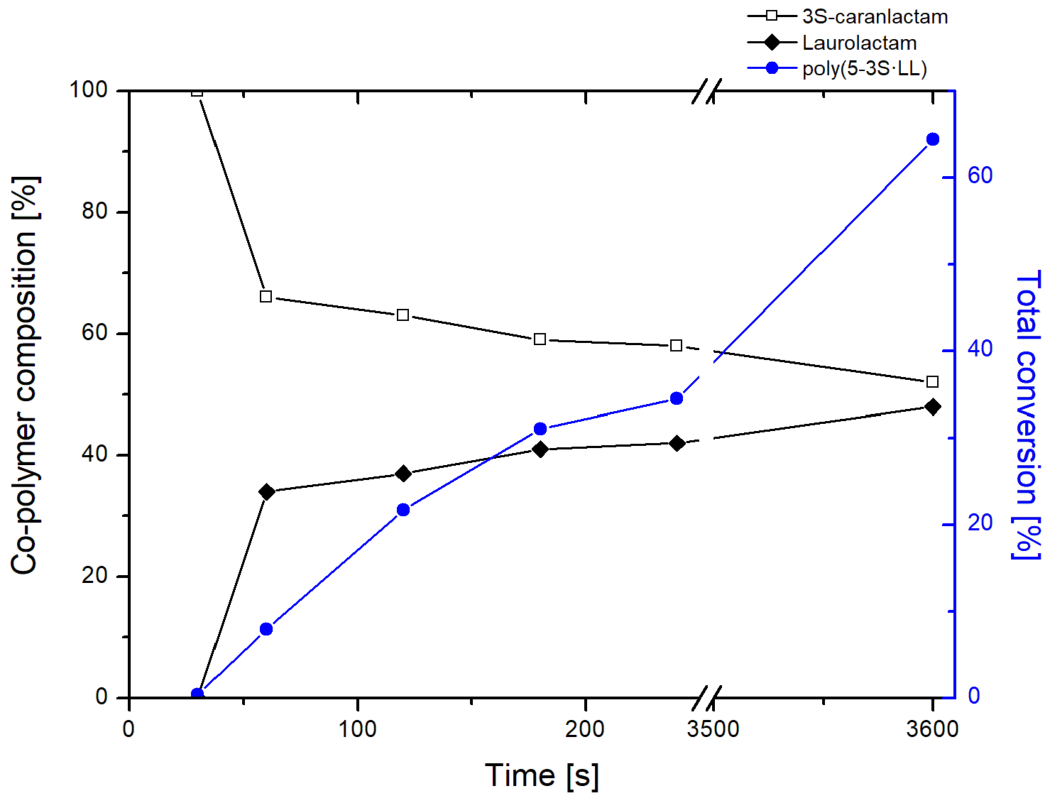

Supplementary Figure 29: Incorporation of **5-3S** and **LL** at different reaction times. Reaction conditions: 3S-caranlactam (**5-3S**, 2.39 g, 14.3 mmol), laurolactam (**LL**, 2.82 g, 14.3 mmol, NaH (60% on paraffin, 9.6 mg, 0.24 mmol) and **Bz5-3S** (56.0 mg, 0.23 mmol), 190 °C.

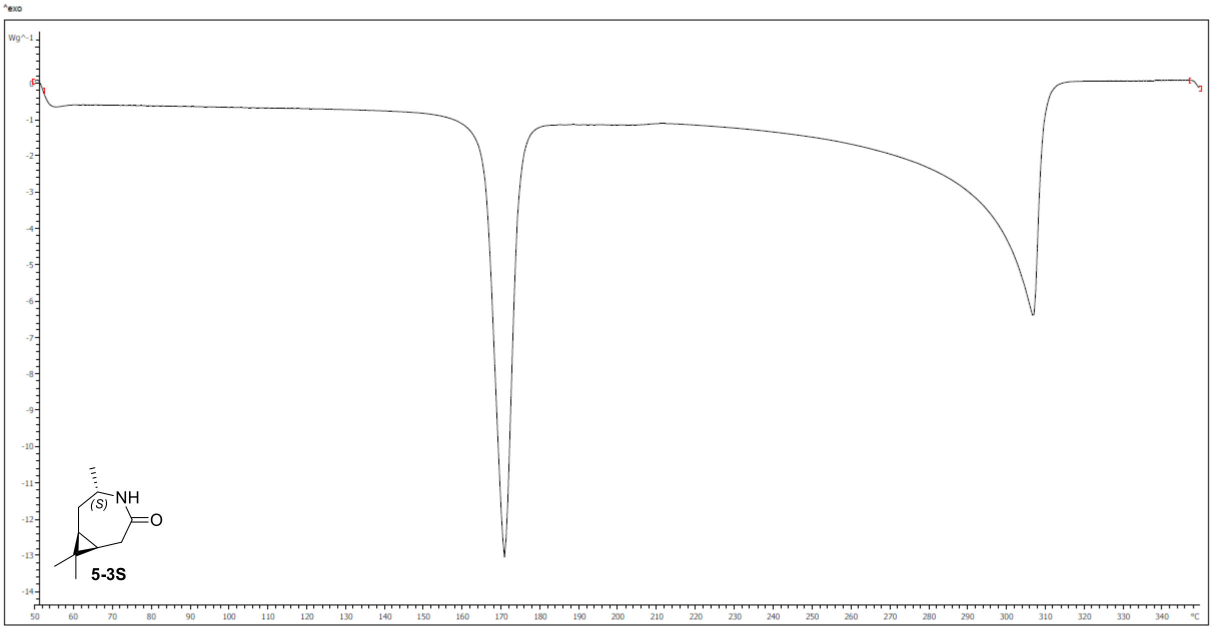

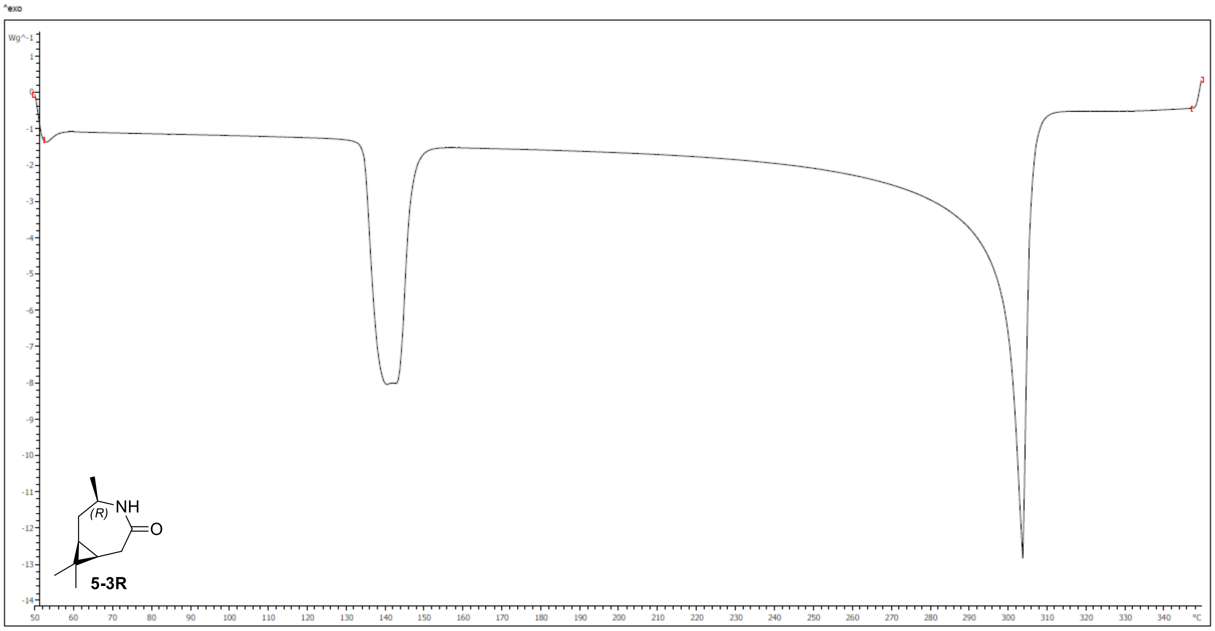

Supplementary Figure 30: DSC curves of **5-3S** (upper) and **5-3R** (lower).

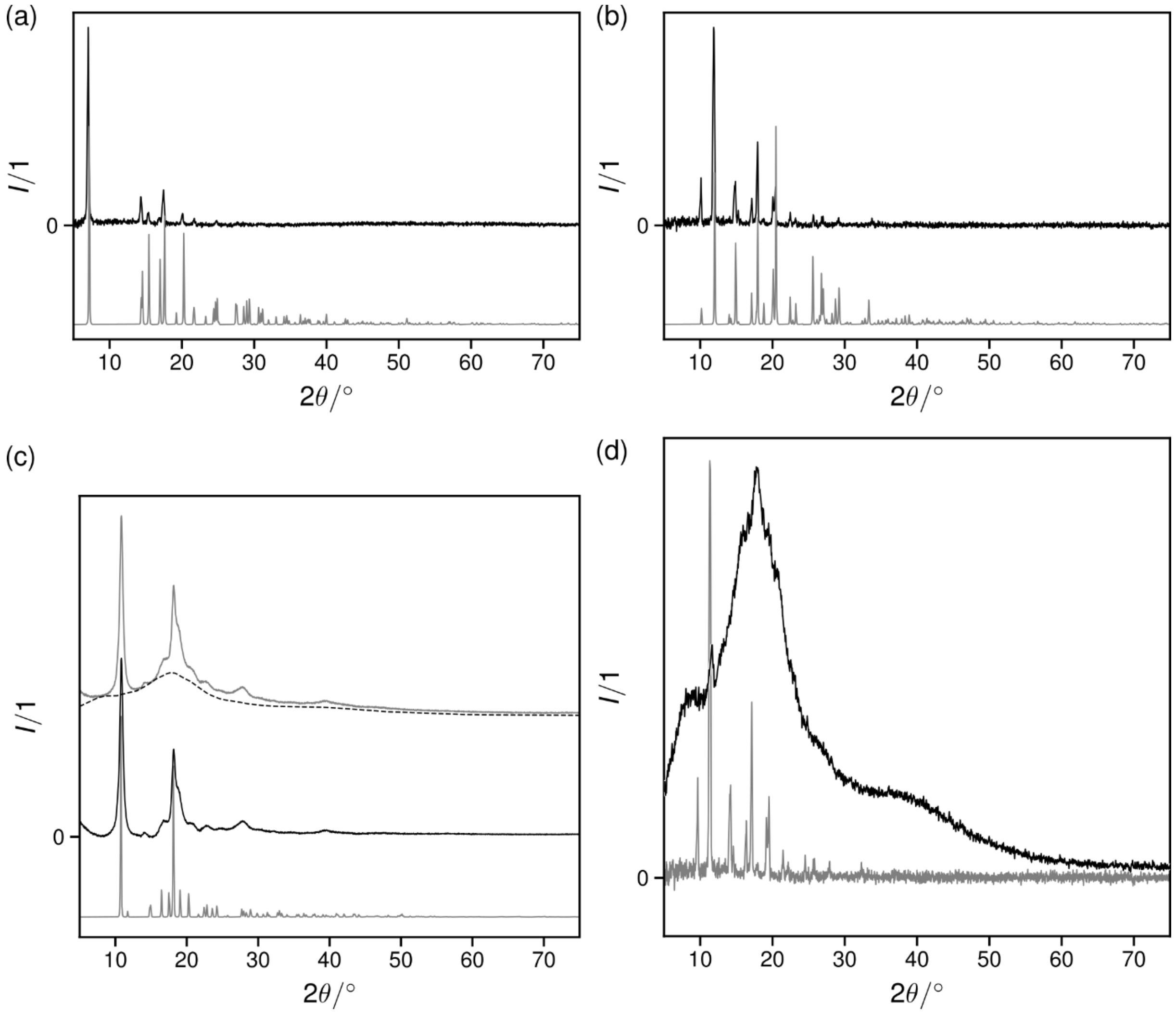

Supplementary Figure 31: Diffraction patterns (Cu Kα) from **5-3S** (a) and **5-3R** (b) and **poly5-3S** (c) S- and **poly5-3R** (d, black lines) with simulated diffractograms from the respective best-match crystal structures (lowest grey lines). In (c), the subtraction of the amorphous phase-diffractogram (dashed line) and the resulting purely crystalline pattern (black full line) are shown. In (d), instead of a best-match crystal pattern, the pattern from residual monomers, (b), is shown.

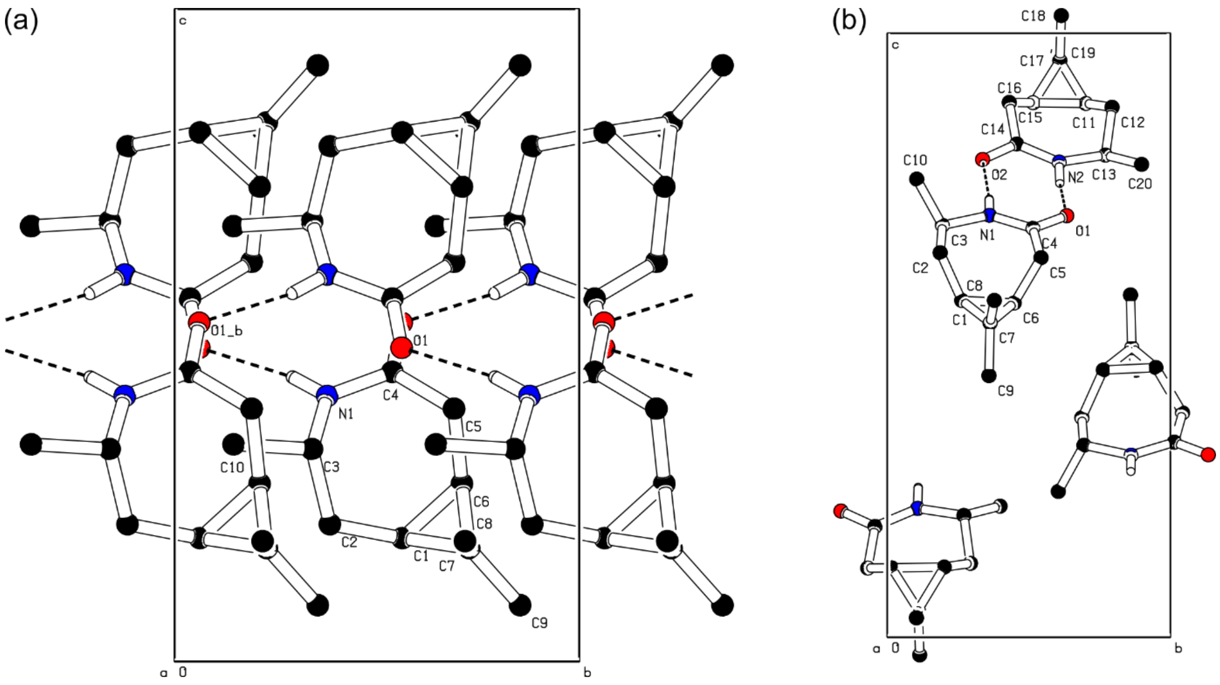

Supplementary Figure 32: Structure of **5-3S** (left) and **5-3R** (right) with bonding hydrogens only, viewed in unit cell direction *a*.

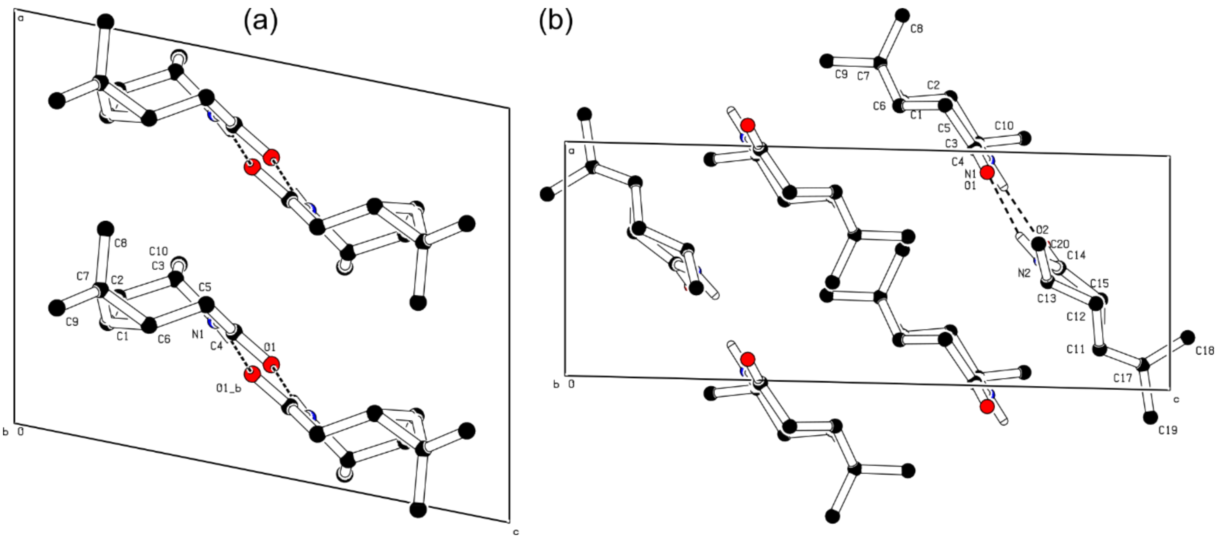

Supplementary Figure 33: Structure of **5-3S** (left) and **5-3R** (right) with bonding hydrogens only, viewed in unit cell direction *b*.

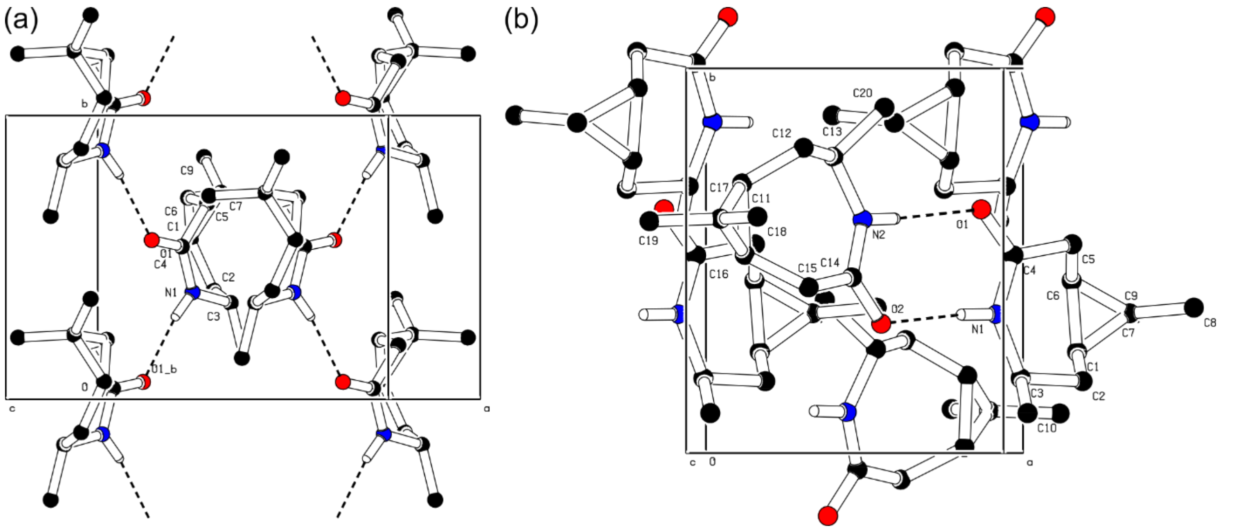

Supplementary Figure 34: Structure of **5-3S** (left) and **5-3R** (right) with bonding hydrogens only, viewed in unit cell direction *c*.

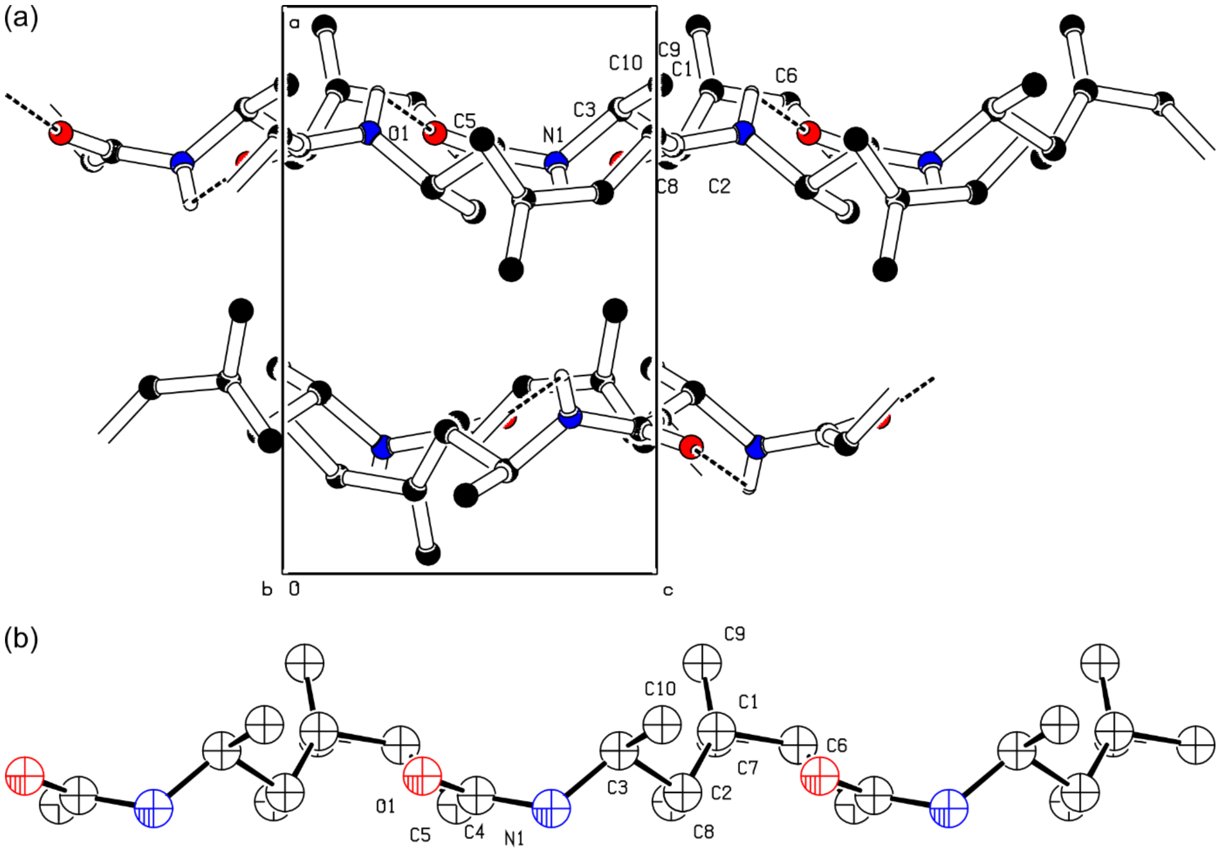

Supplementary Figure 35: Structure of **poly5-3S**, viewed in unit cell direction *b* (unit cell with bonding hydrogens only, the expanded repeating unit without hydrogens (lower).

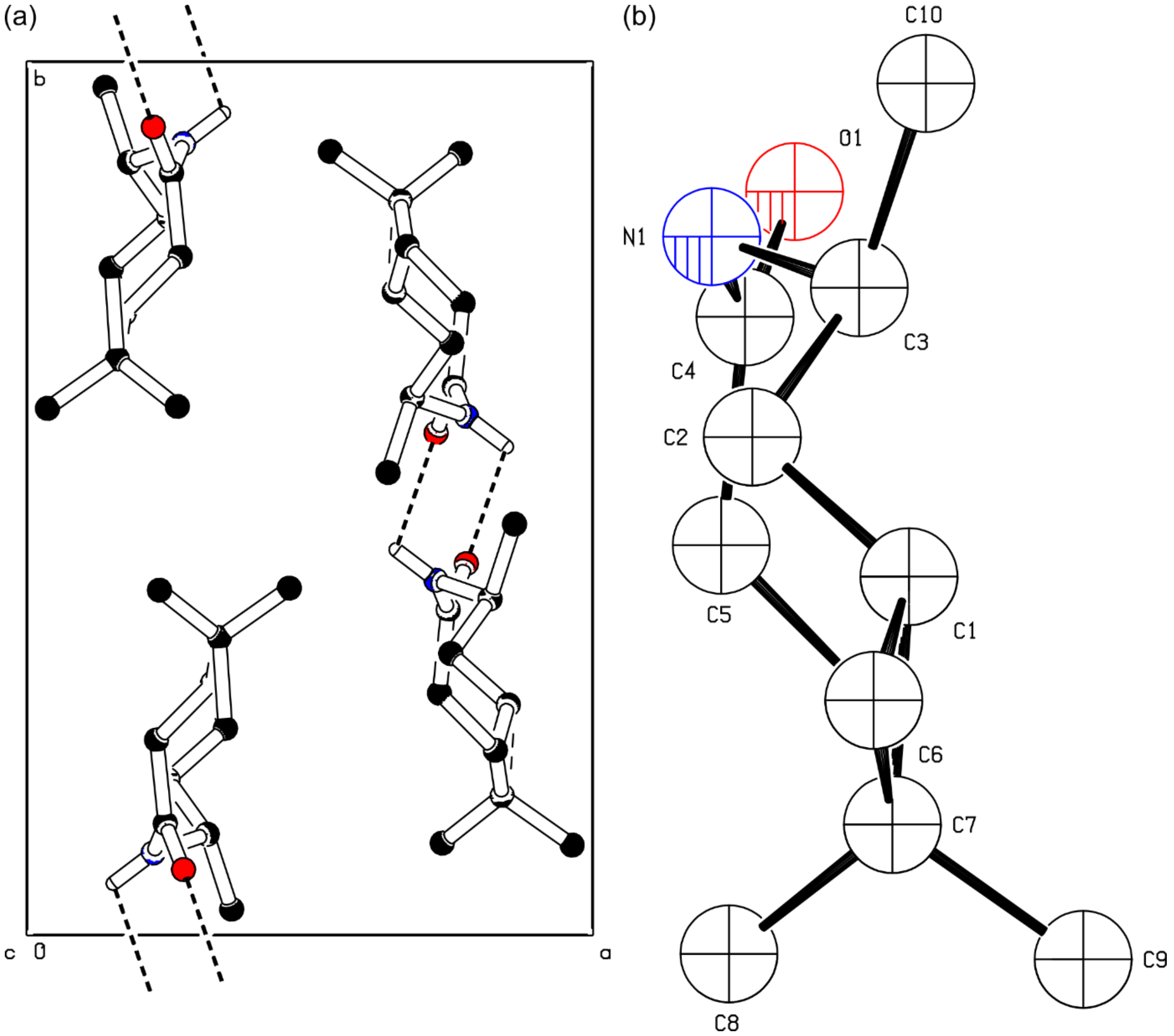

Supplementary Figure 36: Structure of **poly5-3S**, viewed in unit cell direction c; unit cell with bonding hydrogens only (left), the expanded repeating unit without hydrogens (right).

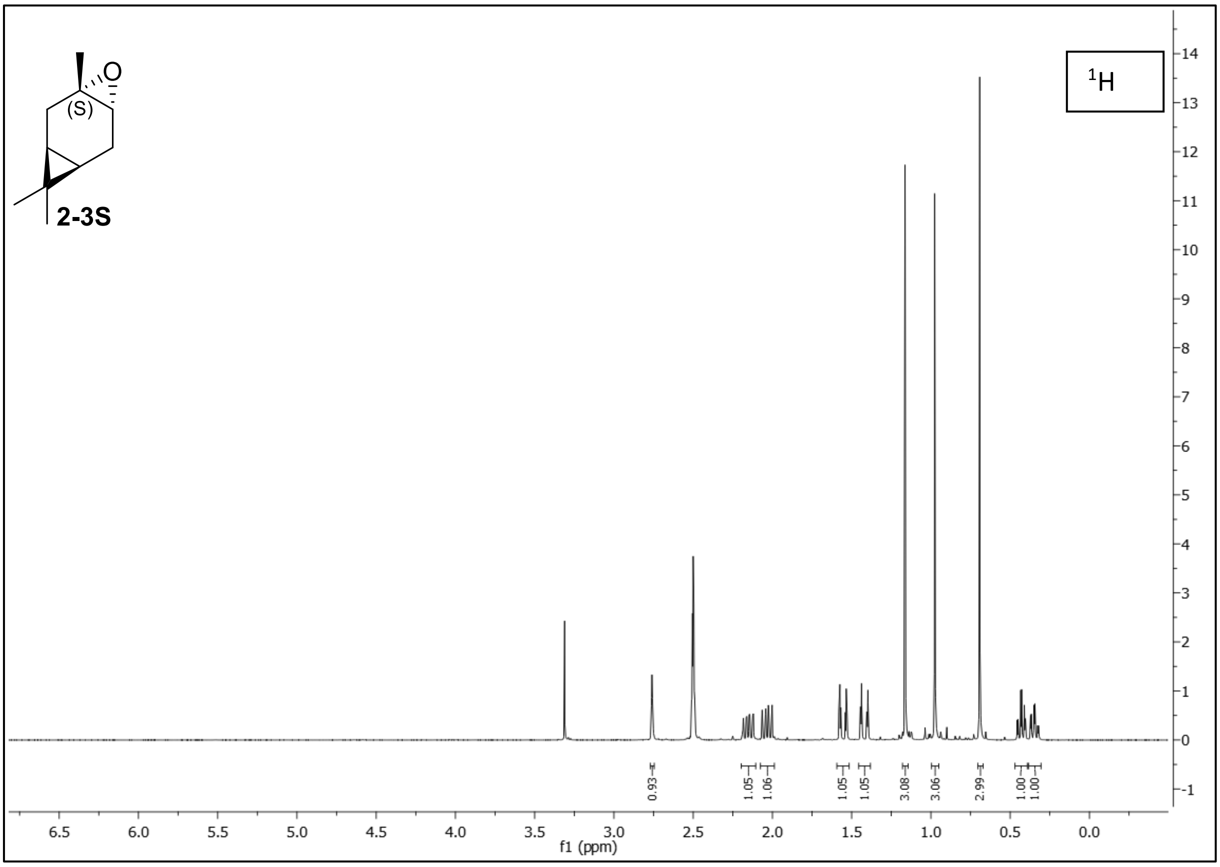

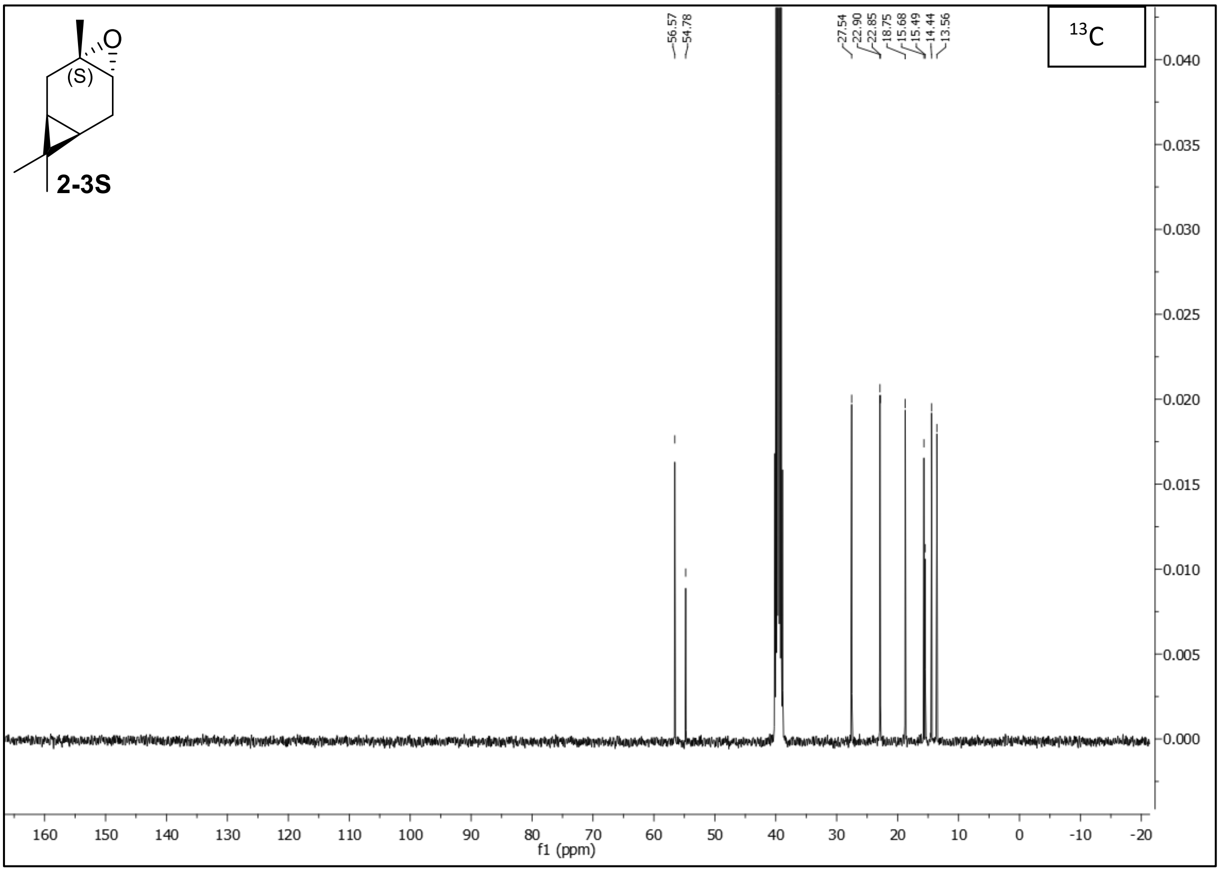

Supplementary Figure 37: NMR spectra of **2-3S**.

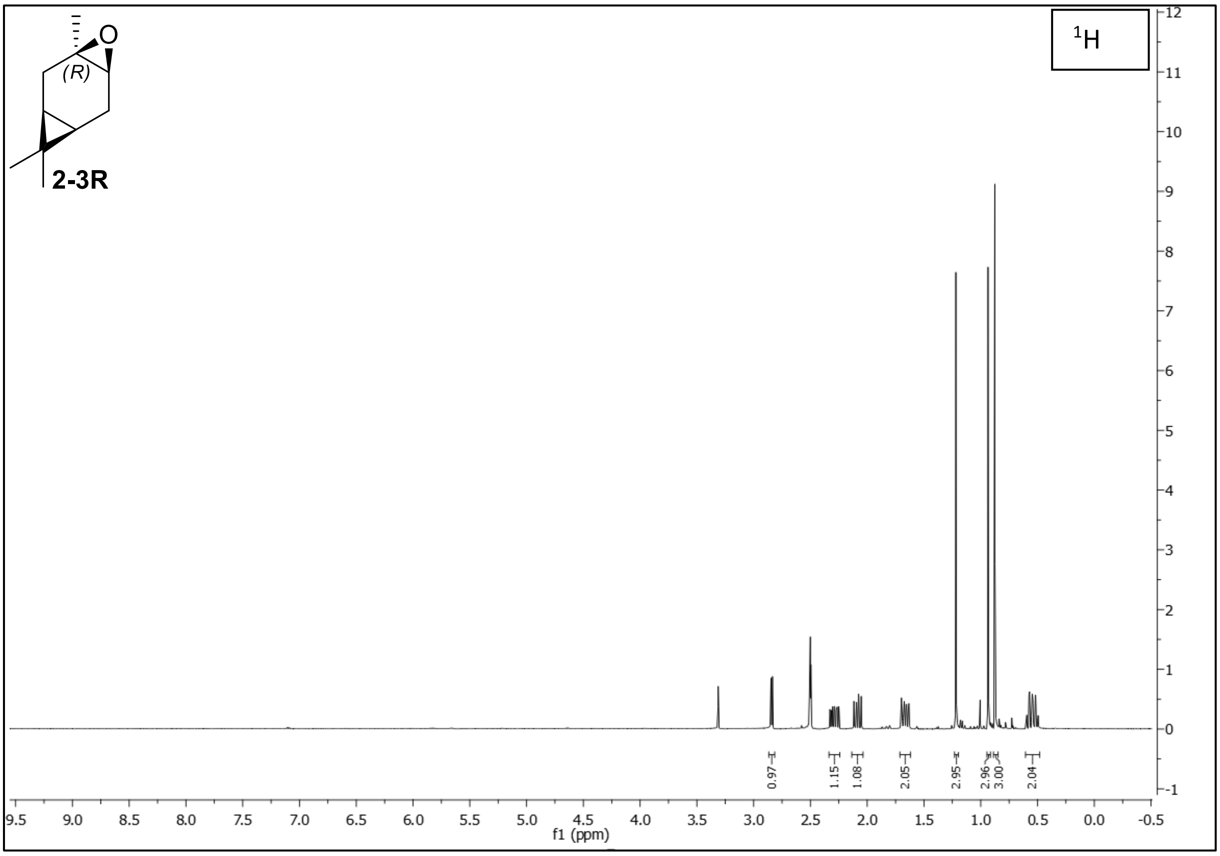

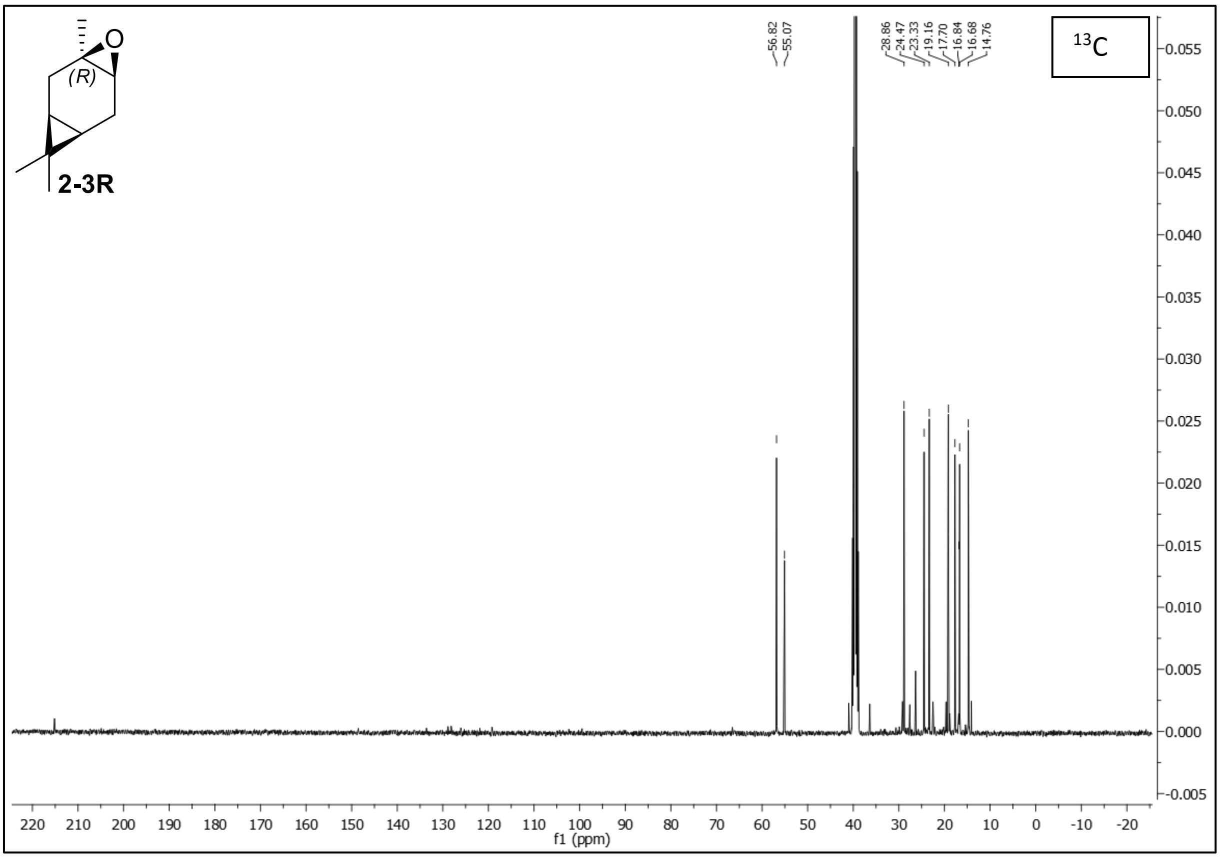

Supplementary Figure 38: NMR spectra of **2-3R**.

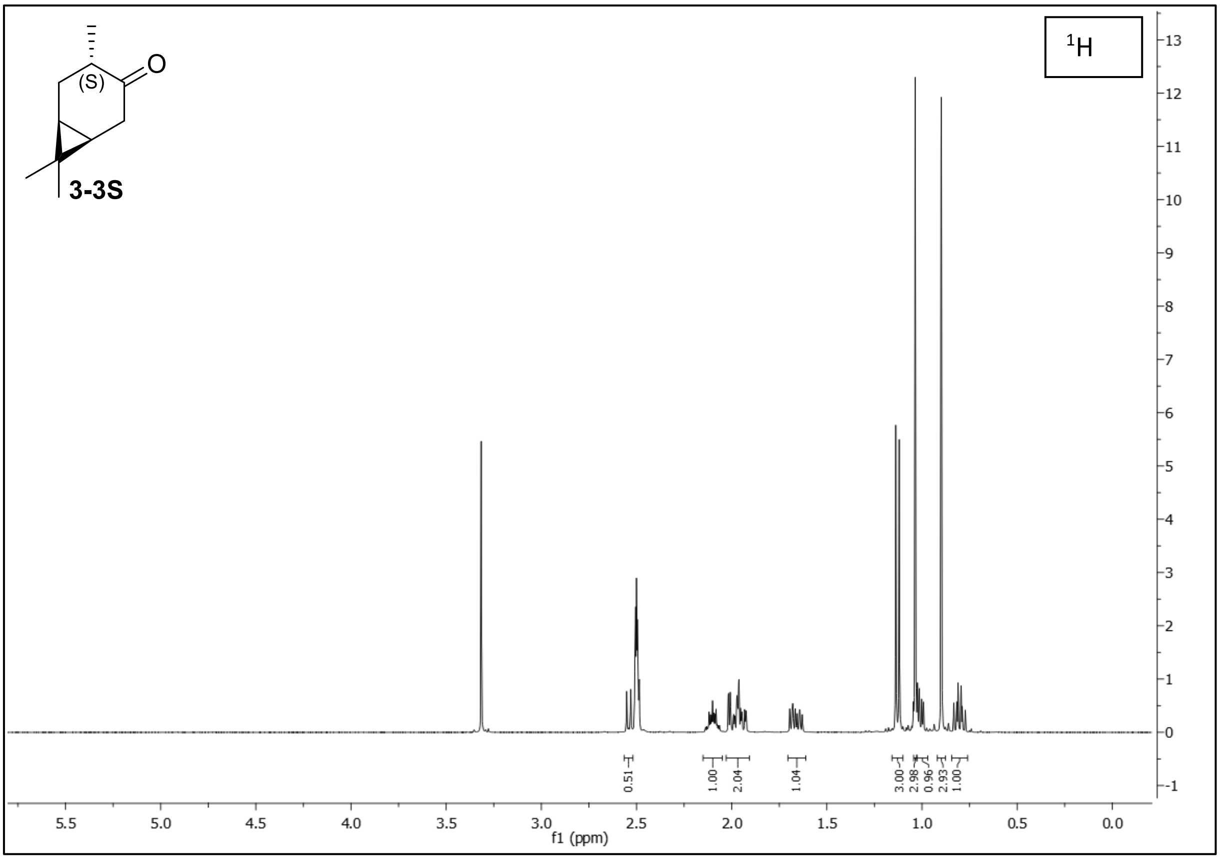

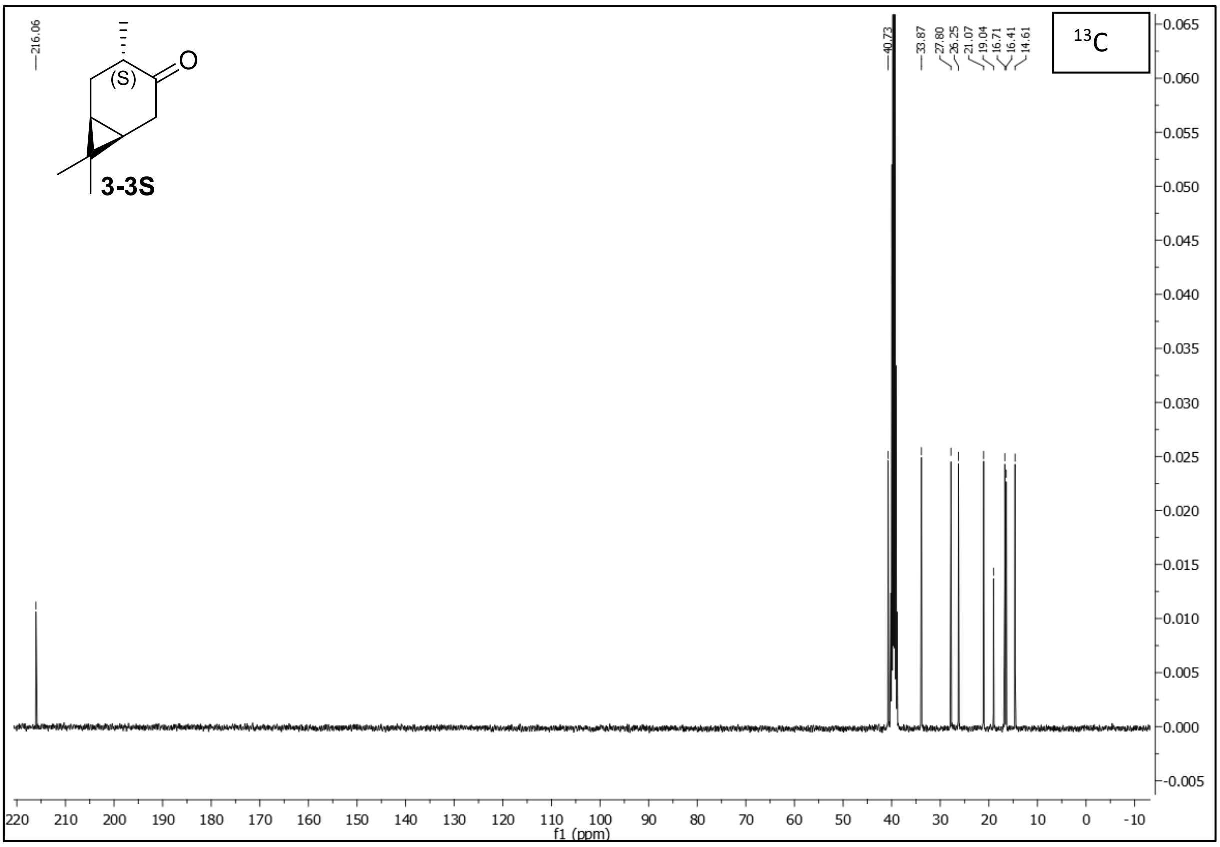

Supplementary Figure 39: NMR spectra of **3-3S**.

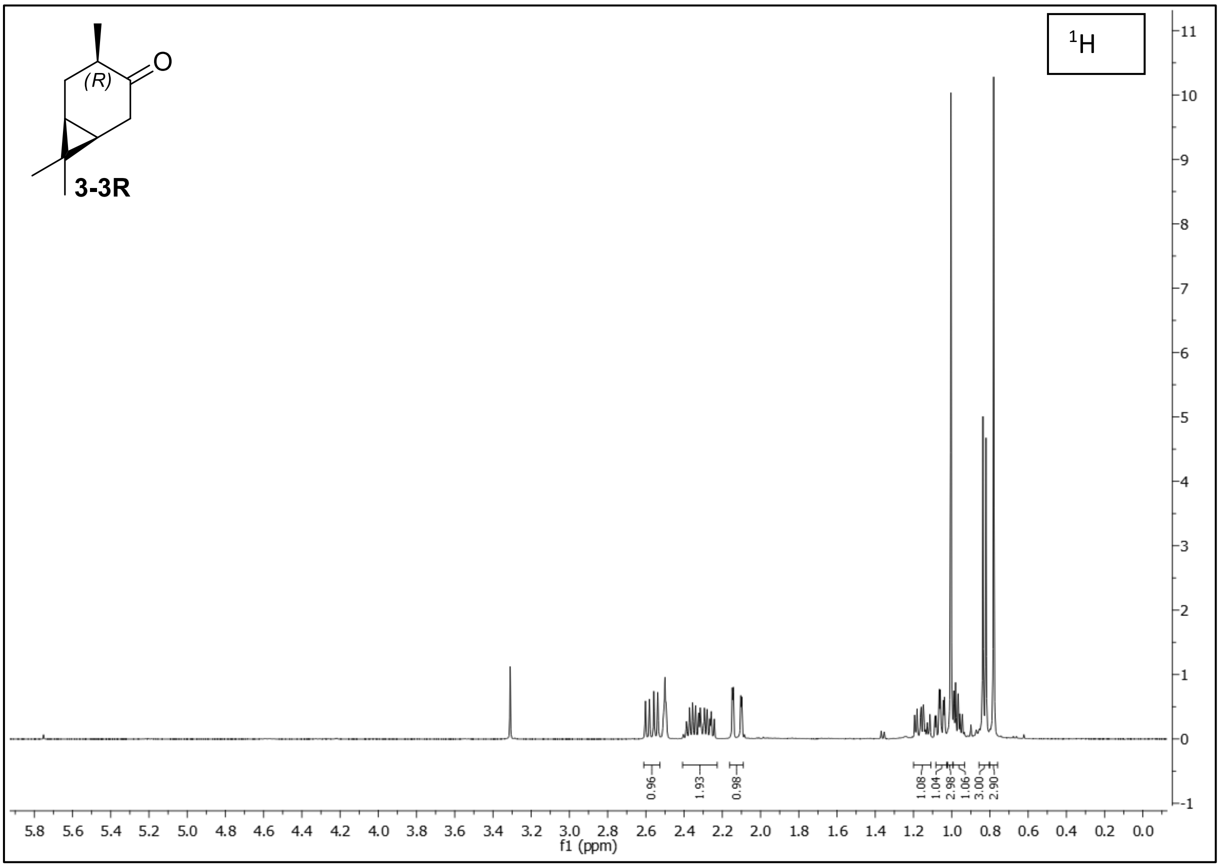

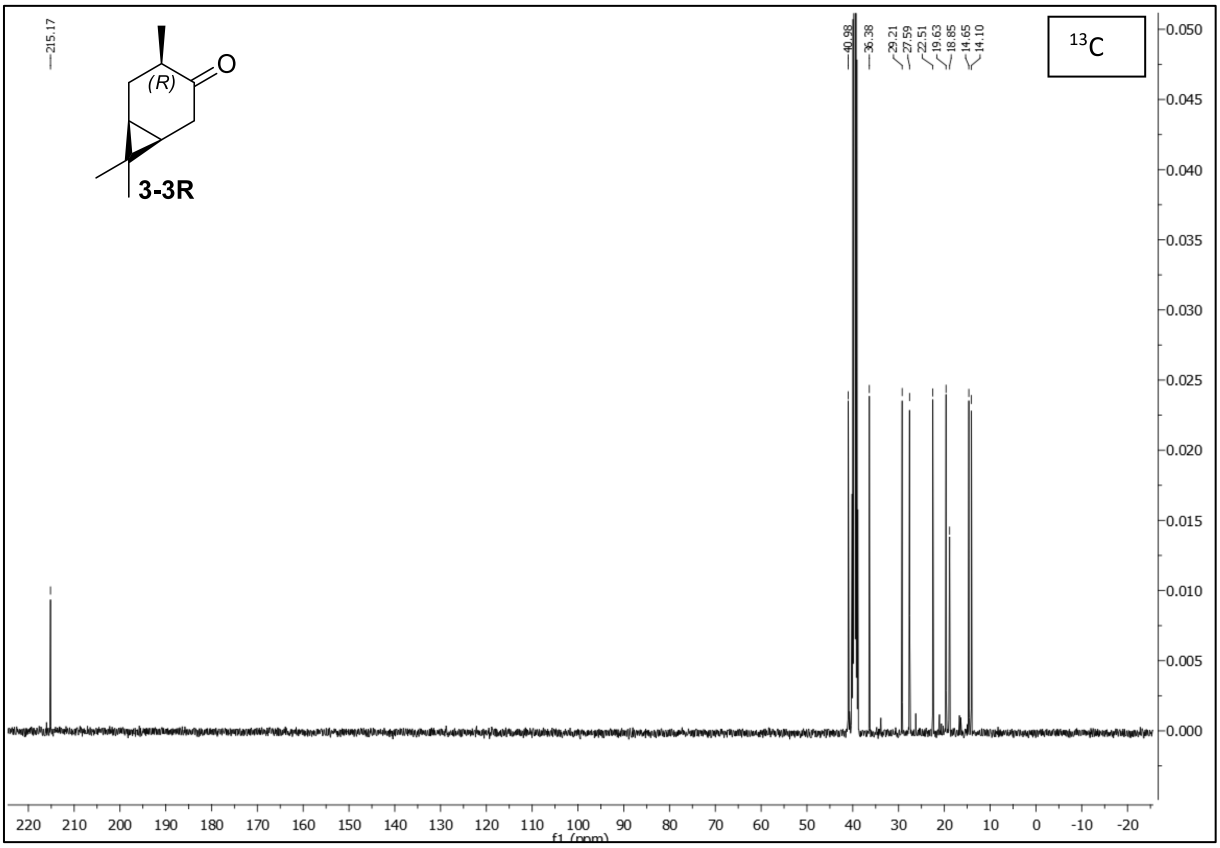

Supplementary Figure 40: NMR spectra of **3-3R**.

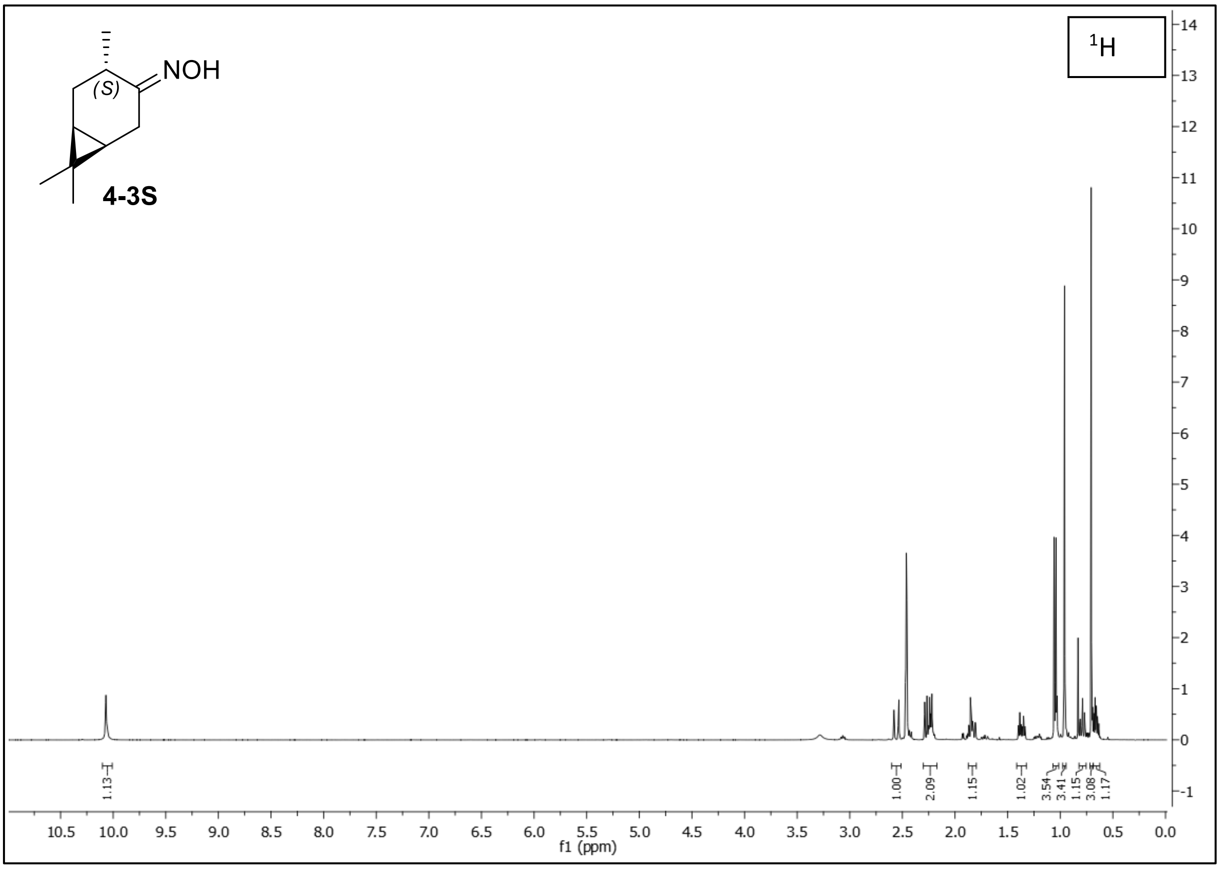

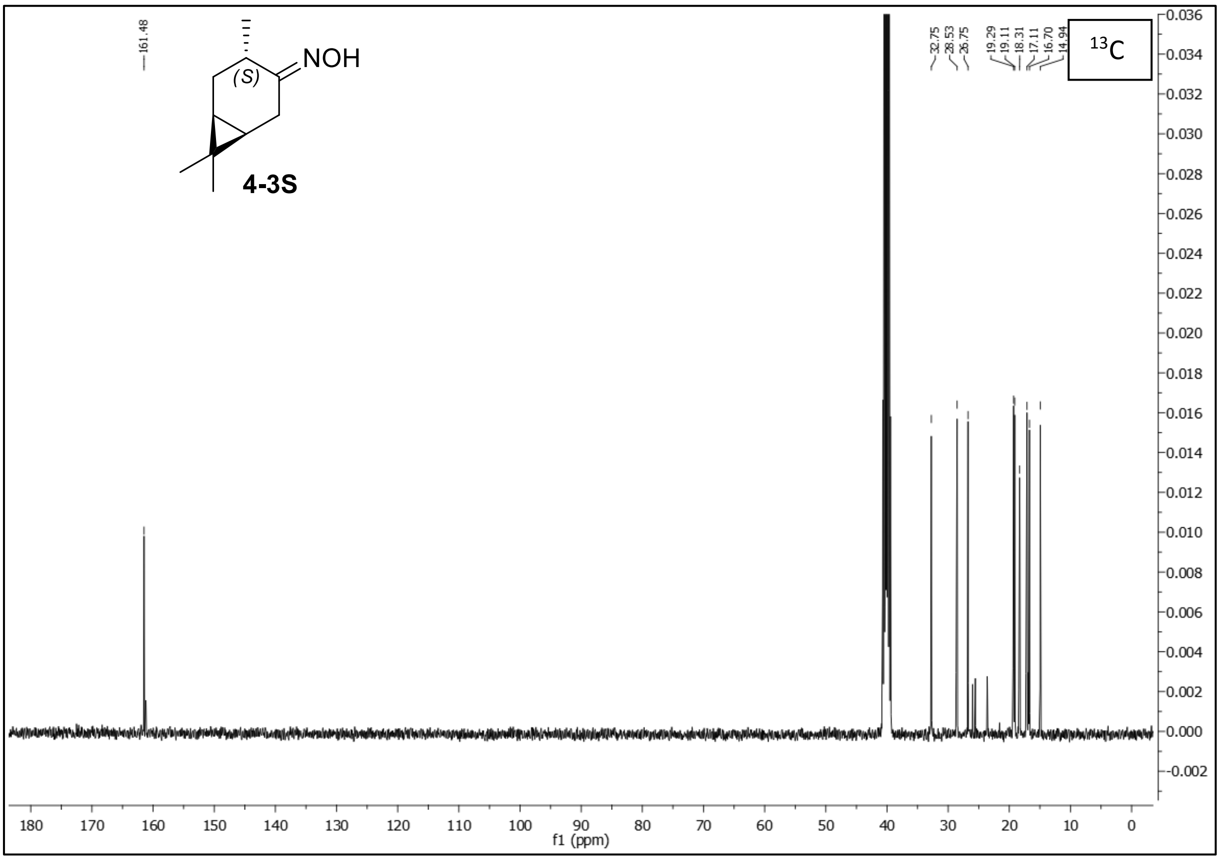

Supplementary Figure 41: NMR spectra of **4-3S**.

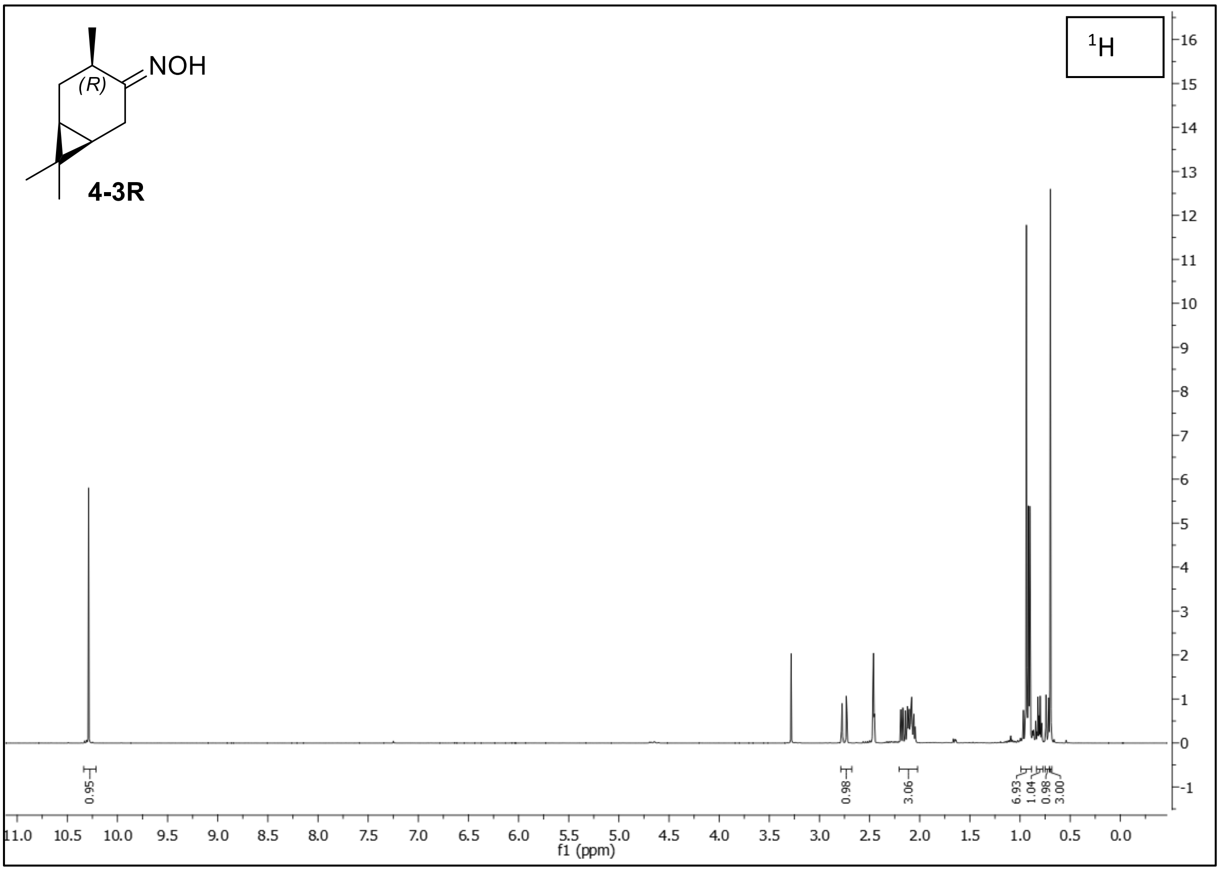

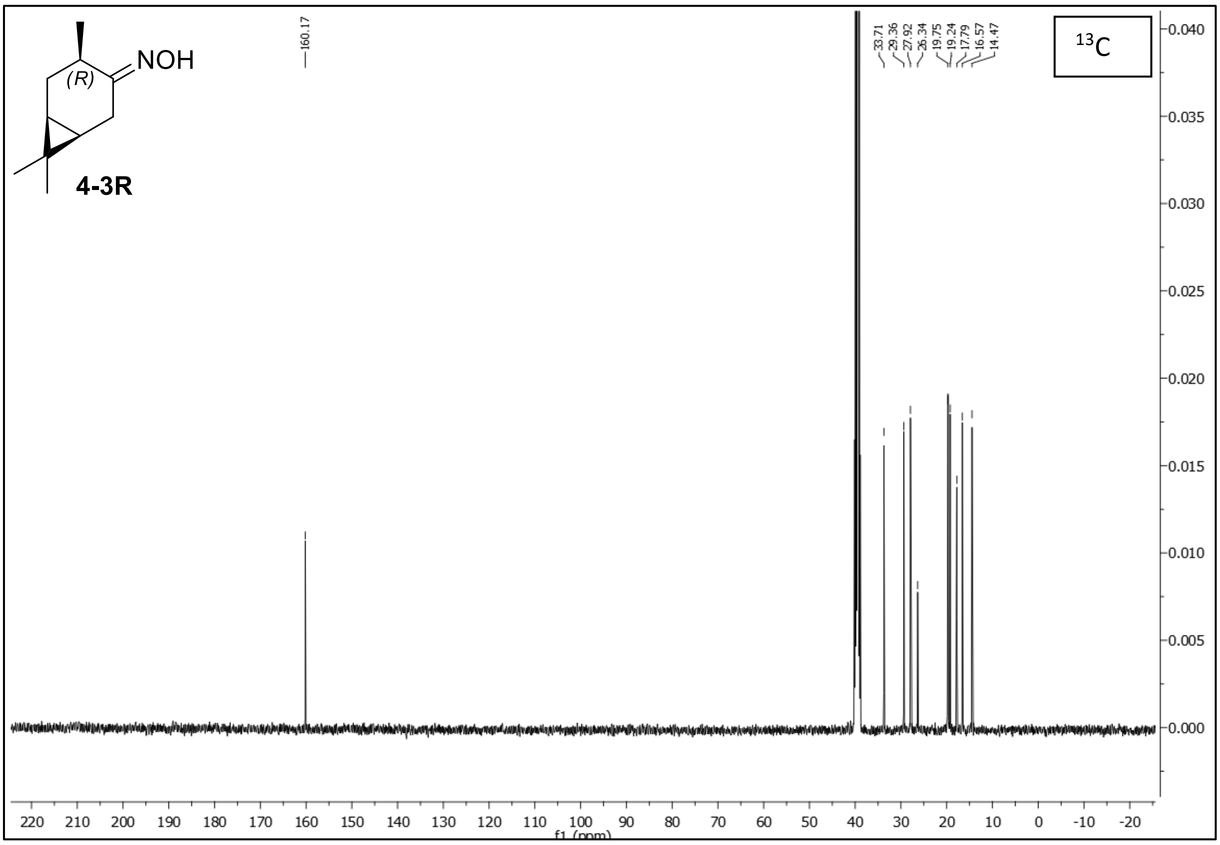

Supplementary Figure 42: NMR spectra of **4-3R**.

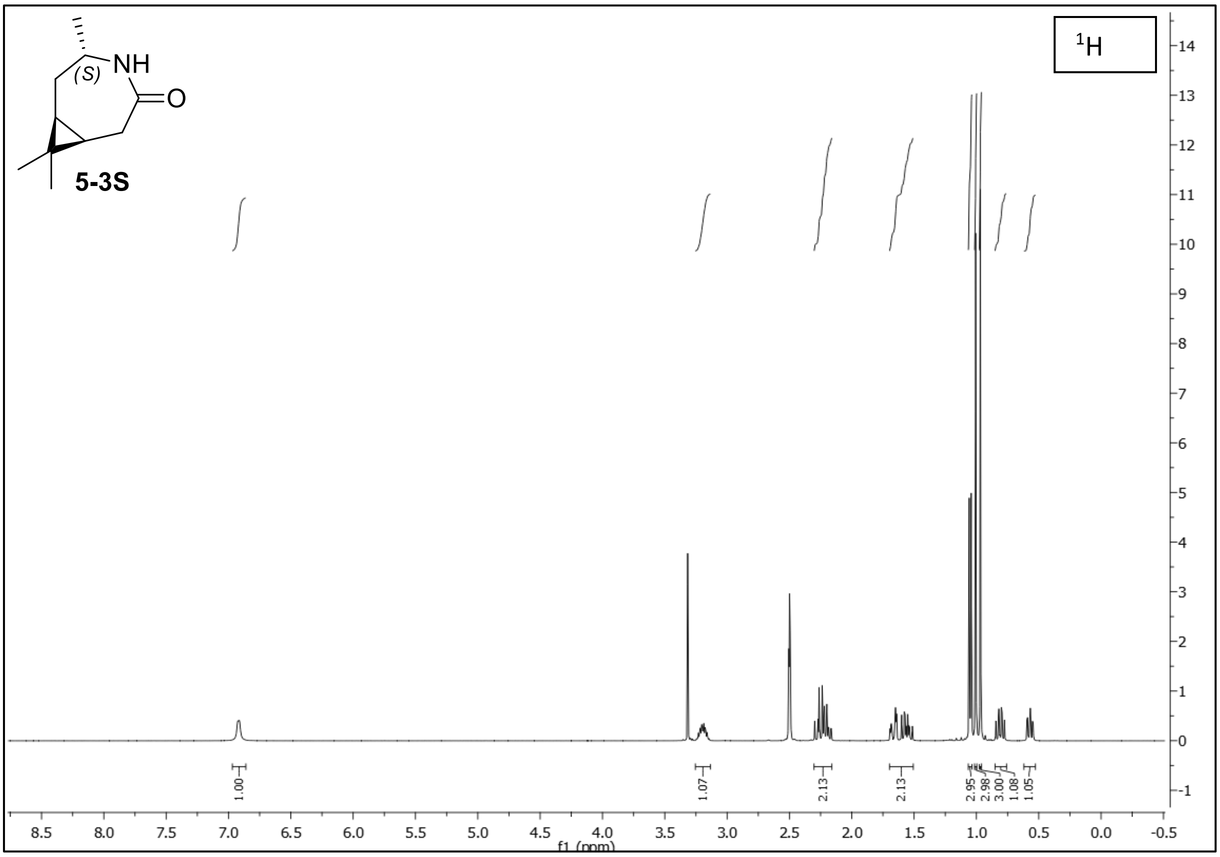

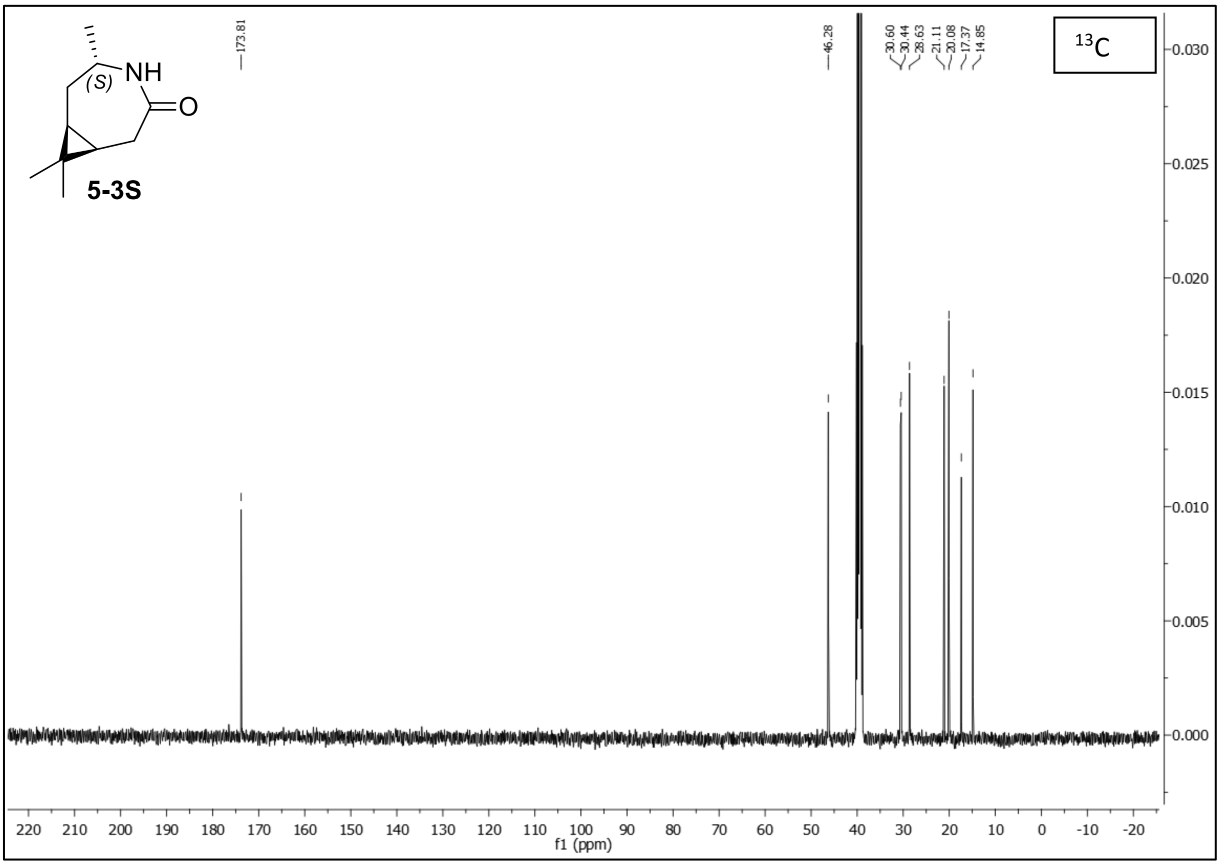

Supplementary Figure 43: NMR spectra of **5-3S**.

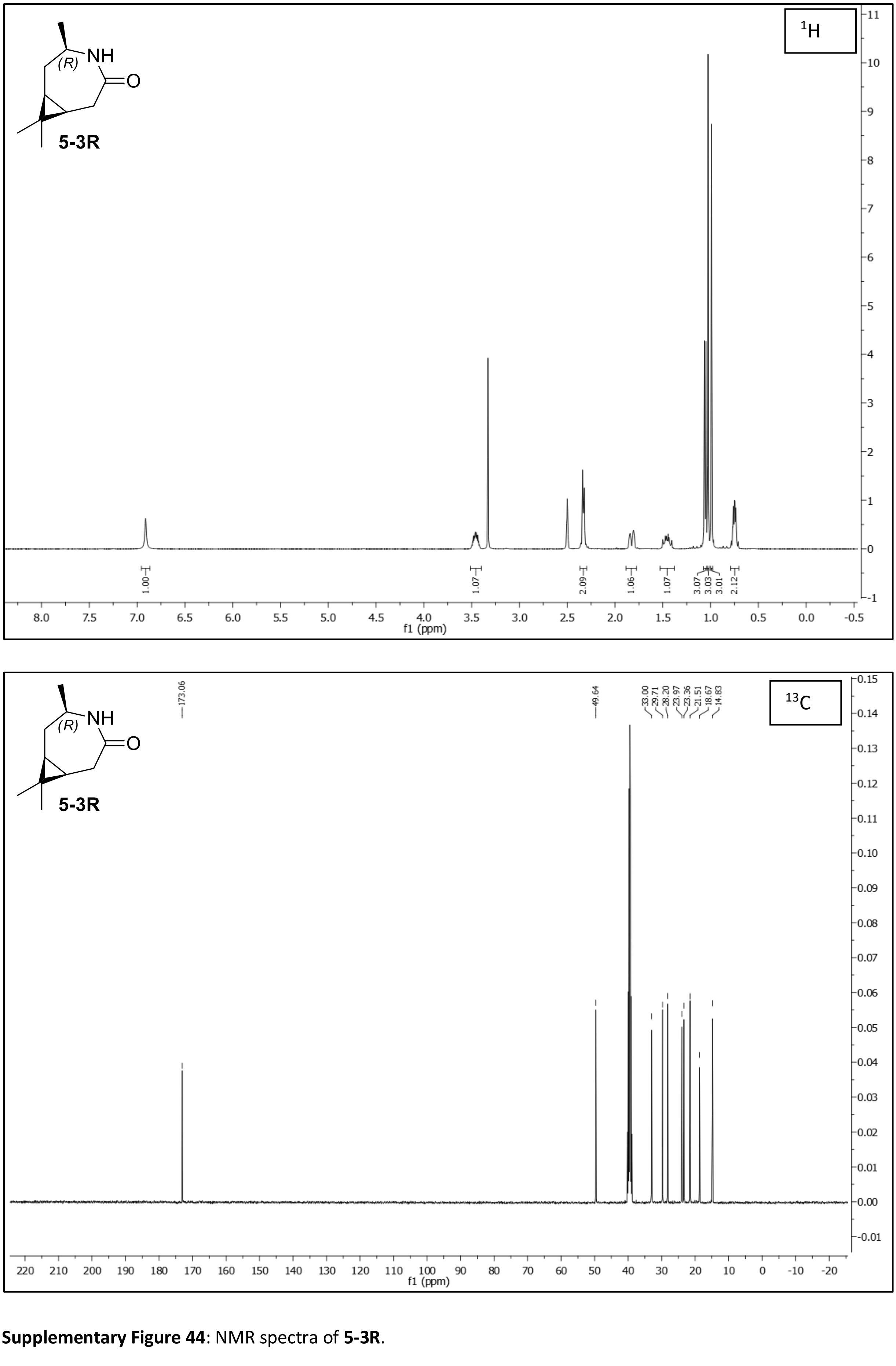

Supplementary Figure 44: NMR spectra of **5-3R**.

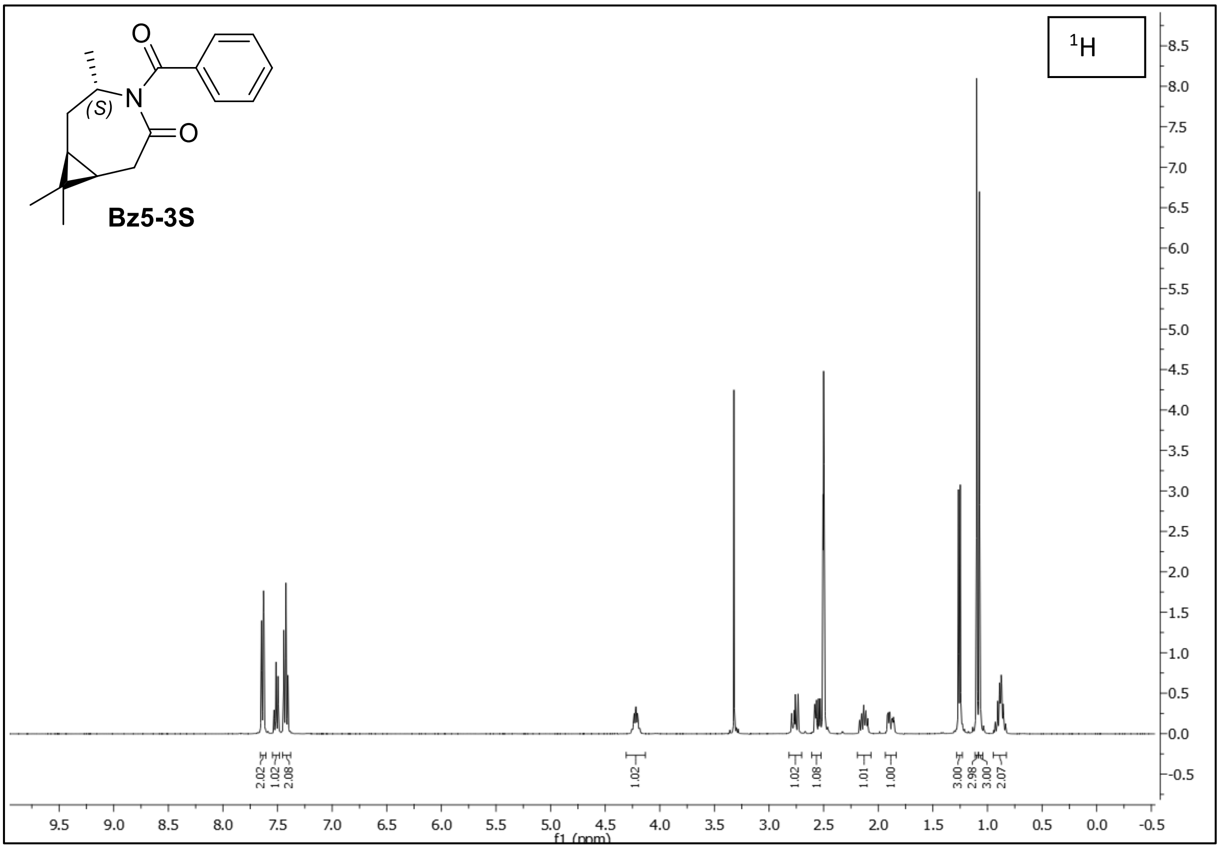

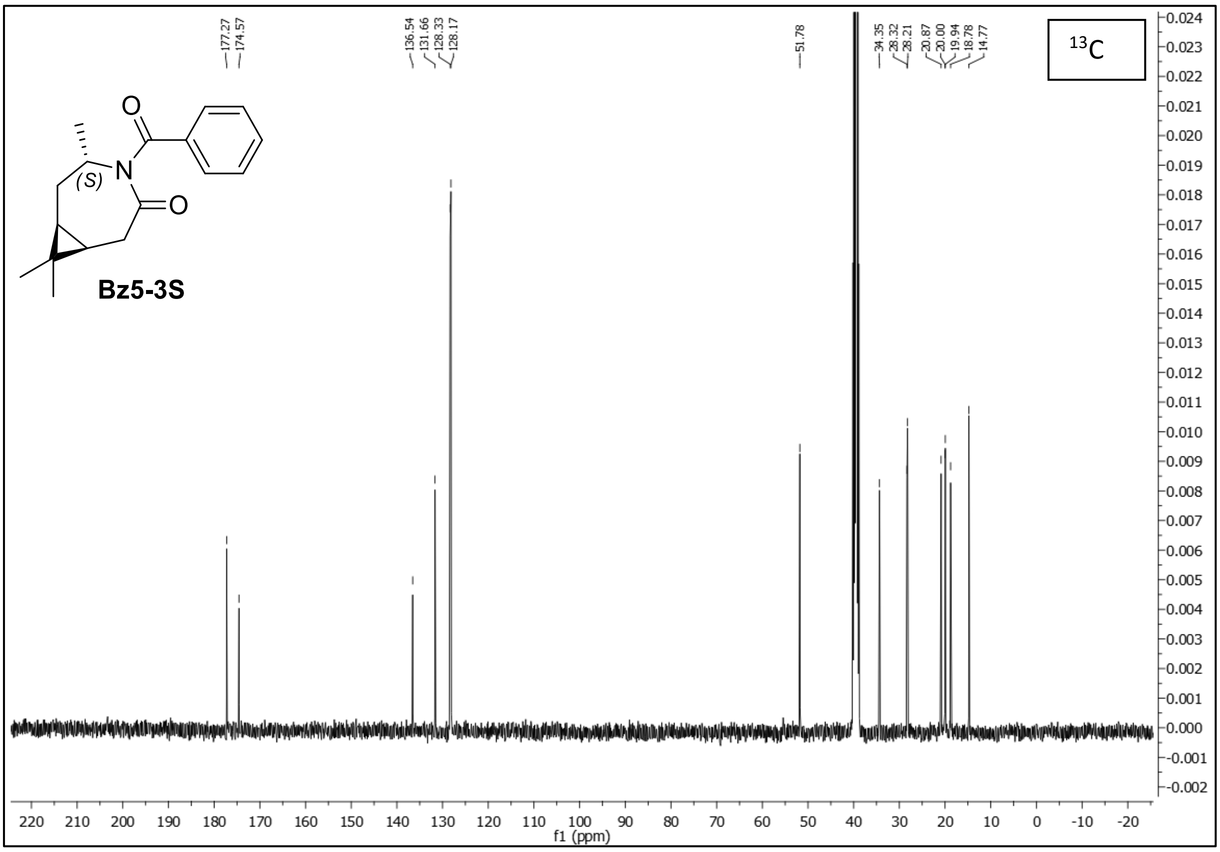

Supplementary Figure 45: NMR spectra of **Bz5-3S**.

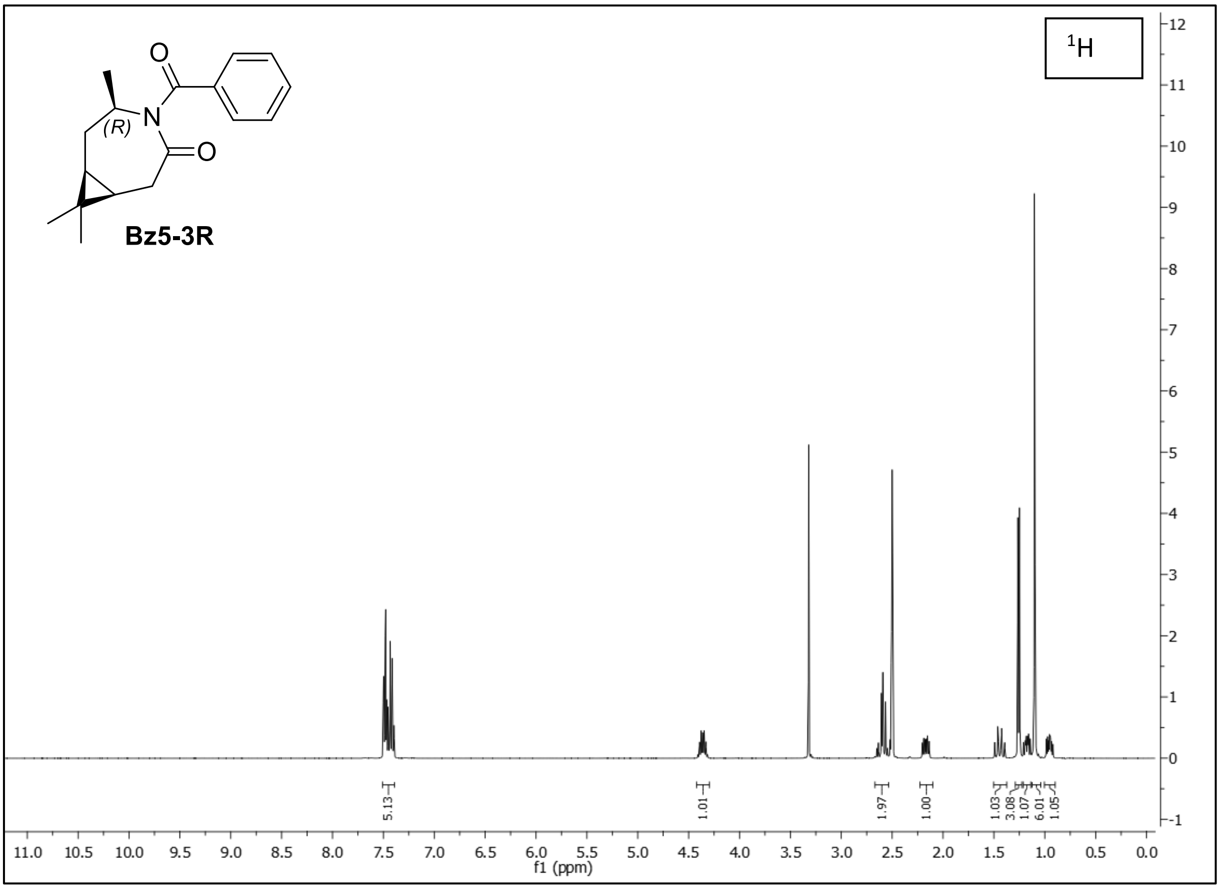

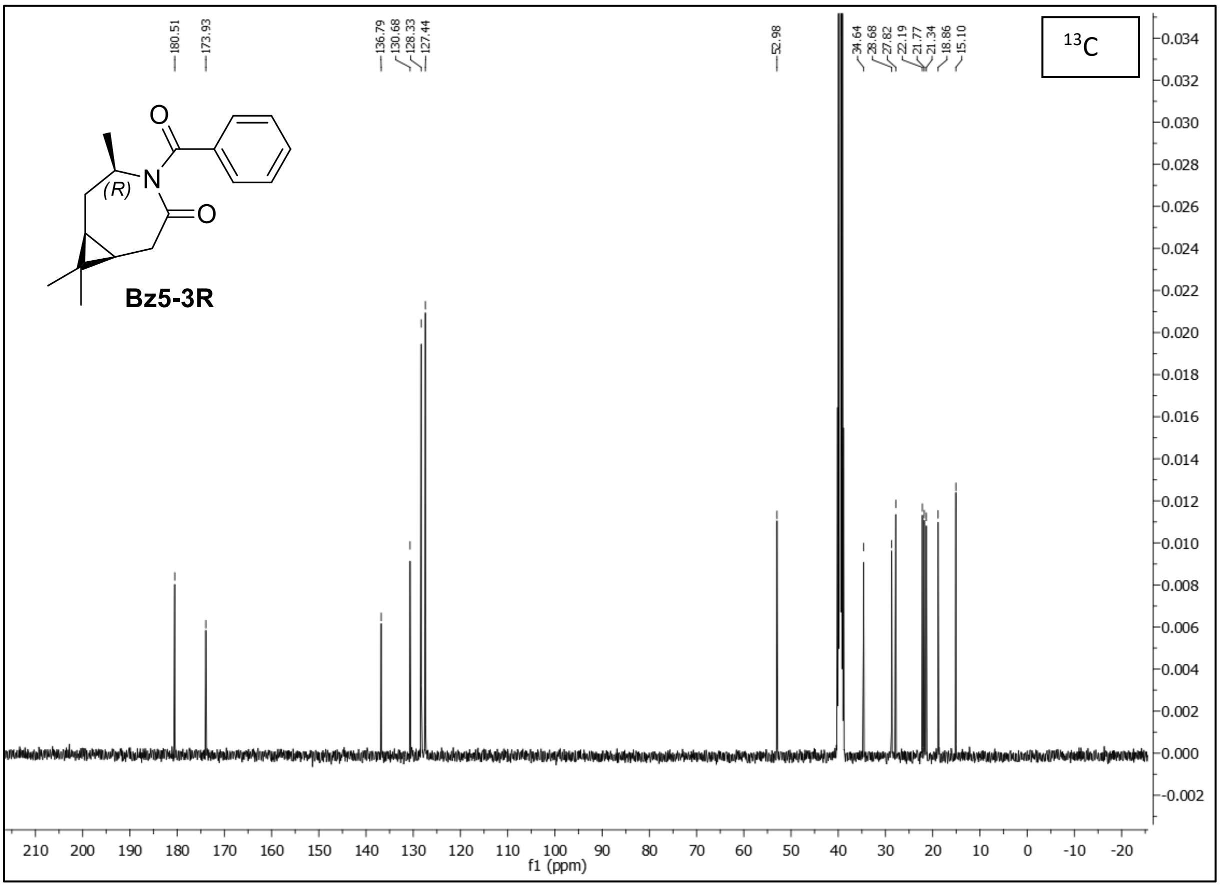

Supplementary Figure 46: NMR spectra of **Bz5-3R**.

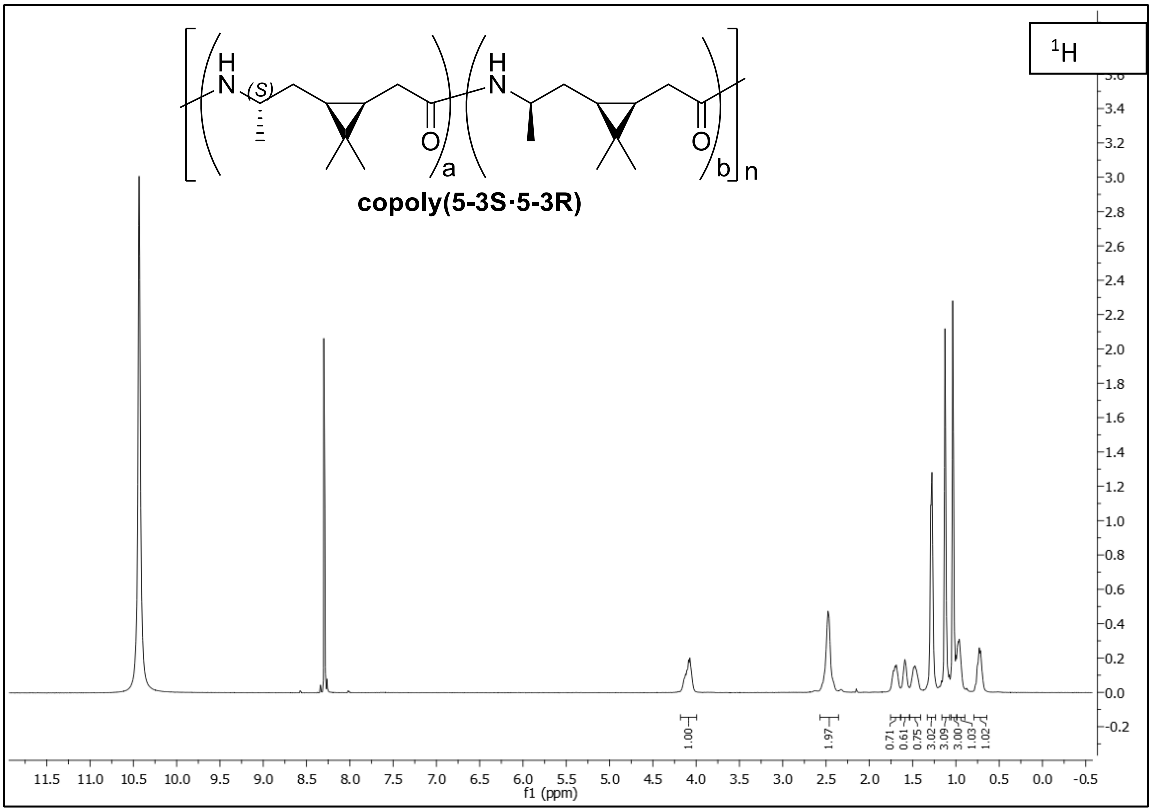

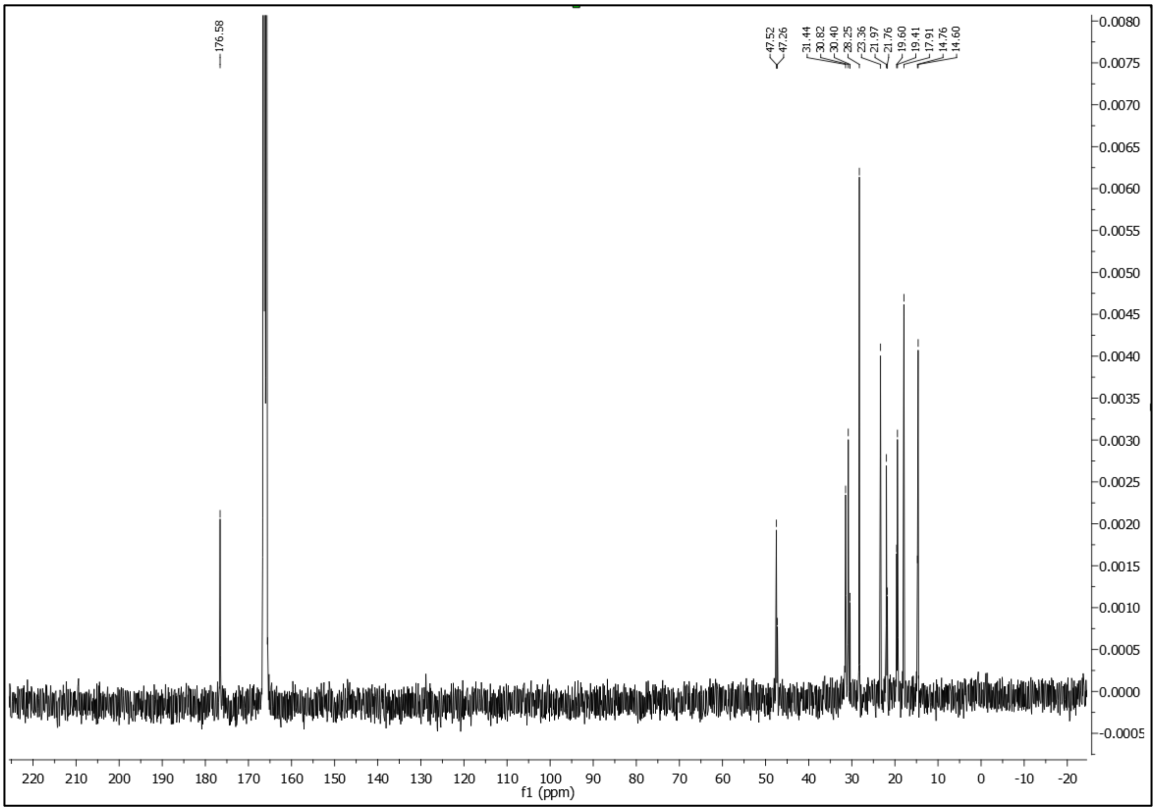

Supplementary Figure 47: NMR Spectra of 3S-caranlactam-3R-caranlactam-copolyamide (DCOOD, 1H 400 MHz, 13C 100 MHz)

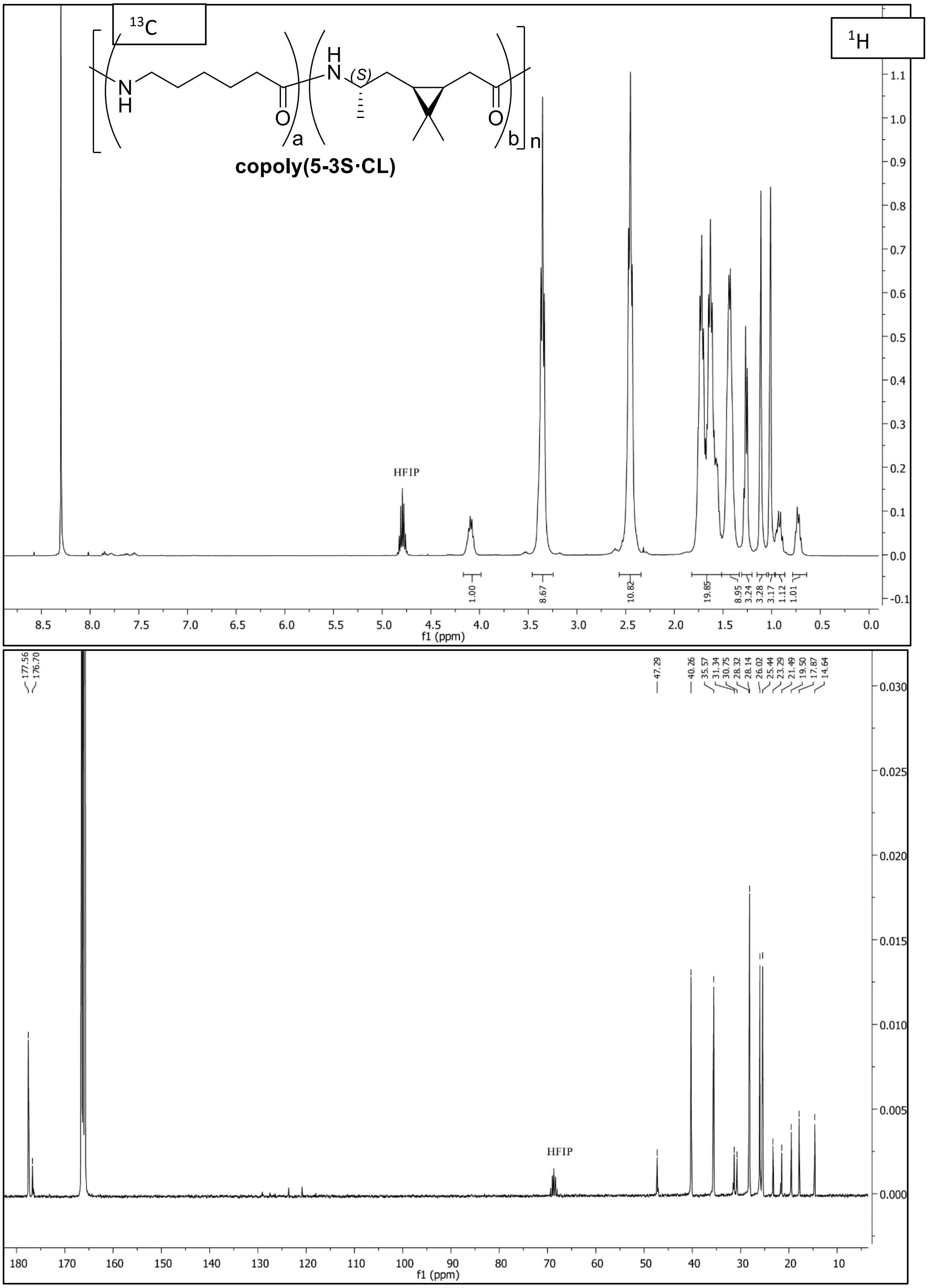

Supplementary Figure 48: NMR Spectra of 3S-caranlactam-caprolactam-copolyamide (DCOOD, ^{1}H 400 MHz, ^{13}C 100 MHz)

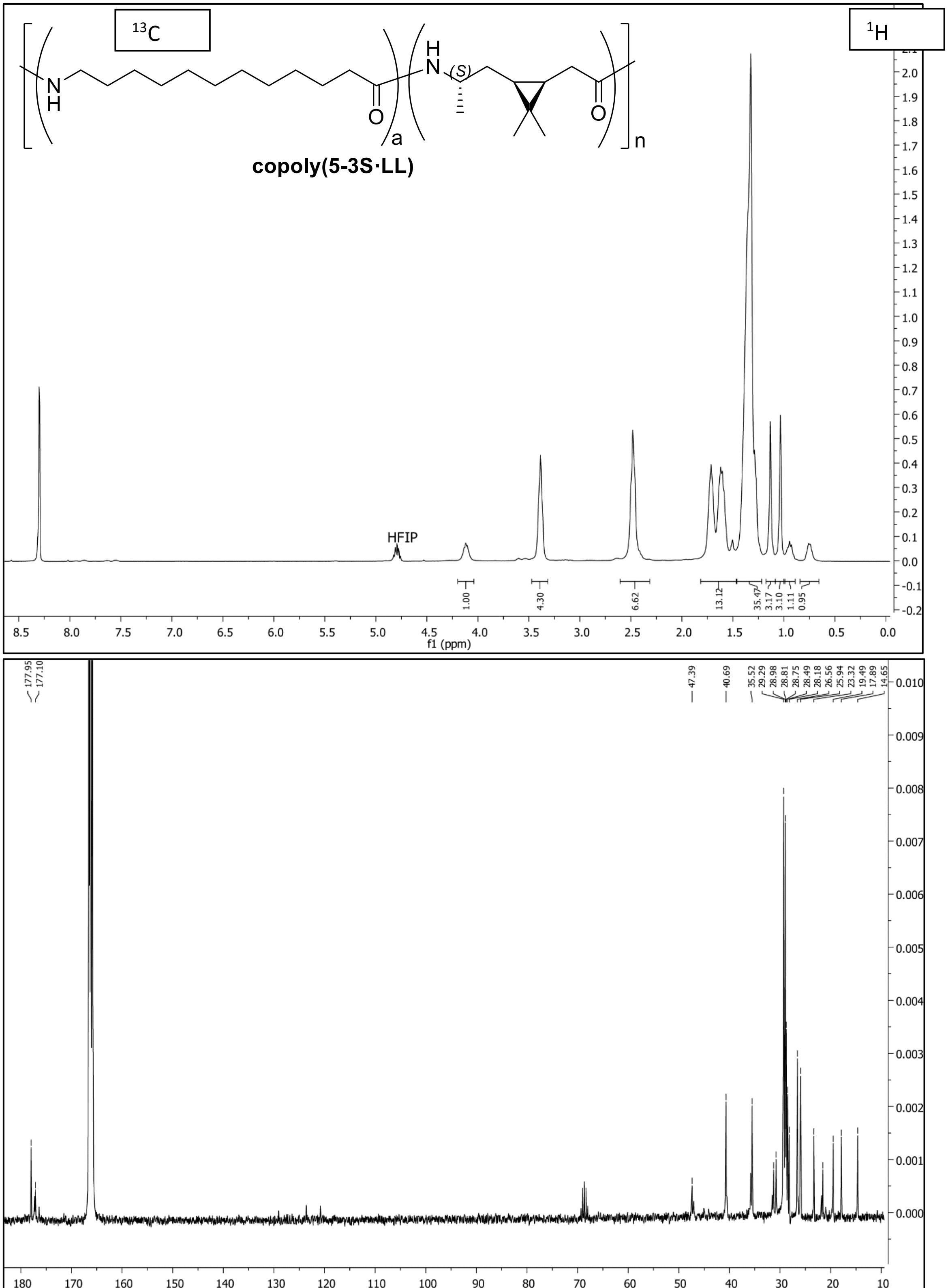

Supplementary Figure 49: NMR Spectra of 3S-caranlactam-laurolactam-copolyamide (DCOOD, ^{1}H 400 MHz, ^{13}C 100 MHz)

2. Supplementary Tables

Supplementary Table 1: Heating- and elution-parameters of the GC-MS analysis and retention times of the (+)-3-carene (**1**) derivatives

**Parameters**	**Values**	
split rate	5	
injection temperature	250 °C	
carrier gas	helium	
column flow-through	1.69 ml/min.	
heating program	50 °C for 1 min.	
	50-120 °C, heating 15 °C per min.	
	120-170 °C, heating 5 °C per min.	
	170-200 °C, heating 20 °C per min.	
	200 °C for 7 min.	
column	BPX5 (CS Chromatography), length 30 m,	
	inner diameter 0.25 µm, diameter 0.25 mm	
**Substance**	**Retention time [min]**	
(+)-3-carene (**1**)	5.7	-
3S-caranepoxide (**2-3S**)	7.3	-
3S-caranketone (**3-3S**)	8.2	-
3R-caranketone (**3-3R**)	8.0	-
3R-caranoxime (**4-3R**)	11.1 (trans, major)	11.0 (cis, minor)
3S-caranoxime (**4-3S**)	10.8 (trans, major)	10.9 (cis, minor)
3R-caranepoxide (**2-3R**)	7.2	-
3S-caranlactam (**5-3S**)	14.6 (major)	14.5 (minor)
3R-caranlactam (**5-3R**)	14.1 (major)	14.3 (minor)

Supplementary Table 2: Influence of solvent polarity on the rearrangement of 3S-caranepoxide (**2-3S**) to the R- and S-caranketone isomers. All experiments were carried out with a concentration of 1 M at 25 °C and 0.2% $Fe(ClO_4)_2 \cdot H_2O$ for 8 h. Conversion of 3S-caranepoxide (**2-3S**) was 100%. Data values refer to TIC of GCMS analytics (uncorrected values).

**Entry**	**Solvent**	**Relative polarity***	**Σ ketone [%]**	**3-3R [%]**	**3-3S [%]**
**1**	Cyclohexane	0.006	70	11	89
**2**	Hexane	0.009	64	13	87
**3**	Heptane	0.012	64	13	87
**4**	Toluene	0.099	71	14	86
**5**	2-Me-THF	-	66	26	74
**6**	THF	0.207	51	39	61
**7**	EtOAc	0.228	67	44	56
**8**	Chloroform	0.259	68	38	72
**9**	DCM	0.309	60	57	43
**10**	2-butanone	0.327	38	60	40
**11**	Acetone	0.355	24	61	39
**12**	Isopropanol	0.546	12	20	80
**13**	EtOH	0.654	26	50	50
**14**	MeOH	0.762	<1	-	-

* data retrieved from Christian Reichardt, Solvents and Solvent Effects in Organic Chemistry, Wiley-VCH Publishers, 3rd ed., 2003.

Supplementary Table 3: Screening of Lewis-acidic catalysts under various conditions. Data values refer to TIC-area of GCMS spectra (uncorrected values).

Entry	Catalyst [%]	Solvent	C [M]	T [°C]	Conversion [%]	Σ ketone [%]	3-3R [%]	3-3S [%]	t [h]
**1**	$ZnBr_2$ 10%	-	-	60	100	60	25	75	4
**2**	$Fe(OTf)_2$ 10%	EtOAc	0.05	60	100	57	30	70	1
**3**	ZSM-5 15 w%	EtOAc	1	45	100	54	34	66	1
**4**	$Zn(OTf)_2$ 10%	EtOAc	0.05	60	100	60	34	66	1
**5**	$ZnBr_2$ 100%	EtOAc	0.25	70	100	68	45	55	2
**6**	$ZnBr_2$ 10%	toluene	0.25	80	100	64	45	55	4
**7**	$ZnBr_2$ 10%	$CHCl_3$	0.25	80	100	66	45	55	4
**8**	$AlCl_3$ 100%	EtOAc	0.25	60	45	20	50	50	48
**9**	$FeCl_3 \cdot 6H_2O$ 100%	EtOAc	0.25	60	100	22	50	50	12
**10**	$ZnBr_2$ 100%	EtOAc	0.25	60	100	85	55	45	12
**11**	$Ti(BuO)_4$ 20%	EtOAc	0.2	50	0	0	0	0	12
**12**	$Sn(OAc)_2$ 10%	EtOAc	0.25	50	0	0	0	0	12
**13**	$CuCl_2$ 100%	EtOAc	0.25	60	0	0	0	0	12
**14**	$CeNO3 \cdot 6H_2O$ 100%	EtOAc	0.25	60	0	0	0	0	12
**15**	$ZnSO_4 \cdot 7H_2O$ 100%	EtOAc	0.25	60	0	0	0	0	12
**16**	$ZnCl_2$ 100%	EtOAc	0.25	60	0	0	0	0	12
**17**	$CoCl_2 \cdot 6H_2O$ 100%	EtOAc	0.25	60	0	0	0	0	12
**18**	CuI 100%	EtOAc	0.25	60	0	0	0	0	12
**19**	FeOAc 100%	EtOAc	0.25	60	0	0	0	0	12

Supplementary Table 4: Comparison of Fe- and Zn-Lewis acids at 60 °C in cyclohexane with a concentration of 1 M and 0.2% catalyst. Data values refer to TIC-area of GCMS spectra (uncorrected values).

**Entry**	**Catalyst [%]**	**Conversion [%]**	**Σ ketone [%]**	**3-3R [%]**	**3-3S [%]**	**t [h]**
**1**	$Zn(OAc)_2$	0	0	0	0	12
**2**	$Zn(OTf)_2$	48	70	9	91	40
**3**	$Zn(ClO_4)_2 \cdot H_2O$	73	78	13	77	4
**4**	$Fe(OAc)_2$	0	0	0	0	0
**5**	$Fe(OTf)_2$	91	73	15	85	0.5
**6**	$Fe(ClO_4)_2 \cdot H_2O$	100	82	15	85	0.5
**7**	$Fe(ClO_4)_3 \cdot H_2O$	100	81	16	84	0.5

Supplementary Table 5: Meinwald rearrangement of 3S-caranepoxide (**2-3S**) to ketones **3-3S** and **3-3R** using sulfonic acids. Data values refer to TIC-area of GCMS spectra (uncorrected values).

**Entry**	**Catalyst [%]**	**Solvent**	**C [M]**	**T [°C]**	**Conversion [%]**	**Σ ketone [%]**	**3-3R [%]**	**3-3S [%]**	**t [h]**
**1**	CF_3SO_3H 0.1%	toluene	1	25	100	73	13	87	1
**2**	CF_3SO_3H 0.1%	pentane	0.5	25	100	68	13	87	1
**3**	CF_3SO_3H 0.1%	hexane	0.5	50	100	67	15	85	1
**4**	CF_3SO_3H 0.1%	toluene	0.5	50	100	74	15	85	1
**5**	PTSA 1%	cyclohexane	1	60	100	69	17	83	12
**6**	CF_3SO_3H 0.1%	toluene	1	60	100	79	19	81	1
**7**	CF_3SO_3H 0.1%	toluene	1	85	100	73	22	78	1
**8**	CF_3SO_3H 0.1%	EtOAc	1	0	100	52	37	63	1
**9**	$MeSO_3H$ 20%	2-Me-THF	0.7	80	100	60	84	16	1
**10**	$MeSO_3H$ 7%	heptane	3	90	100	24	86	14	0.5
**11**	CSA 20 %	toluene	0.2	100	0	0	0	0	12

Supplementary Table 6: Influence of the amount of $Fe(ClO_4)_2 \cdot H_2O$ on the rearrangement of 3S-caranepoxide (**2-3S**) to the R- and S-caranketone isomers. All experiments were carried out with a concentration of 1 M at 25 °C for 5 h. Data values refer to TIC-area of GCMS spectra (uncorrected values).

**Entry**	**$Fe(ClO_4)_2 \cdot H_2O$ [mol%]**	**Conversion [%]**	**Σ ketone [%]**	**3-3R [%]**	**3-3S [%]**
**1**	0.1	90	71	20	80
**2**	0.25	95	69	14	86
**3**	0.5	98	67	15	85
**4**	1	100	63	15	85
**5**	5	100	54	28	72

Supplementary Table 7: Influence of the concentration of 3S-caranepoxide (**2-3S**) on the rearrangement to ketones **3-3S** and **3-3R**. All experiments were carried out with a concentration of 1 M at 25 °C and 0.2 % $Fe(ClO_4)_2 \cdot H_2O$ for 7 h. Data values refer to TIC-area of GCMS spectra (uncorrected values).

**Entry**	**$C_{S\text{-caranepoxide}}$ [M]**	**Conversion [%]**	**Σketone [%]**	**3-3R [%]**	**3-3S [%]**
**1**	0.1	<1	-	-	-
**2**	0.25	8	56	20	80
**3**	0.5	78	74	13	87
**4**	1.0	100	70	10	90
**5**	2.0	100	67	10	90
**6**	3.0	100	66	10	90

Supplementary Table 8: Comparison of temperature effects on combinations of Fe(ClO4)$_2$·H_2O (0.2%) in cyclohexane and CF_3SO_3H in hexane/pentane (0.1%).

**Entry**	**Catalyst [%]**	**Solvent**	**C [M]**	**T [°C]**	**Conversion [%]**	**Σ ketone [%]**	**3-3R [%]**	**3-3S [%]**	**t [h]**
**1**	$Fe(ClO_4)_2 \cdot H_2O$	cyclohexane	1	25	100	70	10	90	7
**2**	$Fe(ClO_4)_2 \cdot H_2O$	cyclohexane	1	60	100	82	15	85	0.1
**3**	CF_3SO_3H	pentane	0.5	25	100	68	13	87	1
**4**	CF_3SO_3H	hexane	0.5	50	100	67	15	85	1

Supplementary Table 9: Influence of the solvent on the isomerization of a 3S-caranketone (**3-3S**) enriched solution (purity 79%, R-caranketone 11%, S-caranketone 89%). All experiments were carried out with a concentration of 1 M and HCl (6.0%, from a 2 M solution) as isomerization agent. Samples were taken after stirring for 6 h at room temperature (a), additional 15 h at room temperature (b) and additional 48 h at 60 °C (c). Data values refer to TIC-area of GCMS spectra (uncorrected values).

**Entry**	**Solvent**	**Relative polarity***	**Σ ketone [%]**			**3-3R [%]**			**3-3S [%]**		
			a	b	c	a	b	c	a	b	c
**1**	Cyclohexane	0.006	77	77	70	20	20	74	80	80	26
**3**	Toluene	0.099	77	76	70	21	21	79	79	79	21
**4**	THF	0.207	76	76	73	78	85	78	22	15	22
**5**	EtOAc	0.228	77	77	73	55	85	78	45	15	22
**6**	Chloroform	0.259	77	77	73	21	21	23	79	79	77
**7**	Acetone	0.355	78	78	30	85	85	100	15	15	0
**8**	MeCN	0.460	78	78	20	80	82	100	20	18	0
**9**	EtOH	0.654	77	77	73	25	36	79	75	64	21
**10**	MeOH	0.762	78	78	70	33	57	76	77	43	24

* data retrieved from Christian Reichardt, Solvents and Solvent Effects in Organic Chemistry, Wiley-VCH Publishers, 3rd ed., 2003.

Supplementary Table 10: GPC-parameters applied for analysis of terpene-based polyamides.

Parameters	Values
column temperature	35 °C
flow	0.6 mL/min
elution solvent	0.05 M NaTFA in HFIP
sample concentration	1.0 mg/mL
injection volume	50 µL
elution time	30 min
elution volume	18.0 mL
column 1	PSS PFG pre-column
column 2	PSS PFG 100 Å
column 3	PSS PFG 1000 Å

Supplementary Table 11: Effect of the reaction temperature and the activator concentration on the molecular weights. Polymerization method A was applied. Conditions: 1.8 mmol **5-3S**, 2.0 – 5.5 mol% NaH on paraffin, 180 °C or 220 °C, 1 h, work up method A. M/A ratio = Monomer/Activator ratio. Molecular weights refer to masses over 1.0 kDa.

**Entry**	**M/A ratio**	**M_n [kDa]**	**M_w [kDa]**	**M_P [kDa]**	**P_D**	**T_g [° C]**	**T_m [° C]**
**180 °C A)**	90	10.2	16.2	14.0	1.59	115	250-280
**180 °C B)**	50	9.3	14.5	12.6	1.56	115	260-285
**180 °C C)**	46	9.1	14.1	12.5	1.55	113	255-285
**180 °C D)**	24	6.7	9.5	9.4	1.42	111	245-280
**180 °C E)**	16	5.9	8.3	8.4	1.41	105	245-280
**220 °C F)**	100	7.5	9.6	9.4	1.28	112	240-270
**220 °C G)**	96	7.1	9.0	9.0	1.27	112	230-270
**220 °C H)**	25	6.0	7.5	7.8	1.26	110	240-270
**220 °C I)**	16	5.6	7.3	7.4	1.30	108	230-270

Supplementary Table 12: DSC method B, method without tempering segment.

Segment	Temperature [°C] Start/End	heating rate [K/min]	N_2 [mL/min]
1	-20 (2 min)	isotherm	50
2	-20/320	10	50
3	320	isotherm	50
4	320/-20	10	50
5	-20 (1 min)	Isotherm	50
6	-20/320	10	50
7	320 (1 min)	Isotherm	50
8	320/-20	10	50
9	-20 (1 min)	Isotherm	50
10	-20/320	10	50

Supplementary Table 13: Impact of the activator concentration on the conversion. The conversion was verified by NMR (closed lactam -NH-C*H*CH$_3$-CH$_2$- compared to open lactam -NH-C*H*CH$_3$-CH$_2$-, see also Supplementary Figure 12) and GPC (M_n > 1.0 kDa). Conditions: 3.0 mmol **5-3S** or **5-3R**, 3.0 mol% NaH on paraffin, 190 °C, 1 h, polymerization method B, work-up method B. Conv. = conversion. M/A ratio = Monomer/Activator ratio. M/A CCR = Monomer/Activator conversion-correlated ratio [($n_{Monomer}$* conversion [%] * 100^{-1}) * $n_{Activator}^{-1}$].

Entry	M/A ratio	GPC conv. [%]	NMR conv. [%]	M/A CCR	M_n [kDa]	M_w [kDa]	PD	T_g [° C]
**poly5-3R-E1**	1250	45	39	500	19.9	32.9	1.65	119
**poly5-3R-E2**	789	57	53	418	20.2	43.8	2.17	120
**poly5-3R-E3**	330	84	85	281	19.6	45.6	2.33	117
**poly5-3R-E4**	158	84	88	134	15.2	36.3	2.38	116
**poly5-3R-E5**	107	86	88	93	13.5	31.8	2.35	115
**poly5-3R-E6**	79	86	91	69	13.1	29.7	2.27	117
**poly5-3S-E7**	150	1	1.5	5	3.3	7.3	2.22	-
**poly5-3S-E8**	107	19	19	24	6.6	13.7	2.08	-
**poly5-3S-E9**	79	65	64	54	8.6	16.9	1.97	-
**poly5-3S-E10**	54	71	69	43	8.4	15.2	1.81	-
**poly5-3S-E11**	38	87	88	36	7.6	13.9	1.83	-
**poly5-3S-E12**	27	85	84	26	7.1	12.8	1.79	-

Supplementary Table 14: Impact of the reaction time on the conversion and the state. Reaction conditions: 3S-caranlactam (**5-3S**, 1.80 mmol), NaH (60% on paraffin, 6.0 mg, 0.15 mmol) and **Bz5-3S** (15.0 mg, 0.06 mmol), 180 °C, polymerization method B.

**Entry**	**Reaction time [min]**	**Integral** 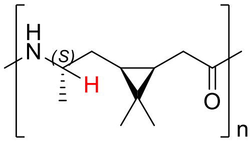 **poly5-3S**	**Integral** 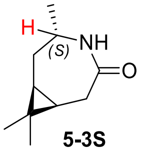 **5-3S**	**Conversion [%]**	**State**
**A**	5	1.00	1.03	49	viscous oil, yellow
**B**	15	1.00	0.63	61	very viscous oil, yellow
**C**	30	1.00	0.48	68	brittle solid, yellow
**D**	45	1.00	0.32	76	brittle solid, yellow
**E**	120	1.00	0.23	81	brittle solid, brown

Supplementary Table 15: DSC method A, method for thermal analysis of polyamides including a tempering segment (4).

**Segment**	**Temperature [°C] Start/End**	**heating rate [K/min]**	**N_2 [mL/min]**
1	20/350	20	50
2	350/20	-20	50
3	20/220	10	50
4	220 (20 min)	isotherm	50
5	220/0	-10	50
6	0/370	10	50
7	370/0	-10	50
8	0/440	10	50

Supplementary Table 16: Built-in was investigated by NMR. * = Application of Ac_2O and NaH for in-situ generated **Ac5-3S** as activator instead of **Bz5-3S**. # = detection limit insufficient.

				**Reaction Conditions**					
**Entry**	**5-3S [%]**	**53-R [%]**	**LL [%]**	**CL [%]**	**M/A ratio**	**T [°C]**	**t [min]**	**NaH [%]**	**Polymerization method**
**E1**	15	85			333	190	20	3.0	B
**E2**	50	50			64*	190	30	4.2	A*
**E3**	83	17			73	190	60	2.5	B
**E4**	30		70		50	170	30	1.6	A
**E5**	41		59		44	190	30	5.6	A
**E6**	37		63		400	190	30	1.6	A
**E7**	50		50		70	190	60	3.8	B
**E8**	85		15		20	190	60	2.0	A
**E9**	25			75	213	190	30	2.1	A
**E10**	50			50	86	190	60	3.0	B
**E11**	75			25	53	175	60	1.9	B
				**Analytical Data**					
**Entry**		**Built-in [%]**			**M_n [kDa]**	**M_w [kDa]**	**T_g [°C]**	**T_m [°C]**	**PD**
	**5-3S**	**53-R**	**LL**	**CL**					
**E1**	0#	100#			19.8	38.9	111	-	1.96
**E2**	30	70			no data	no data	111	-	no data
**E3**	75	25			10.4	15.0	109	210-250	1.43
**E4**	27		73		no data	no data	43	120-150	no data
**E5**	32		68		12.5	24.5	46	-	1.96
**E6**	33		67		30.2	60.1	49		1.99
**E7**	41		59		10.0	15.6	55	-	1.56
**E8**	83		17		no data	no data	82	220-240	no data
**E9**	18			82	15.2	31.1	62	160-190	2.04
**E10**	48			52	12.1	17.3	88	-	1.42
**E11**	73			27	no data	no data	94	-	no data

Supplementary Table 17: Incorporation of 5-3S in co-polymerization with CL or LL at different reaction times.

Poly(5-3S$_{50\%}$·CL$_{50\%}$)			
	Co-polymer composition [%]		
Time [s]	Incorporated **5-3S**	Incorporated **CL**	Total conversion [%]
30	0	100	2.8
60	18	82	6.7
120	30	70	14.7
180	35	65	28.7
240	37	63	41
360	39	61	47.9
420	41	59	59.4
3600	49	51	84

Poly(5-3S$_{50\%}$·LL$_{50\%}$)			
	Co-polymer composition [%]		
Time [s]	Incorporated **5-3S**	Incorporated **LL**	Total conversion [%]
30	100	0	0.39
60	66	34	7.9
120	63	37	21.7
180	59	41	31.1
240	58	42	34.5
3600	52	48	64.4

Supplementary Table 18: Outline of the determined crystal structures.

Name	SG	a, b, c [Å]	α, β, γ [°]	V [Å^3]	ρ [g cm^3]
**5-3S**	C121	10.302(10)	90.00	969.0(17)	1.147
		7.635(8)	101.37(4)		
		12.566(14)	90.00		
**5-3R**	$P12_11$	6.716(4)	90.00	949.5(9)	1.171
		8.133(4)	91.38(2)		
		17.370(10)	90.00		
**Poly5-3S**	$P2_12_12_1$	9.79(6)	90.00	952(11)	1.167
		15.10(6)	90.00		
		6.44(6)	90.00		

3. Supplementary Methods

Analytical methods and instruments

Gas chromatography/Mass spectrometry (GC/MS). GC analysis was performed using GC-2010 Plus (Shimadzu) in combination with auto-injector AOC-5000 (Jain, Combi PAL). Separation was achieved via GC capillary column (BPX 5: 5% phenyl, 95% methyl polysilphenylene / siloxane; SGE). For coupled MS, MS-QP2010 Plus (Shimadzu) with electron ionization (70 eV) was used. Software analysis of measured data was accomplished with GC-MS Postrun Analysis (Shimadzu). Gathered data was compared with the National Institute of Standards and Technology database version 08. Supplementary Table 1 displays the used parameters and retention times.

Nuclear magnetic resonance (NMR) spectroscopy. All NMR-measurements were carried out on a JNM-ECA 400 MHz spectrometer from JEOL at 25 °C using standard pulse programs. Chemical shifts are reported as δ-values in ppm. Coupling constants (J-values) are given in Hertz (Hz). The DEPT135° technique was used to assign CH_2-signales. 2D NMR methods (COSY, HSQC, HMBC) were applied if useful. Chemical shifts are reported as follows: value (multiplicity, coupling constant(s) where applicable, number of protons). Only clearly identifiable peaks are assigned. For the characterization of observed signal multiplicities, the following abbreviations were applied: s (singlet), d (doublet), dd (double doublet), dt (double triplet), t (triplet), q (quartet), quint (quintet) and m (multiplet). The data was evaluated with JEOL Delta v5.0.4.4 or MestReNova 6.0.2. For polymer sample preparation, the polyamide (10-15 mg) was dissolved in 600 µL of DCOOD and shook until a homogenous, clear solution was obtained. The samples were measured immediately. As displayed in the spectra, the signals, as typical for polymers, are broadened. Therefore, no conclusive multiplet analysis was possible.

Thin layer chromatography (TLC). TLC was performed using aluminium plates coated with SiO_2 (Merck 60, F-254) and the spots were visualized with a $KMnO_4$ stain. Flash column was performed using SiO_2 (0.06-0.2 mm, 230-400 mesh ASTM) from Roth.

Size exclusion chromatography (SEC). SEC was performed by a SECcurity GPC system with an autosampler (1260 Infinity, Agilent Technologies) and a TCC6000 column oven (Polymer Standards Service, PSS). As columns, a system of three Novema GPC/SEC columns was used (1: 50x8 mm, 10 µm; 2: 300x8 mm, 10 µm, 100 Å; 3: 300x8 mm, 10 µm, 3000 Å). The data was evaluated using PSS WinGPC UniChrom (PSS). PMMA was chosen for narrow molar mass standard calibration and PA6 standards from a PSS ready-call-kit (M_w/M_n = 31400/17400 Da; 22000/13000 Da; 17200/11300 Da) for the broad calibration. For sample preparation, the polyamide was dissolved in a solution of 0.05 M sodium trifluoroacetate (NaTFA) and hexafluoro-iso-propanol (HFIP). Supplementary Table 10 displays the applied GPC parameters.

Differential Scanning Calorimetry (DSC). DSC was performed on a DSC 1 from Mettler Toledo with the software STARe V. 16.00. The samples (5-10 mg) were prepared in alumina crucibles. Supplementary Tables 15 and 11 displays the applied methods DSC method A and DSC method B. For method A, the segments 6 and 7 were used for verification of T_g and T_m if not otherwise stated. For method B, the last heating run was used for evaluation.

Matrix-assisted laser desorption/ionization – time-of-flight (MALDI-TOF). MALDI-TOF was conducted on a Bruker Ultra Flex TOF/TOF mass spectrometer. α-cyano-4-hydroxycinammic acid or dithranol was applied as the matrix. HFIP or formic acid were used as solvents.

Single crystal X-ray diffraction. Monomers were analysed by single-crystal X-ray diffractometry (SC-XRD, D8 Venture, Bruker AXS, Madison, WI, USA) equipped with a 4-circle goniometer (Kappa geometry), a CMOS detector (Photon 100, Bruker AXS), a rotating anode (TXS, Bruker AXS) with MoKα radiation (λ=0.71073 Å), and a multilayer mirror monochromator (HELIOS, Bruker AXS), using the APEX 2 software package (version 2008.4., Bruker AXS). The measurements were performed on single crystals coated with perfluorinated ether, frozen under a stream of cold nitrogen at 100 K. A matrix scan was used to determine the initial lattice parameters. Reflections were merged and corrected for Lorenz and polarization effects, scan speed, and background using a narrow-frame algorithm (SAINT version 7.56a, Bruker AXS). Absorption corrections were performed using SADABS (version 2008/1, Bruker AXS). Space group assignments were based upon systematic absences, E statistics, successful solution (using SHELXT [1]) and refinement of the structures. These were solved by direct methods with the aid of successive difference Fourier maps, and were refined against all data using SHELXL-2014[2] in conjunction with SHELXLE.[3] If not mentioned otherwise, non-hydrogen atoms were refined with anisotropic displacement parameters. Hydrogen atoms were placed in ideal positions using the SHELXL riding model. Full-matrix least-squares refinements were carried out by minimizing $\Sigma\ w(F^2{}_0\text{-}F^2{}_C)^2$ with the SHELXL-20142 weighting scheme. Neutral atom scattering factors for all atoms and anomalous dispersion corrections for the non-hydrogen atoms were taken from *International Tables for Crystallography*.[4] For 3S-caranlactam (**5-3S**), a clear colourless fragment-like specimen of $C_{10}H_{17}NO$, approximate dimensions 0.060 mm x 0.221 mm x 0.263 mm, was used. A total of 1228 frames were collected, for a total exposure time of 2.96 hours.

For 3R-lactam (**5-3R**), a clear colourless fragment-like specimen of $C_{10}H_{17}NO$, approximate dimensions 0.204 mm x 0.214 mm x 0.445 mm, was used. A total of 1420 frames were collected, for a total exposure time of 0.74 hours.

Powder X-ray diffraction. Polymers were assessed by powder X-ray diffraction in Bragg-Brentano geometry (PXRD, Miniflex, Rigaku, Japan, with silicon strip detector D/teX Ultra). Copper Kα radiation was used and sample holders rotated around the axis at half the scattering angle 2Θ to reduce effects of texture. Intensities were recorded in steps of Θ = 0.02°, using incident and receiving Soller slits with

angular apertures of 2.5° and 1.0°. 3S- and 3R-polycaranamide (**poly5-3S**, **poly5-3R**) powders were compressed on low-background holders (cut silicon wafers) with flat recesses. Also, scattering patterns of solvent-cast thin films of **poly5-3S** were recorded. In the latter, crystallization was effectively quenched through rapid solvent evaporation, allowing to record amorphous reference patterns. For presentation, scattering patterns from S- and R-monomers, deposited from solution, were recorded. All patterns were corrected for their sample holder backgrounds.

For **5-3S**, the amorphous reference was scaled to give the volume fraction of crystalline phase, *fc* via the fractions of integral intensities. Its structure was determined by the direct space method simulated annealing (SA) in Expo2014.[5] In detail, molecular models retaining the hydrogen atoms at the three chiral centres were used. The distance between the bonding atoms C1-C6 was flexibly constrained to 1.54 pm, the distance between N1 and O1 to 2.85 pm. An initial selection of unit cells and space groups (SG) was made, based on reflex positions, indexed by N-TREOR09.[6] For each cell, ten SA runs were performed with a resolution of 1.8 Å. Each result was characterized by a figure of merit CF (lower meaning better match). The results were discriminated, based on the CF and the requirements that the obtained crystalline phases possess:

- Densities $\rho_{c,p}$ matching the density of the corresponding monomeric crystals $\rho_{c,m}$ as $\rho_{c,p} \in \rho_{c,m} \pm \rho_{c,m}/5$.

- Structures that are chemically plausible, in particular with respect to the bond angle between C1 and C6.

The cells yielding positive results were explored by additional 50 SA runs, with subsequently added hydrogens. They were then tested for their physical plausibility by semi-empirical geometry optimization by the Molecular Orbital Package (MOPAC, PM7 algorithm, singlet state).[7] As the final figure of merit (FOM), we multiplied the root mean square displacement (RMSD) between the results and their MOPAC-optimized geometries with their respective CF.

Synthetic procedures

Unless otherwise stated, all chemicals, solvents and starting materials were commercially available and used as received. All intermediates were separated from side products by distillation, column chromatography or crystallization until combined data of GCMS and NMR could verify the product and subsequent reactions could be performed satisfactorily. For the enzymatic epoxidation, Novozyme-435 (CALB lipase immobilized on acrylic resin) was applied.

Synthesis of 3S-caranepoxide (2-3S) ***(1S,3S,5R,7R)-3,8,8-trimethyl-4-oxatricyclo[5.1.0.03,5]octane*** **(Enzyme catalysis)**. (+)-3-carene (**1**) (92.2 g, 677 mmol, 1.00 equiv.) was dissolved in EtOAc (2.80 L) and heated under stirring to 50 °C. Novozyme-435 (20 g) was added followed by portions of H_2O_2 (35%, 80.4 mL, 918 mmol, 1.40 equiv., 13.4 mL every hour). The reaction mixture was stirred over night until full conversion of the substrate and the enzyme was removed by filtration. The organic phase was washed with Na_2HCO_3 (saturated solution, 1.50 L) Na_2SO_3 (saturated solution) until a peroxide test (*Quantofix peroxide 100*) was negative. The combined aqueous layers were extracted with toluene. The organic layers were dried using $MgSO_4$ and the organic solvent was removed under reduced pressure to give crude 3S-caranepoxide (**2-3S**, 98.5 g, 646 mmol, 96%) as yellow oil. The purity of the crude product was above 95% (GCMS, NMR) and could be used without further purification. The yield after distillation was 84%.

Synthesis of 3S-caranepoxide (2-3S) ***(1S,3S,5R,7R)-3,8,8-trimethyl-4-oxatricyclo[5.1.0.03,5]octane*** AcOOH (38%, 170 mL, 800 mmol 1.10 equiv.) were given to a solution of $NaOAc·3H_2O$ (130 g, 950 mmol, 1.30 equiv.) in water 500 mL) and heated in an oil bath until the mixture reached a temperature of 30 °C. (+)-3-carene (**1**) (100 g, 730 mmol, 1.00 equiv.) was dropped to the solution within a period of 0.5 h under vigorous stirring. Without external heating, the temperature of the reaction mixture increased to 60 °C. The full conversion of (+)-3-carene (**1**) was reached after 1 h, as monitored by GCMS. After cooling to room temperature, pentane (200 mL) and NaCl (saturated solution, 200 mL) were added and the layers of the reaction mixture were separated. The organic layer was washed with NaOH solution (1 M, 2x250 mL), Na_2SO_2 (saturated solution, 1x250 mL) and water (1x250 mL) and dried over $MgSO_4$. All layers were tested for remaining peracid species. After the solvent was removed under reduced pressure, 100 g of the crude product were distilled (45 °C, 2 mbar) to yield 3S-caranepoxide (**2-3S**, 92 g,605 mmol, 82%) as colourless oil.

^{1}H NMR (400 MHz, DMSO-d6): δ/ppm = 2.76 (s, 1H, -CH_2-C*H*O-CCH_3O-), 2.15 (ddd, J = 16.3, 9.1, 1.9 Hz, 1H, -CCH_3O-C*H_2*-CH-), 2.03 (dd, J = 16.1, 9.1 Hz, 1H, -CHO-C*H_2*-CH-), 1.55 (dt, J = 16.3, 2.4 Hz, 1H, -CCH_3O-C*H_2*-CH-), 1.42 (dd, J = 16.1, 2.3 Hz, 1H, -CHO-C*H_2*-CH-), 1.16 (s, 3H, -CHC*H_3*-), 0.98 (s, 3H, -CCHCHC*H_3*CH_3-), 0.69 (s, 3H, 3H, -$CCHCHCH_3$C*H_3*-), 0.43 (td, J = 9.1, 2.3 Hz, 1H, -CHO-CH_2-C*H*-), 0.35 (td, J = 9.1, 2.4 Hz, 1H, -CCH_3O-CH_2-C*H*-).

^{13}C NMR (100 MHz, DMSO-d6): δ/ppm = 56.6 ($-\underline{C}CH_3O-$), 54.8 ($-\underline{C}HO-$), 27.5 ($-CCHCH\underline{C}H_3CH_3-$), 22.9 ($-CHO-\underline{C}H_2-CH-$), 22.9 ($-C\underline{C}H_3O-$), 18.8 ($-CCH_3O-\underline{C}H_2-CH-$), 15.7 ($-CHO-CH_2-\underline{C}H-$), 15.5 ($-\underline{C}CHCHCH_3CH_3-$), 14.4 ($-CCHCHCH_3\underline{C}H_3-$), 13.6 ($-CCH_3O-CH_2-\underline{C}H-$).

MS (EI, 70 eV): m/z (%) = 152.10 (0.94), 151.05 (0.48), 138.15 (3.64), 137.10 (34.48), 136.15 (0.79), 135.10 (0.77), 134.10 (2.37), 133.15 (0.37), 124.10 (1.81), 123.10 (14.74).

MS (EI, 70 eV): % (m/z) = 100.00 (43.05), 83.40 (67.10), 67.72 (41.10), 63.07 (109.10), 44.70 (81.10), 44.59 (39.10), 34.48 (137.10), 32.83 (79.10), 30.62 (55.05), 27.86 (95.10).

Synthesis of 3R-caranepoxide (2-3R) ***(1S,3R,5S,7R)-3,8,8-trimethyl-4-oxatricyclo[5.1.0.03,5]octane***. (+)-3-Carene (**1**, 50 g, 370 mmol, 1.00 equiv.) were dissolved in acetone (200 mL) and H_2O (200 mL) and cooled in an ice bath before N-bromsuccinimide (72.0 g, 400 mmol, 1.08 equiv.) were added portion-wise while the temperature of the reaction mixture was kept below 10 °C. After the addition was complete, the reaction was allowed to reach room temperature and was then stirred for 1 h before another portion of N-bromsuccinimide (5.00 g, 28.0 mmol, 0.08 equiv.) was added without cooling. When the conversion of (+)-3-carene (**1**) was complete, NaOH (5 M, 250 mL) were dropped to the reaction mixture and stirred at room temperature for 12 h. After extraction with hexane (2x200 mL), the combined organic layers were washed with Na_2SO_3 (saturated solution, 250 mL) and NaCl (saturated solution, 250 mL) and then dried over $MgSO_4$. The solvent was removed under reduced pressure and the crude product was purified by fractionated vacuum distillation (55-90 °C, 3 mbar) to yield 3R-caranepoxide (**2-3R**, 26.1 g, 170 mmol, 46 %, purity: 97 % 3R-caranepoxide (**2-3R**) and 3.0 % 3S-caranketone (**3-3S**) as verified by GCMS) as colourless oil.

^{1}H NMR (400 MHz, DMSO-d6): δ/ppm = 2.84 (d, J = 5.4 Hz, 1H, $-CCH_3CO-C\underline{H}O-CH_2-$), 2.29 (ddd, J = 16.7, 9.2, 5.4 Hz, 1H, $-CHO-C\underline{H_2}-CH-$), 2.08 (dd, J = 16.4, 9.2 Hz, 1H, $CCH_3O-C\underline{H_2}-CH-$), 1.71 – 1.61 (m, 2H, $-CHO-C\underline{H_2}-CH-$, $-CCH_3O-C\underline{H_2}-CH-$), 1.22 (s, 3H, $-CC\underline{H_3}O-$), 0.94 (s, 3H, $-CCHCHC\underline{H_3}CH_3-$), 0.88 (s, 3H, $-CCHCHC\underline{H_3}CH_3-$), 0.61 – 0.48 (m, 2H, $-CHO-CH_2-C\underline{H}-$, $-CCH_3O-CH_2-C\underline{H}-$).

^{13}C NMR (100 MHz, DMSO-d6): δ/ppm = 56.8 ($-\underline{C}HO-$), 55.07 ($-\underline{C}CH_3O-$), 28.86 ($-CCHCH\underline{C}H_3CH_3-$), 24.47 ($-C\underline{C}H_3O-$), 23.33 ($-CHO-\underline{C}H_2-CH-$), 19.16 ($CCH_3O-\underline{C}H_2-CH-$), 17.70 ($-CHO-CH_2-\underline{C}H-$), 16.84 ($CCH_3O-CH_2-\underline{C}H-$), 16.68 ($-\underline{C}CHCHCH_3CH_3-$), 14.76 ($-CCHCHCH_3\underline{C}H_3-$).

MS (EI, 70 eV): m/z (%) = 152.20 (1.27), 138.10 (1.91), 137.15 (17.91), 136.15 (0.53), 134.10 (1.17), 124.15 (1.31), 123.15 (10.85), 122.15 (1.08), 121.15 (2.56), 120.15 (1.05).

MS (EI, 70 eV): % (m/z) = 100.00 (79.10), 93.02 (43.05), 67.30 (67.10), 52.62 (41.05), 44.01 (109.10), 42.23 (94.10), 34.09 (81.10), 29.28 (93.10), 27.58 (39.05), 26.27 (95.10).

Synthesis of 3S-caranketone (3-3S) ***(1R,4S,6S)-4,7,7-trimethylbicyclo[4.1.0]heptan-3-one***. 3S-caranepoxide (**2-3S**, 43.6 g, 280 mmol, 1.00 equiv.) was dissolved in cyclohexane (230 mL) and heated to 60 °C. $Fe(ClO_4)_2 \cdot H_2O$ (167 mg, 0.2 mol%) in EtOAc (1 mL) was added. The rearrangement was completed after 1.5 h. GCMS verified a *de* of 95%. The mixture was washed with HCl (2.0 M, 2x50 mL), $NaHCO_3$ (saturated solution, 100 mL) and water (100 mL). Cyclohexane was removed under reduced pressure and 15.0 g of the crude product were purified by fractional vacuum distillation (100 – 140 °C, 10 mbar) to yield 3S-caranketone (**3-3S**, 8.0 g, 53%) as colourless oil.

^{1}H NMR (400 MHz, DMSO-d6): δ/ppm = 2.56 – 2.47 (m, 1H, -CO-C*H*$_2$-CH-, superposition by solvent peak), 2.10 (qdd, J = 7.3, 5.0, 2.7 Hz, 1H, -C*H*CH$_3$-), 2.03 – 1.90 (m, 2H, -CHCH$_3$-C*H*$_2$-CH-, -CO-C*H*$_2$-CH-), 1.70 – 1.62 (m, 1H, -CHCH$_3$-C*H*$_2$-CH-), 1.13 (d, J = 7.2 Hz, 3H, -CHC*H*$_3$-), 1.04 (s, 3H, -CCHC*H*$_3$CH$_3$-), 1.03 – 0.97 (m, 1H, -CO-CH$_2$-C*H*-), 0.90 (s, 3H, -CCHCH$_3$C*H*$_3$-), 0.80 (td, J = 8.9, 6.4 Hz, 1H, -CHCH$_3$-CH$_2$-C*H*-).

^{13}C NMR (100 MHz, DMSO-d6): δ/ppm = 216.1 (-*C*O-), 40.7 (-*C*HCH$_3$-), 33.9 (-CO-*C*H$_2$-CH-), 27.8 (-CCHCH*C*H$_3$CH$_3$-, 26.3 (-CHCH$_3$-*C*H$_2$-CH-), 21.1 (-CO-CH$_2$-*C*H-), 19.0 (-*C*CHCHCH$_3$CH$_3$-), 16.7 (-CH*C*H$_3$-), 16.4 (-CO-CH$_2$-*C*H-), 14.6 (-CCHCHCH$_3$*C*H$_3$-).

MS (EI, 70 eV): m/z (%) = 153.10 (2.77), 152.10 (27.38), 138.10 (1.16), 137.10 (12.12), 135.15 (0.47), 134.10 (2.59), 125.15 (0.48), 124.10 (4.38), 123.10 (4.10), 121.10 (0.54).

MS (EI, 70 eV): % (m/z) = 100.00 (67.10), 83.36 (81.10), 69.63 (41.10), 45.24 (39.10), 44.25 (82.10), 33.71 (95.10), 32.68 (109.10), 30.74 (110.10), 27.40 (55.10), 27.38 (152.10).

Synthesis of 3R-caranketone (3-3R) ***(1R,4R,6S)-4,7,7-trimethylbicyclo[4.1.0]heptan-3-one*** **by rearrangement of 3R-caranepoxide (2-3R).** 3R-caranepoxide (**2-3R**, 10.0 g, 66 mmol, 1.00 equiv.) was dissolved in cyclohexane (55 mL) and heated to 60 °C before a solution of $Fe(ClO_4)_2 \cdot H_2O$ (78 mg, 0.30 mmol, 0.005 equiv.) in EtOAc (0.5 mL) was dropped to the reaction mixture and stirred for 3 h. GCMS revealed a *de* of 100%. After complete conversion, Na_2SO_3 (saturated solution, 150 mL) was added and the layers were separated. The organic layer was washed with water (1x100 mL) and the solvent was removed under reduced pressure. Crude product (8.80 g) was purified by fractional vacuum distillation (105 °C, 30 mbar) to yield a mixture of caranketones (5.74 g, 57 % purity > 80% as confirmed by GCMS and NMR, R 82%, S 18%). As no 3S-caranketone (**3-3S**) was present before the distillation and a heat-induced isomerization was not observed for pure 3R-caranketone (**3-3R**), it is likely that the 3S-caranketone (**3-3S**) formation is promoted by residual bromide species that could not be removed by the washing steps.

Synthesis of 3R-caranketone (3-3R) ***(1R,4R,6S)-4,7,7-trimethylbicyclo[4.1.0]heptan-3-one*** **by isomerization of 3S-caranketone (3-3S) and subsequent enrichment of 3R-caranketone (3-3R) by reaction with $HONH_2 \cdot HCl$**. Freshly distilled 3S-caranepoxide (**2-3S**, 88.0 g, 580 mmol, 1.00 equiv.) was

dissolved in cyclohexane (600 mL) and heated to 60 °C. $FeClO_4 \cdot 6H_2O$ (150 mg, 0.58 mmol, 0.001 equiv.) in EtOAc (1.0 mL) was added and stirred overnight until all 3S-caranepoxide (**2-3S**) was converted. After washing with Na_2SO_3 (2.0 w%, 2 x 400 mL), HCl (0.1 M, 500 mL) and NaCl (saturated solution, 500 mL) and drying under use of $MgSO_4$, the organic solvent was removed under reduced pressure to give crude 3S-caranketone (**3-3S**, 83.8 g) that was then dissolved in MeCN (500 mL). For the isomerization to 3R-caranketone (**3-3R**), HCl (2 M, 120 mL) was then dropped to the solution within 30 min under vigorous stirring and then stirred for 48 h until the equilibrium isomeric ratio of 4:1 in favour of the R-isomer was reached. As the S-isomer reacts faster to the corresponding oxime, a further enrichment of the R-isomer was possible. NaOAc·3 H_2O (6.56 g, 80.0 mmol) in water (200 mL) was given to the reaction mixture and stirred for 5 min before $HONH_2 \cdot HCl$ (5.07 g, 73 mmol) was added portion-wise within an hour. After stirring for 4 h, the reaction mixture was investigated by GCMS. The whole process is displayed in Supplementary Figure 17. The layers were separated, and the aqueous phase was extracted with cyclohexane (3 x 100 mL). The combined organic phases were washed with $NaHCO_3$ (saturated solution, 300 mL) and NaCl (saturated solution, 300 mL) and dried using Na_2SO_4. The crude product was purified by vacuum distillation (60 → 100 °C, 3 mbar) to yield 3R-caranketone (**3-3R**, 41.3 g, 47%, purity > 90 % as confirmed by GCMS and NMR, isomeric ratio > 25:1).

^{1}H NMR (400 MHz, DMSO-d6): δ/ppm = 2.57 (dd, J = 17.6, 8.4 Hz, 1H, -CO-C*<u>H</u>*H_2-CH-), 2.41 – 2.23 (m, 2H, -C<u>H</u>CH_3-CO-, -$CHCH_3$-C$\underline{H_2}$-CH-), 2.12 (dd, J = 17.6, 2.1 Hz, 1H, -CO-C$\underline{H_2}$-CH-), 1.20 – 1.10 (m, 1H, -$CHCH_3$-C$\underline{H_2}$-CH-), 1.06 (td, J = 8.8, 2.3 Hz, 1H, -CO-CH_2-C<u>H</u>-), 1.00 (s, 3H, -CCHCHC$\underline{H_3}$$CH_3$-), 1.00 – 0.92 (m, 1H, -$CHCH_3$-CH_2-C<u>H</u>-), 0.83 (d, J = 6.4 Hz, 3H, -CH_2-CHC$\underline{H_3}$-CO-), 0.78 (s, 3H, -CCHCHC$\underline{H_3}$$CH_3$-).

^{13}C NMR (100 MHz, DMSO-d6): δ/ppm = 215.2 (-<u>C</u>O-), 41.0 (-<u>C</u>HCH_3-), 36.4 (-CO-<u>C</u>H_2-CH-), 29.2 (-$CHCH_3$-<u>C</u>H_2-CH-), 27.6 (-CCHCH<u>C</u>$H_3$$CH_3$-), 22.5 (-CO-$CH_2$-<u>C</u>H-), 19.6 (-$CHCH_3$-$CH_2$-<u>C</u>H-), 18.9 (-<u>C</u>CHCH$CH_3$$CH_3$-), 14.7 (-CCHCH$CH_3$<u>C</u>$H_3$-), 14.1 (-CH<u>C</u>$H_3$-).

MS (EI, 70 eV): m/z (%) = 153.15 (3.05), 152.20 (28.19), 138.20 (1.46), 137.20 (14.60), 135.15 (0.64), 134.15 (2.69), 124.15 (4.17), 123.15 (4.72), 119.15 (3.62), 111.15 (3.35).

MS (EI, 70 eV): % (m/z) = 100.00 (67.10), 87.96 (81.15), 36.29 (41.10), 46.76 (82.15), 37.97 (39.10), 36.57 (95.15), 35.65 (109.15), 34.44 (110.15), 29.04 (55.10), 28.19 (152.20).

Synthesis of 3S- and 3R-caranoxime (4-3S and 4-3R) ***(1R,4S,6S)-4,7,7-trimethylbicyclo[4.1.0]heptan-3-one oxime*** **and** ***(1R,4R,6S)-4,7,7-trimethylbicyclo[4.1.0]heptan-3-one oxime***. 3-caranketone (**3-3S** or **3-3R**, 1.00 equiv.) was dissolved in MeCN (1.25 M) and NaOAc (1.40 equiv.) followed by $HONH_2 \cdot HCl$ (1.20 equiv.) were added sequentially. The suspension was stirred overnight until full conversion was verified by GCMS. The volume was doubled by the addition of water and the layers were separated. The aqueous phase was extracted with EtOAc until no oxime was observed in the aqueous phase and

the combined organic phases were washed with $NaHCO_3$ (saturated solution, equivoluminar) and NaCl (saturated solution, equivoluminar) and dried using Na_2SO_4. The solvent was removed under reduced pressure to give the respective oximes **4-3S** or **4-3R** as a mixture of cis- (15%) and trans-oximes (85%) as very viscous oil in a total yield of 90 %.

3S-caran-*trans*-oxime:

^{1}H NMR (400 MHz, DMSO-d6): δ/ppm = 10.07 (s, 1H, -NO$\underline{H}$), 2.56 (dd, J = 18.6, 1.6 Hz, 1H, -CNOH-C$\underline{H_2}$-CH-), 2.32 – 2.17 (m, 2H,-C$\underline{H}$CH$_3$-, -CNOH-C$\underline{H_2}$-CH-), 1.90 – 1.78 (m, J = 16.8, 8.1, 3.1 Hz, 1H, -CHCH$_3$-C$\underline{H_2}$-CH-), 1.37 (dt, J = 14.4, 4.9 Hz, 1H, -CHCH$_3$-C$\underline{H_2}$-CH-), 1.05 (d, J = 7.1 Hz, 3H, CH$_2$-CHC$\underline{H_3}$-CNOH-), 0.96 (s, 3H, -CCHCHC$\underline{H_3}$CH$_3$-), 0.79 (td, *J* = 8.9, 1.8 Hz, 1H, -CNOH-CH$_2$-C$\underline{H}$-) 0.71 (s, 3H, -CCHCHCH$_3$C$\underline{H_3}$-), 0.69 – 0.62 (m, 1H, CHCH$_3$-CH$_2$-C$\underline{H}$-).

^{13}C NMR (100 MHz, DMSO-d6): δ/ppm = 161.5 (-$\underline{C}$NOH-), 32.8 (-$\underline{C}$HCH$_3$-), 28.5 (-CCHCH$\underline{C}$H$_3$CH$_3$-), 26.8 (-CHCH$_3$-$\underline{C}$H$_2$-CH-), 19.3 (CH$_2$-CH$\underline{C}$H$_3$-CNOH-), 19.1 (-CNOH-CH$_2$-$\underline{C}$H-), 18.3 ($\underline{C}$CHCHCH$_3$CH$_3$), 17.1 (-CNOH-$\underline{C}$H$_2$-CH-), 16.7 (-CHCH$_3$-CH$_2$-$\underline{C}$H-), 14.9 (-CCHCHCH$_3\underline{C}$H$_3$-).

MS (EI, 70 eV): m/z (%) = 168.05 (1.30), 167.00 (11.80), 166.05 (1.66), 153.10 (1.47), 152.05 (15.72), 151.05 (1.69), 150.05 (10.83), 149.05 (1.90), 148.10 (5.97), 139.10 (2.56).

MS (EI, 70 eV): % (m/z) = 100.00 (41.05), 51.40 (39.10), 47.59 (67.05), 43.19 (112.10), 42.42 (79.05), 41.89 (107.10), 40.65 (55.10), 39.11 (106.05), 38.33 (43.05), 29.88 (81.05).

3R-caran-*trans*-oxime:

^{1}H NMR (400 MHz, DMSO-d6): δ/ppm = 10.29 (s, 1H, -CNO$\underline{H}$-), 2.75 (dd, J = 18.1, 1.6 Hz, 1H, -CNOH-C$\underline{H_2}$-CH-), 2.21 – 2.02 (m, 3H, -CNOH-C$\underline{H_2}$-CH-, -C$\underline{H}$CH$_3$-, -CHCH$_3$-CH_2-CH-),0.97 – 0.86 [7H, 2xC$\underline{H_3}$ 1xC$\underline{H}$H: 0.94 (s, 3H, CCHCHC$\underline{H_3}$CH$_3$-), 0.91 (d, J = 6.2 Hz, 3H, -CHC$\underline{H_3}$-), superposition -CHCH$_3$-C$\underline{H_2}$-CH-)] 0.85 – 0.77 (m, 1H, -CNOH-CH$_2$-C$\underline{H}$-), 0.73 (dd, J = 9.0, 2.0 Hz, 1H, CHCH$_3$-CH$_2$-C$\underline{H}$-), 0.70 (s, 1H, -CCHCHCH$_3$C$\underline{H_3}$-).

^{13}C NMR (100 MHz, DMSO-d6): δ/ppm = 160.2 (-$\underline{C}$NOH-), 33.7 (-$\underline{C}$HCH$_3$-), 29.4 (-CHCH$_3$-$\underline{C}$H$_2$-CH-), 28.0 (-CCHCH$\underline{C}$H$_3$CH$_3$-), 19.8 (-CNOH-CH$_2$-$\underline{C}$H-), 19.7 (CNOH-$\underline{C}$H$_2$-CH-), 19.3 (-CNOH-CH$_2$-$\underline{C}$H-), 17.8 ($\underline{C}$CHCHCH$_3$CH$_3$), 16.6 (CH$_2$-CH$\underline{C}$H$_3$-CNOH-), 14.5 (-CCHCHCH$_3\underline{C}$H$_3$-).

MS (EI, 70 eV): m/z (%) = 168.15 (1.95), 167.15 (15.02), 166.15 (2.30), 153.15 (2.14), 152.15 (19.40), 151.20 (2.22), 150.20 (16.60), 149.20 (2.13), 148.15 (7.09), 139.15 (3.59).

MS (EI, 70 eV): % (m/z) = 100.00 (41.10), 59.79 (112.10), 57.23 (67.10), 54.27 (79.10), 51.43 (107.10), 48.84 (106.10), 48.75 (55.10), 48.26 (39.05), 42.55 (43.10), 37.77 (134.15).

Synthesis of 3S- and 3R-caranlactams (5-3S and 5-3R) ***(1R,5R,7S)-5,8,8-trimethyl-4-azabicyclo[5.1.0]octan-3-one*** **and** ***(1S,5S,7S)-5,8,8-trimethyl-4-azabicyclo[5.1.0]octan-3-one***. 3-caranoxime (**4-3S** or **4-3R**, 1.00 equiv.) was dissolved in MeCN (1 M) and cooled in an ice bath before NaOH (2 M, 3.10 equiv.) was dropped to the solution. The reaction mixture was stirred for 2 h and tosyl chloride (1.10 equiv.) was added slowly within 1.5 h. The ice bath was removed after additional stirring for 2 h and kept at room temperature overnight. The aqueous layer was extracted with EtOAc until no product was detected in the aqueous phase and the combined organic layers were then washed with $NaHCO_3$ (saturated solution, equivoluminar) and NaCl (saturated solution, equivoluminar) and dried using Na_2SO_4. The solvent was removed under reduced pressure and the remaining solid was recrystallized from EtOAc at -20 °C to yield pure 3-caranlactam as colourless crystals (3R-caranlactam **5-3R**: 74 %; 3S-caranlactam **5-3S**: 76 %).

3S-caranlactam (**5-3S**)

^{1}H NMR (400 MHz, DMSO-d6): δ/ppm = 6.92 (s, 1H, -CO-N$\underline{H}$-) 3.5 – 3.14 (m, 1H, -NH-C$\underline{H}CH_3$-CH_2-), 2.31 – 2.15 (m, 2H, -CO-C$\underline{H_2}$-CH-), 1.71 – 1.49 (m, 2H, -CH-C$\underline{H_2}$-$CCHCH_3$-), 1.05 (d, J = 6.4 Hz, 3H, -NH-CHC$\underline{H_3}$-), 1.01 (s, 3H, -CC$\underline{H}CHCH_3C\underline{H_3}$-), 0.97 (s, 3H, -CCHCHC$\underline{H_3}CH_3$-), 0.85 – 0.76 (m, 1H, -CC$\underline{H}CHCH_3CH_3$-), 0.57 (td, J = 9.0, 2.1 Hz, 1H, -CCHC$\underline{H}CH_3CH_3$-).

^{13}C NMR (100 MHz, DMSO-d6): δ/ppm = 173.8 (-$\underline{C}$O-), 46.3 (-NH-$\underline{C}HCH_3$-), 30.6 (-CO-C$\underline{H_2}$-CH-), 30.4 (CH-C$\underline{H_2}$-CHCH3-), 28.6 (-CCHCH$\underline{C}H_3CH_3$-), 21.11 (-NH-CH$\underline{C}H_3$-), 20.1 (CO-CH_2-$\underline{C}$H-), 20.1 (-$CHCH_3$-CH_2-$\underline{C}$H-), 17.4 (-$\underline{C}CHCHCH_3CH_3$-), 14.9 (-$CCHCHCH_3\underline{C}H_3$-).

MS (EI, 70 eV): m/z (%) = 168.10 (1.05), 167.15 (8.33), 166.25 (0.70), 154.20 (0.30), 153.20 (4.44), 152.20 (44.99), 151.25 (0.22), 150.20 (0.23), 139.20 (1.42), 138.15 (1.09).

MS (EI, 70 eV): % (m/z) = 100.00 (44.10), 60.39 (67.10), 44.99 (152.20), 44.18 (81.10), 42.43 (82.10), 37.54 (110.15), 35.25 (41.05), 28.11 (57.10), 19.97 (39.05), 19.46 (55.10).

m.P.: 171 °C

3R-caranlactam (**5-3R**)

^{1}H NMR (400 MHz, DMSO-d6): δ/ppm = 6.90 (s, 1H, -CO-N$\underline{H}$-), 3.50 – 3.41 (m, 1H, -NH-C$\underline{H}CH_3$-CH_2-), 2.37 – 2.29 (m, 2H, -CO-C$\underline{H_2}$-CH-), 1.88 – 1.77 (m, 1H, -CH-C$\underline{H_2}$-$CCHCH_3$-), 1.51 – 1.40 (m, 1H, -CH-C$\underline{H_2}$-$CCHCH_3$-), 1.06 (d, J = 6.4 Hz, 3H, -NH-CHC$\underline{H_3}$-), 1.03 (s, 3H, -CC$\underline{H}CHCH_3C\underline{H_3}$-), 0.99 (s, 3H, -CCHCHC$\underline{H_3}CH_3$-), 0.79 – 0.69 (m, 2H, -CC$\underline{H}CHCH_3CH_3$-, -CCHC$\underline{H}CH_3CH_3$-).

^{13}C NMR (100 MHz, DMSO-d6): δ/ppm = 173.1 (-$\underline{C}$O-), 49.6 (-NH-$\underline{C}HCH_3$-), 33.0 (-CO-C$\underline{H_2}$-CH-), 29.7 (CH-C$\underline{H_2}$-CHCH3-), 28.2 (-CCHCH$\underline{C}H_3CH_3$-), 24.0 (-NH-CH$\underline{C}H_3$-), 23.4 (CO-CH_2-$\underline{C}$H-), 21.5 (-$CHCH_3$-CH_2-$\underline{C}$H-, 18.7 -$\underline{C}CHCHCH_3CH_3$-), 14.8 (-$CCHCHCH_3\underline{C}H_3$-).

MS (EI, 70 eV): m/z (%) = 168.10 (1.42), 167.10 (13.20), 166.15 (1.41), 153.15 (3.19), 152.10 (32.36), 139.15 (1.90), 138.15 (1.66), 134.15 (0.71), 127.15 (0.55), 126.15 (5.91).

MS (EI, 70 eV): % (m/z) = 100.00 (44.05), 71.08 (67.05), 59.84 (110.10), 50.77 (81.10), 41.82 (41.05), 41.15 (82.10), 32.36 (152.10), 24.22 (57.10), 22.96 (55.10), 21.80 (99.10).

m.P.: 140 °C

Synthesis of N-benzoyl-3S-caranlactam (Bz5-3S) ***(1R,5S,7S)-4-benzoyl-5,8,8-trimethyl-4-azabicyclo[5.1.0]octan-3-one***. 2-Methyl-THF (30 mL) in a nitrogen atmosphere was cooled in an ice bath and NaH (540 mg 60 % on paraffin wax, 15.5 mmol, 2.60 equiv.) was added. After 10 min 3S-caranlactam (**5-3S**, 1.00 g, 6.0 mmol, 1.00 equiv.) was added portion-wise within 5 min; a rise of temperature was not observed. After stirring for 2 h, BzCl (1.00 mL, 7.80 mmol, 1.30 mmol) was slowly given to the mixture *via* syringe. The mixture was allowed to reach room temperature and was stirred over for 12 h before 20 g ice was added. After extraction with hexane (3x100 mL), the combined organic phases were washed with NaOH (2.0 M, 150 mL), $NaHCO_3$ (saturated solution, equivoluminar) and NaCl (saturated solution, equivoluminar) and dried using Na_2SO_4. The solvent was removed under reduced pressure and the crude product was crystallized from pentane/EtOAc (4:1) at -20 °C to give N-benzoyl-3S-caranlactam (**Bz5-3S**, 930 mg, 3.40 mmol, 57%) as colourless crystals.

^{1}H NMR (400 MHz, DMSO-d6): δ/ppm = 7.66 – 7.61 (m, 2H, *ortho*-$C\underline{H}$-), 7.54 – 7.48 (m, 1H, *para*-$C\underline{H}$-), 7.46 – 7.39 (m, 2H, *meta*-$C\underline{H}$-), 4.22 (pd, *J* = 6.6, 2.6 Hz, 1H, -$C\underline{H}CH_3$-), 2.76 (dd, *J* = 13.9, 10.1 Hz, 1H, -CO-$C\underline{H_2}$-CH-), 2.56 (dd, *J* = 13.9, 6.8 Hz, 1H, -CO-$C\underline{H_2}$-CH-), 2.19 – 2.06 (m, 1H, -$CHCH_3$-$C\underline{H_2}$-CH-), 1.94 – 1.83 (m, 1H, -$CHCH_3$-$C\underline{H_2}$-CH-), 1.26 (d, *J* = 6.6 Hz, 3H, -$CH\underline{H_3}$-), 1.10 (s, 3H, -$CCHCHC\underline{H_3}CH_3$-), 1.07 (s, 3H, -$CCHCHC\underline{H_3}CH_3$-), 0.94 – 0.83 (m, 2H, -$CHCH_3$-CH_2-$C\underline{H}$-, -CO-CH_2-$C\underline{H}$-).

^{13}C NMR (100 MHz, DMSO-d6): δ/ppm = 177.3 (-CH_2-$\underline{C}$O-NBz-), 174.6 (-N-$\underline{C}$O-Ph), 136.5 (-N-CO-*ipso*-$\underline{C}_{Ph}$), 131.7 (2x *ortho*-$\underline{C}_{Ph}$), 128.3 (2x *meta*-$\underline{C}_{Ph}$), 128.2 (*para*-$\underline{C}_{Ph}$), 51.9 (-$\underline{C}HCH_3$-), 34.4 (-CO-$\underline{C}H_2$-CH-), 28.3 (-$CHCH_3$-$\underline{C}H_2$-CH-), 28.2 (-$CCHCH\underline{C}H_3CH_3$-), 20.9 (-CO-$CH_2$-$\underline{C}$H-), 20.0 (-$CHCH_3$-$CH_2$-$\underline{C}$H-), 19.9 (-$\underline{C}CHCHCH_3CH_3$-), 18.8 (-$CH\underline{C}H_3$-), 14.7 (-$CCHCHCH_3\underline{C}H_3$-).

MS (EI, 70 eV): m/z (%) = 273.20 (0.09), 272.10 (0.86), 271.10 (4.65), 270.10 (2.47), 257.05 (0.15), 256.10 (0.84), 253.10 (0.23), 244.15 (0.12), 243.10 (0.90), 242.10 (0.46).

MS (EI, 70 eV): % (m/z) = 100.00 (105.05), 35.39 (77.05), 14.02 (82.05), 12.28 (148.10), 10.27 (67.10), 9.19 (41.05), 9.01 (81.10), 8.20 (166.10), 7.90 (106.05), 6.34 (70.05).

Synthesis of N-benzoyl-3R-caranlactam (Bz5-3R) ***(1R,5R,7S)-4-benzoyl-5,8,8-trimethyl-4-azabicyclo[5.1.0]octan-3-one***. 2-Methyl-THF (50 mL) was cooled in an ice bath under nitrogen atmosphere and NaH (600 mg 60 % on paraffin wax, 17.2 mmol, 1.90 equiv.) and 3R-caranlactam (**5-3R**, 1.50 g, 9.00 mmol, 1.00 equiv.) was added after 5 min. The reaction mixture was stirred for 2 h before BzCl (1.5 mL, 11.7 mmol, 1.30 equiv.) was dropped to the mixture *via* syringe. The reaction mixture was allowed to reach room temperature and after stirring for 12 h, NaH (350 mg 60% on paraffin wax, 10.0 mmol, 1.10 equiv.) and BzCl (0.5 mL, 3.90 mmol, 0.40 equiv.) were added to complete the conversion within 4 h. The reaction was quenched by addition of water. The mixture was extracted with hexane (3x100 mL) and the combined organic layers were washed with NaOH (2.0 M, 150 mL), $NaHCO_3$ (saturated solution, equivoluminar) and NaCl (saturated solution, equivoluminar) and dried using Na_2SO_4. After several crystallizations (EtOAc : hexane/ 1 : 4), N-benzoyl-3R-caranlactam (**Bz5-3R**, 530 mg, 1.90 mmol, 17 %) was obtained as colourless crystals.

^{1}H NMR (400 MHz, DMSO-d6): δ/ppm = 7.51 – 7.39 (m, 5H, -*phenyl-*), 4.36 (dp, J = 12.7, 6.3 Hz, 1H, -C*H*CH$_3$-), 2.65 – 2.54 (m, 2H, -CHCH$_3$-C*H$_2$*-CH-), 2.17 (ddd, J = 15.0, 6.4, 5.1 Hz, 1H,-CO-C*H$_2$*-CH-), 1.44 (dt, J = 15.0, 12.1 Hz, 1H,-CO-C*H$_2$*-CH-), 1.26 (d, J = 6.3 Hz, 3H, -CHC*H$_3$*-), 1.18 (ddd, J = 10.7, 8.9, 6.5 Hz, 1H,-CO-CH$_2$-C*H*-), 1.10 (s, 3H, -CCHCHC*H$_3$*CH$_3$-), 1.10 (s, 3H, -CCHCHCH$_3$C*H$_3$*-), 0.95 (ddd, J = 12.1, 8.8, 5.1 Hz, 1H, -CHCH$_3$-CH$_2$-C*H*-).

^{13}C NMR (100 MHz, DMSO-d6): δ/ppm = 180.51 (-CH$_2$-*C*O-NBz-), 173.93 (-N-*C*O-Ph), 136.79 (-N-CO-*ipso*-*C*$_{Ph}$), 130.68 (2x *ortho*-*C*$_{Ph}$), 128.33 (2x *meta*-*C*$_{Ph}$), 127.44 (*para*-*C*$_{Ph}$), 52.98 (-*C*HCH$_3$-), 34.64 (-CO-*C*H$_2$-CH-), 28.68 (-CHCH$_3$-*C*H$_2$-CH-), 27.82 (-CCHCH*C*H$_3$CH$_3$-), 22.19 (-CH*C*H$_3$-), 21.77 (-CO-CH$_2$-*C*H-), 21.34 (-CHCH$_3$-CH$_2$-*C*H-), 18.86 (-*C*CHCHCH$_3$CH$_3$-), 15.10 (-CCHCHCH$_3$*C*H$_3$-).

MS (EI, 70 eV): m/z (%) = 272.15 (0.53), 271.15 (2.92), 270.15 (3.77), 257.10 (0.56), 256.10 (2.81), 253.10 (0.11), 245.15 (0.10), 244.10 (0.97), 243.15 (4.99), 242.15 (1.28).

MS (EI, 70 eV): % (m/z) = 100.00 (105.10), 34.01 (77.10), 10.07 (82.10), 8.97 (70.10), 8.25 (106.10), 7.47 (67.10), 7.15 (41.10), 6.72 (81.10), 6.55 (148.15), 5.82 (51.05).

Scale-up one-vessel four-step synthesis of 3S-caranlactam (5-3S).

a. **Enzyme catalyzed epoxidation**. (+)-3-carene (**1**, 240 mL, 1.50 mol, 1.00 equiv.) was transferred into the reactor and EtOAc (2.80 L) was added. A pre-washed standard nylon sock (enzyme bag) filled with Novozyme-435 (50 g) was immersed into the mixture and the temperature was set to 60 °C at a stirring rate of 100 rpm min^{-1}. H_2O_2 (30%, 1.65 mol, 1.10 equiv., 12 x 15 mL) was added within 5 h. After the addition was complete, the spent Novozyme-435 was exchanged by new Novozyme-435 (20 g). After another 3 h, the conversion surpassed 98% and the reaction was terminated by removing the enzyme bag. The organic layer was washed with NaOH solution (1.00 M, 2x250 mL), sodium sulphite (saturated solution, 250 mL) and H_2O (1x250 mL). Additional cyclohexane (1.00 L) was added for azeotropic distillation to remove all water residuals (T_{jacket} = 95 °C) until the remaining volume in the reactor was 1.25 L, consisting of crude 3S-caranepoxide (**2-3S**) and cyclohexane.
b. **Epoxidation with diluted AcOOH**. A solution of NaOAc (267 g, 3.25 mol, 1.30 equiv.) and AcOOH (38%, 570 mL 3.25 mol, 1.30 equiv.) in H_2O (1.70 L) was transferred into the reactor and (+)-3-carene (**1**, 341 g ,2.5 mol, 1.00 equiv.) was added portion-wise within 15 min and stirred at room temperature for 2 h. The temperature was set to 30 °C for another 2 h to complete the conversion to 3S-caranepoxide (**2-3S**). During the whole process the stirring speed was set to 300 rpm to guaranty sufficient mixing. After cooling to room temperature, cyclohexane (1.00 L) was added and the aqueous layer was separated *via* the bottom outlet. The organic layer remaining in the reactor was then washed with NaOH solution (1.00 M, 2x500 mL), sodium sulphite (saturated solution, 500 mL) and H_2O (1x500 mL). Additional cyclohexane (2.00 L) was added for azeotropic distillation to remove all water residuals (T_{jacket} = 95 °C) until the remaining volume in the reactor was 2.50 L, consisting of crude 3S-caranepoxide (**2-3S**) and cyclohexane.
c. **Meinwald rearrangement**. For the rearrangement of 3S-caranepoxide (**2-3S**) to 3S-caranketone (**3-3S**), the temperature was set to 60 °C and $Fe(ClO_4)_2{\cdot}H_2O$ (1.28 g, 3.6 mmol, 0.002 equiv.) dissolved in EtOAc (5 mL) was dropped into the mixture within 3 min. The mixture was allowed to reach room temperature after two hours (80% conversion) and was stirred for 12 h to complete the reaction. The catalyst was removed by washing with HCl solution (1 M, 2x500 mL), $NaHCO_3$ (saturated solution, 1.00 L) and water (1.00 L) and cyclohexane was distilled off (T_{jacket} 90 °C – 125 °C).
d. **Oximation**. Acetonitrile (2.00 L) and water (1.00 L) were poured into the vessel and $NaOAc{\cdot}3H_2O$ (238 g, 1.75 mol, 0.70 equiv.) was added. Then, $HONH_2{\cdot}HCl$ (122 g, 1.75 mol, 0.70 equiv.) was added portion-wise within 10 min. After stirring for 2 h at 30 °C, the

conversion was complete. The aqueous layer was separated and analysed by GCMS, revealing no significant amounts of 3S-caranoxime (**4-3S**).

e. **Beckmann rearrangement**. NaOH (260 g, 6.5 mol, 2.60 equiv.) dissolved in water (1.00 L) was slowly dropped to the reaction mixture within 2 h and the temperature was kept beneath 20 °C (T_{jacket} = 15 °C) during the process. After stirring for 1 h, TsCl (333 g, 1.75 mol, 0.70 equiv.) was added in aliquots (28 x 12 g) within 3 h while the temperature was kept beneath 25 °C. GCMS analysis showed that all 3S-caranoxime (**4-3S**) was consumed and NaCl (saturated solution, 200 mL) was added to support phase separation. The aqueous layer was removed and extracted with EtOAc (3 x 600 mL). The combined EtOAc extracts were poured back into the reaction vessel. After washing with $NaHCO_3$ (saturated solution, 500 mL) and NaCl (half-saturated solution, 500 mL) the solvent was removed until the remaining volume was approximately 1.5 L. The solution was cooled down to 15 °C and stirred for 12 h. The crystals were filtered off, washed with water and recrystallized from EtOAc. The mother liquor was cooled to -20 °C in the freezer for 12 h and the formed crystals were filtered off and washed with water. The crystal fractions were combined and recrystallized from EtOAc to give pure 3S-caranlactam (**5-3S**, 101 g, 30 w%, 24 mol%) as colourless crystals (Supplementary Figure 4).

NMR analysis of the polyamides

Poly-3S-caranamide (**poly5-3S**)

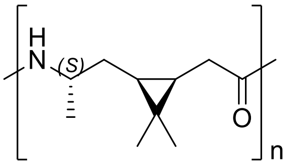

poly5-3S

^{1}H NMR (400 MHz, DCOOD): δ/ppm = 4.19 – 4.04 (1H, -NH-C$\underline{H}$CH$_3$-CH$_2$-, repeating unit), 2.57 – 2.35 (2H, -HN-CO-C$\underline{H_2}$-CH-, repeating unit), 1.66 – 1.53 (2H, -NH-CHCH$_3$-C$\underline{H_2}$-CH-, repeating unit), 1.36 – 1.20 (3H, -NH-CHC$\underline{H_3}$-CH$_2$-, repeating unit), 1.18 – 1.08 (3H, -CO-CH$_2$-CH-CCHCH$_3$C$\underline{H_3}$-, methyl group facing carboxylic group, repeating unit), 1.05 – 1.00 (3H, -NH-CHCH$_3$-CH-CCHC$\underline{H_3}$CH$_3$-, methyl group turned away from carboxylic group, repeating unit), 0.98 – 0.90 (1H, -CO-CH$_2$-C$\underline{H}$-, repeating unit), 0.79 – 0.67 (1H, -NH-CHCH$_3$-CH$_2$-C$\underline{H}$-, repeating unit).

^{13}C NMR (100 MHz, DCOOD): δ/ppm = 176.5 (-$\underline{C}$O-), 47.3 (-NH-$\underline{C}$HCH$_3$-), 31.5 (-CO-$\underline{C}$H$_2$-), 30.9 (-CHCH$_3$-$\underline{C}$H$_2$-), 28.2 (-CCHCH$_3$$\underline{C}H_3$-, methyl group facing carboxylic group), 23.4 (-CO-CH$_2$-$\underline{C}$H-), 21.8 (-CHCH$_3$-CH$_2$-$\underline{C}$H-, 19.6 (-CH$\underline{C}$H$_3$-), 17.9 (-$\underline{C}$CHCHCH$_3$CH$_3$-), 14.7 (-CCH$\underline{C}$H$_3$CH$_3$-, methyl group facing turned away from carboxylic group).

Poly-3R-caranamide (**poly5-3R**)

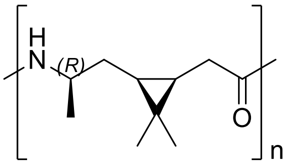

poly5-3R

^{1}H NMR (400 MHz, DCOOD): δ/ppm = 4.13 – 3.99 (1H, -NH-C$\underline{H}$CH$_3$-CH$_2$-, repeating unit), 2.55 – 2.35 (2H, -HN-CO-C$\underline{H_2}$-CH-, repeating unit), 1.77 – 1.60 (1H, -NH-CHCH$_3$-C$\underline{H_2}$-CH-, repeating unit), 1.54 – 1.39 (1H, -NH-CHCH$_3$-C$\underline{H_2}$-CH-, repeating unit), 1.32 – 1.22 (3H, -NH-CHC$\underline{H_3}$-CH$_2$-, repeating unit), 1.18 – 1.07 (3H, -CO-CH$_2$-CH-CCHCH$_3$C$\underline{H_3}$-, methyl group facing carboxylic group, repeating unit), 1.06 – 1.00 (3H, -NH-CHCH$_3$-CH-CCHC$\underline{H_3}$CH$_3$-, methyl group turned away from carboxylic group, repeating unit), 0.99 – 0.88 (1H, -CO-CH$_2$-C$\underline{H}$-, repeating unit), 0.76 – 0.64 (1H, -NH-CHCH$_3$-CH$_2$-C$\underline{H}$-, repeating unit).

^{13}C NMR (100 MHz, DCOOD): δ/ppm = 176.6 (-$\underline{C}$O-), 47.5 (-NH-$\underline{C}$HCH$_3$-), 31.3 (-CO-$\underline{C}$H$_2$-), 30.7 (-CHCH$_3$-$\underline{C}$H$_2$-), 28.2 (-CCHCH$_3$$\underline{C}H_3$-, methyl group facing carboxylic group), 23.3 (-CO-CH$_2$-$\underline{C}$H-), 21.9 (-CHCH$_3$-CH$_2$-$\underline{C}$H-), 19.3 (-CH$\underline{C}$H$_3$-), 17.9 (-$\underline{C}$CHCHCH$_3$CH$_3$-), 14.5 (-CCH$\underline{C}$H$_3$CH$_3$-, methyl group facing turned away from carboxylic group).

3S-caranlactam-3R-caranlactam-copolyamide (**copoly(5-3S·5-3R)**)

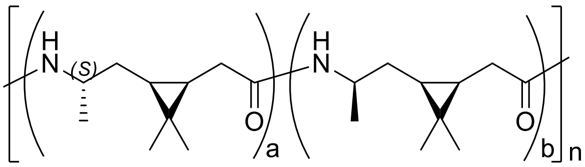

copoly(5-3S·5-3R)

^{1}H NMR (400 MHz, DCOOD): δ/ppm = 4.18 – 3.99 (1H, -NH-C*H*CH$_3$-CH$_2$-), 2.58 – 2.36 (2H, -HN-CO-C*H$_2$*-CH-), 1.77 – 1.64 (0.7 H$_{R\text{-isomer}}$, -NH-CHCH$_3$-C*H$_2$*-CH-), 1.63 – 1.53 (0.6 H$_{S\text{-isomer}}$, -NH-CHCH$_3$-C*H$_2$*-CH-), 1.52 – 1.41 (0.7 H$_{R\text{-isomer}}$, -NH-CHCH$_3$-C*H$_2$*-CH-), 1.33 – 1.23 (3H, -NH-CHC*H$_3$*-CH$_2$-), 1.17 – 1.07 (3H, -CO-CH$_2$-CH-CCHCH$_3$C*H$_3$*-), 1.06 – 1.00 (3H, -NH-CHCH$_3$-CH-CCHC*H$_3$*CH$_3$-), 0.99 – 0.89 (1H, -CO-CH$_2$-C*H*-), 0.80 – 0.65 (1H, -NH-CHCH$_3$-CH$_2$-C*H*-).

^{13}C NMR (100 MHz, DCOOD): δ/ppm = 176.6 (-*C*O-), 47.5 (-NH-C_RHCH$_3$-), 47.3 (-NH-C_SHCH$_3$-) 31.4 (-$C_{R,S}$O-*C*H$_2$-), 30.8 (-CHCH$_3$-$C_{R,S}$H$_2$-), 28.3 (-CCHCH$_3$$C_{R,S}H_3$-), 23.4 (-CO-CH$_2$-$C_{R,S}$H-), 22.0 (-CHCH$_3$-CH$_2$-$C_R$H-), 21.8 (-CHCH$_3$-CH$_2$-$C_S$H-), 19.6 (-CH$C_SH_3$-), 19.4 (-CH$C_RH_3$-), 17.9 (-$C_{R,S}$CHCHCH$_3CH_3$-), 14.8 (-CCH$C_SH_3CH_3$-), 14.6 (-CCH$C_RH_3CH_3$-).

3S-caranlactam-caprolactam-copolyamide (**copoly(5-3S·CL)**)

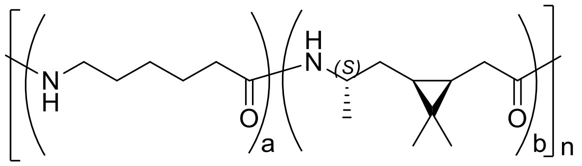

copoly(5-3S·CL)

In the ^{13}C-spectra, additional ^{13}C-signals were observed in close proximity to the expected signals. These signals can be interpreted as sign for a random built-in of small blocks of each monomer. The major ^{13}C-signal refers to the in-block carbons (+**5-3S**+**5-3S**+**5-3S**-; +**CL**+**CL**+**CL**+), whereas the minor signals are from the block-change units (+**CL**+**5-3S**+). A similar effect was not observed in the ^{1}H-spectra, probably due to the broad signals.

^{1}H NMR (400 MHz, DCOOD): δ/ppm = 4.17 – 4.01 (-NH-C*H*CH$_3$-CH$_2$-), 3.46 – 3.26 (-C*H$_2$*-NH-), 2.56 – 2.35 (-HN-CO-C*H$_2$*-CH-; -CO-C*H$_2$*-CH$_2$-), 1.85 – 1.51 (-NH-CHCH$_3$-C*H$_2$*-CH-, NH-CH$_2$-C*H$_2$*-, -CO-CH$_2$-C*H$_2$*-), 1.50 – 1.34 (CH$_2$-C*H$_2$*-CH$_2$-, 1.32 – 1.18 (-NH-CHC*H$_3$*-CH$_2$-), 1.16 – 1.06 (-CO-CH$_2$-CH-CCHCH$_3$C*H$_3$*-), 1.05 – 0.98 (-NH-CHCH$_3$-CH-CCHC*H$_3$*CH$_3$-), 0.96 – 0.86 (-CO-CH$_2$-C*H*-), 0.80 – 0.65 (-NH-CHCH$_3$-CH$_2$-C*H*-).

^{13}C NMR (100 MHz, DCOOD): δ/ppm =

176.7 (-*C*O-), 47.3 (-NH-*C*HCH$_3$-), 31.3 (-CO-*C*H$_2$-), 30.8 (-CHCH$_3$-*C*H$_2$-), 28.3 (-CCHCH$_3$*C*H$_3$-, methyl group facing carboxylic group), 23.3 (-CO-CH$_2$-*C*H-), 21.5 (-CHCH$_3$-CH$_2$-*C*H-, 19.5 (-CH*C*H$_3$-), 17.9 (-*C*CHCHCH$_3$CH$_3$-), 14.6 (-CCH*C*H$_3$CH$_3$-, methyl group facing turned away from carboxylic group). (from **5-3S**, major ^{13}C signals).

177.6 (-*C*O-), 40.3 (-NH-*C*H$_2$-), 35.6 (-CO-*C*H$_2$-CH$_2$-), 28.1 (-NH-CH$_2$-*C*H$_2$-), 26.0 (-CO-CH$_2$-*C*H$_2$-), 25.4 (-CH$_2$-*C*H$_2$-CH$_2$-). (from **CL**, major signals).

3S-caranlactam (**5-3S**)-laurolactam-copolyamide (**copoly(5-3S·LL)**)

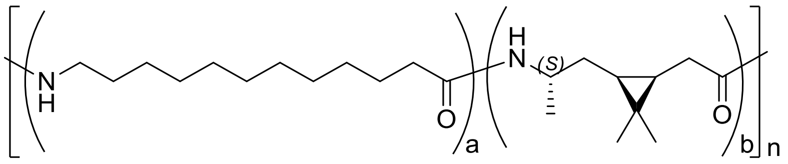

copoly(5-3S·LL)

In the ^{13}C-spectra, additional ^{13}C-signals were observed in close proximity to the expected signals. These signals can be interpreted as sign for a random built-in of small blocks of each monomer. The major ^{13}C-signal refers to the in-block carbons (+**5-3S**+**5-3S**+**5-3S**-; +**LL**+**LL**+**LL**+), whereas the minor signals are from the block-change units (+**LL**+**5-3S**+). A similar effect was not observed in the ^{1}H-spectra, probably due to the broad signals.

^{1}H NMR (400 MHz, DCOOD): δ/ppm = 4.18 – 4.03 (-NH-C*H*CH$_3$-CH$_2$-), 3.47 – 3.31 (-C*H$_2$*-NH-), 2.58 – 2.38 (-HN-CO-C*H$_2$*-CH-; -CO-C*H$_2$*-CH$_2$), 1.82 – 1.21 (-NH-CHCH$_3$-C*H$_2$*-CH-; -NH-CHC*H$_3$*-CH$_2$-; -C*H$_2$*-C*H$_2$*-), 1.19 – 1.10 (-CO-CH$_2$-CH-CCHCH$_3$C*H$_3$*-), 1.09 – 1.00 (-NH-CHCH$_3$-CH-CCHC*H$_3$*CH$_3$-), 0.99 – 0.87 (-CO-CH$_2$-C*H*-), 0.83 – 0.66 (-NH-CHCH$_3$-CH$_2$-C*H*-).

^{13}C NMR (100 MHz, DCOOD): δ/ppm =

177.1 (-*C*O-), 47.4 (-NH-*C*HCH$_3$-), 31.3 (-CO-*C*H$_2$-), 30.8 (-CHCH$_3$-*C*H$_2$-), 28.2 (-CCHCH$_3$*C*H$_3$-, methyl group facing carboxylic group), 23.3 (-CO-CH$_2$-*C*H-), 21.6 (-CHCH$_3$-CH$_2$-*C*H-, 19.5 (-CH*C*H$_3$-), 17.9 (-*C*CHCHCH$_3$CH$_3$-), 14.6 (-CCH*C*H$_3$CH$_3$-, methyl group facing turned away from carboxylic group). (from **5-3S**, major ^{13}C signals).

178.0 (-*C*O-), 40.7 (-NH-*C*H$_2$-), 35.5 (-CO-*C*H$_2$-CH$_2$-), 29.3 (2xC -*C*H$_2$-), 29.0 (-*C*H$_2$-), 28.8 (-*C*H$_2$-), 28.8 (-*C*H$_2$-), 28.5 (-*C*H$_2$-), 26.6 (-*C*H$_2$-), 25.9 (-*C*H$_2$-). (from **LL**, major signals).

4. Supplementary Notes

Supplementary Note 1. Single-crystal X-ray diffraction. For **5-3S** (CCDC 1938732), the integration of the data using a monoclinic unit cell yielded a total of 6057 reflections to a maximum Θ angle of 26.41° (0.80 Å resolution), of which 1959 were independent (average redundancy 3.092, completeness = 99.9 %, R_{int} = 2.65%, R_{sig} = 2.75%) and 1840 (93.93 %) were greater than 2σ(F^2). The final cell constants, Supplementary Table Supplementary **Table 1**8, are based upon the refinement of the XYZ-centroids of 4510 reflections above 20 σ(I) with 6.609° < 2Θ < 52.68°. The ratio of minimum to maximum apparent transmission was 0.862. The calculated minimum and maximum transmission coefficients (based on crystal size) are 0.9810 and 0.9960. The final anisotropic full-matrix least-squares refinement on F^2 with 117 variables converged at R1 = 3.00%, for the observed data and wR2 = 7.52% for all data. The goodness-of-fit was 1.081. The largest peak in the final difference electron density synthesis was 0.177 $e^-/Å^3$ and the largest hole was -0.157 $e^-/Å^3$ with an RMS deviation of 0.034 $e^-/Å^3$.
On the basis of the final model, the calculated density was 1.147 g/cm^3, and F(000) 368 e^-. For **5-3R** (CCDC 1938733 redetermination of CCDC 145220), the integration of the data using a monoclinic unit cell yielded a total of 25457 reflections to a maximum Θ angle of 28.31° (0.75 Å resolution), of which 4685 were independent (average redundancy 5.434, completeness = 99.7 %, R_{int}=2.96 %, R_{sig}=2.09 %) and 4541 (96.93 %) were greater than 2σ(F^2). The final cell constants, Supplementary Table Supplementary **Table 1**, are based upon the refinement of the XYZ-centroids of 9509 reflections above 2Θ σ(I) with 4.693° < 2Θ < 56.60°. The ratio of minimum to maximum apparent transmission was 0.922. The calculated minimum and maximum transmission coefficients (based on crystal size) are 0.6874 and 0.7457. The final anisotropic full-matrix least-squares refinement on F° with 232 variables converged at R1 = 2.89 %, for the observed data and wR2 = 7.74 % for all data. The goodness-of-fit was 1.081. The largest peak in the final difference electron density synthesis was 0.280 $e^-/Å^3$ and the largest hole was -0.155 $e^-/Å^3$ with an RMS deviation of 0.035 $e^-/Å^3$. On the basis of the final model, the calculated density was 1.171 g/cm^3, and F(000) 368 e^-.
Directional views of their structures were prepared as PLUTON periodic graphs, Supplementary Figures 32, 33 and 34.[8] The diffraction patterns obtained from **5-3S** and **5-3R** (from PXRD) showed narrow isolated Bragg reflexes without amorphous background, Supplementary Figures 31 (a) and (b).

Supplementary Note 2. Powder X-ray diffraction of poly5-3S. The diffraction patterns recorded from **poly5-3S** were characteristic of a semi-crystalline polymer, Supplementary Figure 31 (c). By scaling the amorphous reference patterns to match them at 2Θ values outside the range of Bragg peaks, we obtained *fc* = 0.43 from the ratios of the integrated intensities. Patterns from **poly5-3R** showed no distinct reflexes, save from residual monomer, Supplementary Figure 31 (d). Finding the structure of **poly5-3S** was impeded by the small number of distinct Bragg peaks. But, out of an initial selection of

13 tested unit cells and space groups, one met all three requirements for further assessment laid out in *Experimental*. The space group was Cambridge Structural Database entry 58438, Supplementary Table 17. The 50 SA runs performed on this structure then yielded a clear outcome: Three results presented distinctly lowest FOM. Of these, the one with the lowest CF simultaneously showed the most plausible atomic arrangement with regard to bonding angles, and the N-H···O bond arrangement. Directional views of its structure were prepared as periodic graphs, and as Oak Ridge thermal ellipsoid plots, Supplementary Figures 35,36, Figure 10.[8;9]

5. Supplementary References

[1] G. M. Sheldrick, “SHELXT – Integrated space-group and crystal-structure determination,” Acta Crystallographica Section A: Foundations and Advances, vol. 71, no. 1, pp. 3–8, 2015.

[2] G. M. Sheldrick, “Crystal structure refinement with SHELXL,” Acta Crystallographica Section C: Structural Chemistry, vol. 71, no. 1, pp. 3–8, 2015.

[3] C. B. Hübschle, G. M. Sheldrick, and B. Dittrich, “ShelXle: A Qt graphical user interface for shelxl,” Journal of Applied Crystallography, vol. 44, no. 6, pp. 1281–1284, 2011.

[4] A. Wilson, “International Tables for Crystallography, Vol. C, Tables 6.1. 1.4 (pp. 500–502), 4.2. 6.8 (pp. 219–222), and 4.2. 4.2 (pp. 193–199),” Kluwer Academic Publishers, Dordrecht, 1992.

[5] A. Altomare, N. Corriero, C. Cuocci, A. Falcicchio, A. Moliterni, and R. Rizzi, “Expo software for solving crystal structures by powder diffraction data: Methods and application,” Crystal Research and Technology, vol. 50, no. 9-10, pp. 737–742, 2015.

[6] A. Altomare, G. Campi, C. Cuocci, L. Eriksson, C. Giacovazzo, A. Moliterni, R. Rizzi, and P.-E. Werner, “Advances in powder diffraction pattern indexing: N- TREOR09,” *Journal of Applied Crystallography*, vol. 42, no. 5, pp. 768–775, 2009.

[7] J. Stewart, “MOPAC2016,” tech. rep., Stewart Computational Chemistry: Colorado Springs, Colorado, 2016.

[8] A. L. Spek, “PLATON SQUEEZE: A tool for the calculation of the disordered solvent contribution to the calculated structure factors,” Acta Crystallographica Section C: Structural Chemistry, vol. 71, no. 1, pp. 9–18, 2015.

[9] M. N. Burnett and C. K. Johnson, “ORTEP-III: Oak Ridge thermal ellipsoid plot program for crystal structure illustrations,” tech. rep., Oak Ridge National Laboratory report ORNL-6895, Tennessee, 1996.

[10] W. Astbury, “The hydrogen bond. The hydrogen bond in protein structure,” Transactions of the Faraday Society, vol. 36, pp. 871–880, 1940.

[11] W. L. Bragg, J. C. Kendrew, and M. F. Perutz, “Polypeptide chain configurations in crystalline proteins,” Proceedings of the Royal Society of London. Series A. Mathematical and Physical Sciences, vol. 203, no. 1074, pp. 321–357, 1950

[12] P. N. Stockmann, D. L. Pastoetter, M. Woelbing, C. Falcke, M. Winnacker, H. Strittmatter, V. Sieber, *Macromolecular rapid communications* **2019**, *40*, e1800903.
