# Supplementary material for: Biobased chiral semi-crystalline or amorphous high-performance polyamides and their scalable stereoselective synthesis
Source: Nat Commun. 2020 Jan 24;11:509. doi: 10.1038/s41467-020-14361-6 (PMC6981233; doi:10.1038/s41467-020-14361-6)
Supplement: Supplementary file 3 — Description of Additional Supplementary Files [file 41467_2020_14361_MOESM3_ESM.pdf]

Description of Additional Supplementary Files

File Name: Supplementary Data 1

Description: Crystallographic data of poly-3S-caranamide (poly5-3S) as .hkl file
